# Central and peripheral neuromuscular mechanisms underlying functional recovery heterogeneity in tibial plateau fractures

**DOI:** 10.1016/j.isci.2026.116490

**Published:** 2026-06-25

**Authors:** Zihao Sun, Gangqiang Du, Chao Liu, Hongzhen Du, Jianxin Sun, Baoju Wang, Bixuan Duan, Binbin Huang, Meng Guo, Lina Zhang, Pei Ma, Li Yu, Wei Li

**Affiliations:** 1 Department of Special Education and Rehabilitation, Binzhou Medical University, Yantai, Shandong, China; 2 First Clinical Medical College, Shandong University of Traditional Chinese Medicine, Jinan, Shandong, China; 3 Department of Sports Training, Tianjin University of Sport, Tianjin, China; 4 Department of Rehabilitation Medicine, Binzhou Medical University Hospital, Binzhou, Shandong, China; 5 Department of Trauma Orthopedics, Binzhou Medical University Hospital, Binzhou, Shandong, China

**Keywords:** Health sciences, Medicine, Medical specialty, Orthopedics

## Abstract

Functional recovery after tibial plateau fracture surgery shows substantial heterogeneity, with 30–40% of patients developing persistent limitations that extend beyond peripheral muscle deficits and may involve coupled central neural adaptations. We compared 32 patients with higher (Knee Injury and Osteoarthritis Outcome Score-Activities of Daily Living (KOOS-ADL) ≥80) and 32 with lower (KOOS-ADL ≤70) functional outcomes at 12 months post-surgery using synchronized three-dimensional motion capture, functional near-infrared spectroscopy, and surface electromyography during normal, dual-task, and balance-challenged walking. Routine gait revealed sensorimotor cortex hypoactivation and reduced affected-side ankle co-contraction in the lower-function group; dual-task walking exposed a marked affected-side ankle power deficit; and balance-challenged walking unmasked the largest discriminator, a 32.7% versus 7.3% knee coronal plane range-of-motion asymmetry (*d* = 2.54), accompanied by bilateral dorsolateral prefrontal cortex deactivation and reduced antagonist co-contraction. These coupled cortical and muscular control deficits define a bone-brain-muscle signature of lower functional recovery and support precision rehabilitation targeting patients most at risk.

## Highlights

• Balance-challenged walking reveals coronal knee motion asymmetry in lower recovery
• Lower-recovery patients show sensorimotor and prefrontal cortex hypoactivation
• Task-specific antagonist co-contraction deficits mark lower recovery
• Findings support a bone-brain-muscle framework for precision rehabilitation

## Graphical abstract

**Figure:**
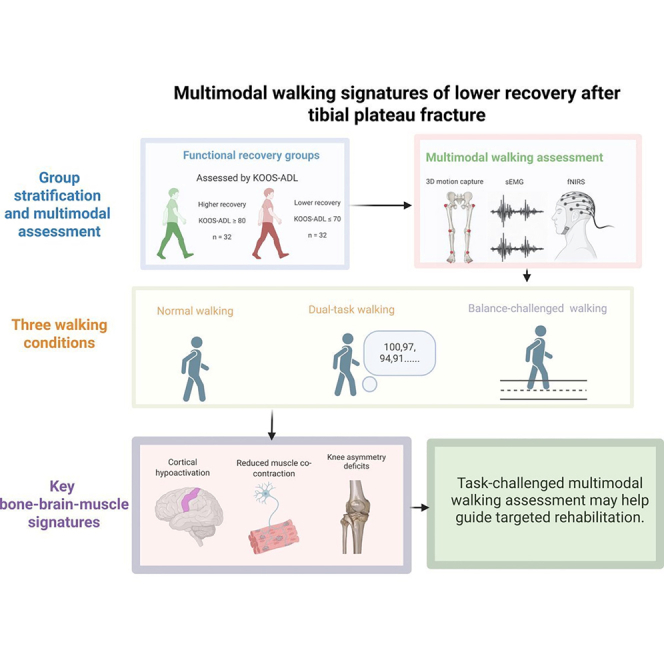

## Introduction

Tibial plateau fractures are severe intra-articular injuries, with an annual incidence of 10.3–26.9 per 100,000 individuals and a rising prevalence.^1^^,^^2^ Open reduction and internal fixation using locking plate systems remains the standard treatment and has improved fracture reduction and early mechanical stability.^3^ Nevertheless, functional recovery varies widely, and 30–40% of patients continue to experience long-term functional impairments despite surgical advances and structured rehabilitation.^4^^,^^5^

Postoperative recovery is commonly assessed using patient-reported measures such as the Knee Injury and Osteoarthritis Outcome Score-Activities of Daily Living (KOOS-ADL) subscale.^6^ Lower KOOS-ADL scores are consistently associated with difficulties in balance-intensive daily activities, including stair climbing, bathing, and heavy household tasks, which require integrated neuromuscular coordination rather than isolated load-bearing capacity.^7^ Notably, these limitations often persist even when radiographic healing is satisfactory, indicating that bony union alone does not ensure the restoration of functional neuromuscular control.

The mechanisms underlying this variability in functional outcomes remain poorly defined. It is unknown whether patients with differing recovery profiles adopt distinct cortical control strategies, muscle coordination patterns, or biomechanical adaptations during walking. This lack of mechanistic insight limits the development of targeted rehabilitation approaches, as clinicians cannot tailor interventions without clearly identifying the neuromuscular deficits associated with lower functional recovery.

Three-dimensional motion capture allows objective evaluation of gait biomechanics and has demonstrated persistent movement asymmetries more than two years after surgery.^8^ However, conventional gait assessments may fail to detect subtle impairments, as task-challenged conditions such as dual-task and balance-challenged walking can more effectively expose deficits in motor control.^9^^,^^10^ In parallel, functional near-infrared spectroscopy (fNIRS) enables the assessment of cortical activation during walking,^11^ and surface electromyography (sEMG) provides quantitative measures of peripheral muscle coordination.^12^ Several previous studies have begun to integrate multiple neurophysiological assessment modalities during gait. Caliandro et al. simultaneously measured prefrontal cortex activation using fNIRS and lower-limb muscle activation using sEMG during over-ground walking in patients with stroke.^13^ Kim et al. combined fNIRS with sEMG to examine age-related differences in cortical-muscular coupling during treadmill walking.^14^ Gramigna et al. systematically reviewed multimodal neuroimaging approaches combined with extended reality technologies for advanced gait analysis.^15^ However, no study has applied synchronized three-dimensional motion capture, fNIRS, and sEMG to characterize neuromuscular control profiles associated with divergent functional outcomes after tibial plateau fracture surgery. Current clinical evaluations primarily emphasize peripheral musculoskeletal function and often neglect central neural adaptations following trauma and immobilization. Distinguishing whether persistent limitations arise from peripheral muscle deficits or altered central motor control is essential for guiding rehabilitation.

**Figure 1:**
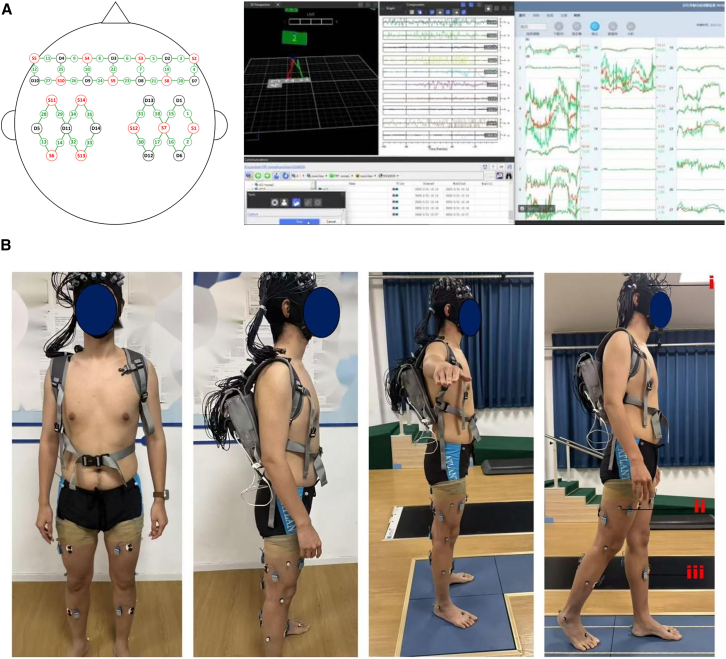
Multimodal gait analysis experimental setup (A) The 35-channel fNIRS acquisition system, with optodes covering bilateral prefrontal and sensorimotor cortices according to the 10–20 system (sources in red, detectors in green); the lower panel shows the real-time oxygenated-hemoglobin (HbO_2_) signal-acquisition interface. Motion capture, sEMG, and fNIRS were synchronized with <10 μs precision using a Vicon Lock Lab unit. (B) Lateral view of the integrated measurement configuration during balance-challenged walking on a 10-cm narrow base, showing the placement of (1) the fNIRS cap, (2) reflective motion-capture markers (16-marker Plug-in-Gait lower-body set), and (3) bilateral sEMG electrodes. Facial features have been masked to protect identity; written informed consent for the publication of the image in (B) was nonetheless obtained from the individual depicted.

Accordingly, we compared patients with higher (KOOS-ADL ≥80) and lower (KOOS-ADL ≤70) functional outcomes at 12 months post-surgery using synchronized multimodal assessments (Figure 1) to: (1) identify biomechanical impairments under progressively challenging walking tasks; (2) characterize cortical activation patterns and network reorganization; and (3) evaluate muscle coordination strategies and brain-muscle coupling. By defining neuromuscular signatures associated with reduced functional recovery, this study aims to inform the development of targeted, individualized rehabilitation strategies.

## Results

### Baseline characteristics

The two groups showed comparable demographic and clinical characteristics (Table 1). We observed no significant differences in age, sex, BMI, or time since surgery (all *p* > 0.05; Table 1). As intended, KOOS-ADL scores differed markedly between groups (83.32 ± 2.47 vs. 61.31 ± 5.09, *p* < 0.001), consistent with the predefined stratification thresholds. Comparisons with healthy controls are presented in Table S1.

**Table 1:** Baseline demographic and clinical characteristics of the study participants

Category	Characteristic	Higher functional outcome (*n* = 32)	Lower functional outcome (*n* = 32)	*p* value
Demographics	age (years)	48.3 ± 8.7	49.2 ± 9.4	0.686
Demographics	sex, male, *n* (%)	20 (62.5%)	19 (59.4%)	0.795
Demographics	height (m)	1.68 ± 0.08	1.67 ± 0.09	0.621
Demographics	weight (kg)	65.4 ± 8.2	64.8 ± 7.9	0.763
Demographics	BMI (kg/m^2^)	23.2 ± 1.8	23.4 ± 1.6	0.642
Clinical characteristics	affected side, left, *n* (%)	15 (46.9%)	16 (50.0%)	0.823
Clinical characteristics	time since surgery (months)	12.1 ± 0.4	12.0 ± 0.5	0.371
Clinical characteristics	fracture type (Schatzker II), *n* (%)	32 (100%)	32 (100%)	–
Clinical characteristics	MTPA (°)	87.30 ± 2.38	87.40 ± 2.66	0.866
Functional outcome	**KOOS pain score (0–100)**	**77.78 (75.00, 80.56)**	**75.00 (72.22, 80.56)**	**0.199**
Functional outcome	**KOOS-ADL score (0–100)**	**83.32** ± **2.47**	**61.31** ± **5.09**	< **0.001∗∗∗**

Data are presented as means ± SD, median (Q1, Q3), or *n* (%). ∗∗∗*p* < 0.001.

Higher functional outcome: KOOS-ADL ≥80; lower functional outcome: KOOS-ADL ≤70.

BMI: body mass index; KOOS-ADL: knee injury and osteoarthritis outcome score – activities of daily living subscale; MTPA: medial tibial plateau angle.

Analysis of 17 individual ADL items (Table S2) showed that deficits were most pronounced in activities requiring dynamic balance, including stair descent (0.31 ± 0.47 vs. 2.72 ± 0.92, *r* = 0.86), getting in and out of the bath (0.62 ± 0.49 vs. 2.00 ± 0.72, *r* = 0.77), and heavy domestic tasks (0.69 ± 0.54 vs. 1.97 ± 0.82, *r* = 0.70), whereas differences in static activities were smaller. These patterns supported the rationale for employing task-challenged walking paradigms. Visual analog scale (VAS) pain scores at testing were minimal in both patient groups and did not differ significantly (0.31 ± 0.46 vs. 0.47 ± 0.61, *p* = 0.357; Table S1). Radiographic assessment at 12 months revealed no between-group difference in medial tibial plateau angle (MTPA: 87.30° ± 2.38 vs. 87.40° ± 2.66, *p* = 0.866, *d* = 0.042; Table 1), confirming comparable tibial plateau alignment and excluding clinically significant articular surface subsidence as a confounder.

### Task-challenge paradigms progressively amplify biomechanical deficits

Between-group comparisons of knee coronal plane ROM symmetry showed progressively larger differences as task difficulty increased (Table 2). During normal walking, the lower-function group exhibited greater asymmetry than the higher-function group (14.12% [11.47, 25.54] vs. 6.16% [2.67, 8.37], *p* < 0.001, *r* = 0.76). This difference increased substantially during dual-task walking (32.83 ± 20.74% vs. 7.77 ± 4.83%, *p* < 0.001, *d* = 1.66) and was most pronounced during balance-challenged walking (32.74 ± 13.09% vs. 7.29 ± 5.42%, *p* < 0.001, *d* = 2.54). Effect sizes increased from large to very large, indicating high sensitivity of knee coronal plane control to task challenge (Tables S3–S8).

**Table 2:** Key biomechanical parameters differentiating functional recovery trajectories across task conditions

Category	Parameter	Normal walking, LFO	Normal walking, HFO	Normal walking, *P*	Normal walking, ES	Dual-task walking, LFO	Dual-task walking, HFO	Dual-task walking, *P*	Dual-task walking, ES	Balance-challenged walking, LFO	Balance-challenged walking, HFO	Balance-challenged walking, *P*	Balance-challenged walking, ES
Primary outcome: ROM asymmetry (%)	**knee coronal plane**	**14.12 (11.47, 25.54)**	**6.16 (2.67, 8.37)**	< **0.001**	**0.76**^b^	**32.83** ± **20.74**	**7.77** ± **4.83**	< **0.001**	**1.66**^a^	**32.74** ± **13.09**	**7.29** ± **5.42**	< **0.001**	**2.54**^a^
Primary outcome: ROM asymmetry (%)	knee transverse plane	18.34 (11.28, 35.76)	40.83 (25.08, 56.83)	**0.01**	0.32^b^	15.99 (7.77, 32.54)	26.49 (16.98, 43.73)	**0.03**	0.28^b^	21.03 ± 14.65	25.12 ± 18.76	0.34	0.24^a^
Secondary outcome: joint power (W/kg)	**ankle joint power**	2.80 (2.26, 3.09)	3.03 (2.65, 3.26)	0.11	0.20^b^	**1.06 (0.88, 2.48)**	**3.07 (2.53, 3.60)**	< **0.001**	**0.57**^b^	2.81 (2.53, 3.10)	3.22 (2.73, 3.93)	**0.048**	0.25^b^
Secondary outcome: joint power (W/kg)	knee joint power	0.50 (0.33, 0.69)	0.65 (0.53, 1.31)	**0.011**	0.32^b^	0.56 (0.41, 0.66)	0.55 (0.43, 0.89)	0.63	0.06^b^	0.60 (0.44, 0.83)	0.45 (0.31, 1.06)	0.22	0.15^b^
Spatiotemporal (balance only)	cadence (steps/min)	–	–	–	–	–	–	–	–	**95.71** ± **10.81**	**106.77** ± **10.77**	< **0.001**	**1.03**^a^
Spatiotemporal (balance only)	stride length (m)	–	–	–	–	–	–	–	–	**1.23** ± **0.06**	**1.30** ± **0.06**	< **0.001**	**1.23**^a^
Spatiotemporal (balance only)	walking speed (m/s)	–	–	–	–	–	–	–	–	**0.92 (0.89, 1.06)**	**1.20 (1.10, 1.27)**	< **0.001**	**0.59**^b^

Data are presented as means ± SD or median (Q1, Q3).

Bonferroni corrected α = 0.0125 for normal/dual-task conditions (4 comparisons) and α = 0.007 for balance condition (7 comparisons). Bold values indicate findings significant after correction.

LFO, lower functional outcome (KOOS-ADL ≤70); HFO: higher functional outcome (KOOS-ADL ≥80); ES: effect size; ROM: range of motion; W/kg: watts per kilogram.

a Cohen’s d

b r value. Bold values indicate primary findings.

Spatiotemporal gait parameters did not differ between groups during normal walking (all *p* > 0.05, Tables S9–S11). In contrast, balance-challenged walking revealed clear deficits in the lower-function group, including reduced cadence (95.71 ± 10.81 vs. 106.77 ± 10.77 steps/min, *p* < 0.001, *d* = 1.03), shorter stride length (1.23 ± 0.06 vs. 1.30 ± 0.06 m, *p* < 0.001, *d* = 1.23), and slower walking speed (0.92 vs. 1.20 m/s, *p* < 0.001, *r* = 0.59). Similar declining trends were observed in the unaffected limb, suggesting impaired global postural control rather than isolated dysfunction of the affected side.

Joint power analysis identified affected-side ankle power as the most discriminative biomechanical variable (Table 2). During dual-task walking, the lower-function group showed an approximately 65% reduction in ankle power compared with the higher-function group (1.06 [0.88, 2.48] vs. 3.07 [2.53, 3.60] W/kg, *p* < 0.001, *r* = 0.572), reflecting markedly reduced plantarflexor power generation during push-off (Tables S12–S34).

### Sensorimotor cortex hypoactivation with prefrontal exhaustion

Cortical activation showed two clear and complementary patterns across tasks (Table 3). First, the sensorimotor cortex exhibited consistent hypoactivation in the lower-function group under all walking conditions. During normal walking, significant between-group differences were observed in the S6–D5 channel (primary somatosensory cortex, BA 1–3; −0.014 ± 0.020 vs. 0.005 [−0.014, 0.024] mmol/L·mm, *p* = 0.002, *r* = 0.387). Similar reductions were detected in S6–D11 (BA 3, 1, 6; *p* = 0.001, *r* = 0.408), S7–D1 (supplementary motor area (SMA); *p* = 0.010, *r* = 0.321), and S14–D11 (premotor cortex; *p* = 0.003, *d* = 0.766). This stable pattern across tasks indicates persistent impairment within core sensorimotor control networks.

**Table 3:** Cortical activation patterns (ΔHbO_2_, mmol/L·mm) across task conditions

Category	Channel (brain region)	Normal walking, LFO	Normal walking, HFO	Normal walking, *P*	Normal walking, ES	Dual-task walking, LFO	Dual-task walking, HFO	Dual-task walking, *P*	Dual-task walking, ES	Balance-challenged walking, LFO	Balance-challenged walking, HFO	Balance-challenged walking, *P*	Balance-challenged walking, ES
Sensorimotor cortex	**S6**–**D5 (BA 1/2/3)**	**−0.014** ± **0.020**	**0.005 (**−**0.014, 0.024)**	**0.002**	**0.39**^b^	−0.008 ± 0.031	0.029 ± 0.046	< **0.001**	0.94^a^	0.004 (−0.040, 0.026)	0.013 (−0.014, 0.043)	0.20	0.33^a^
Sensorimotor cortex	**S6**–**D11 (BA 3/1/6)**	**−0.022** ± **0.028**	**0.001 (**−**0.009, 0.017)**	**0.001**	**0.41**^b^	0.007 ± 0.051	0.022 ± 0.042	0.20	0.32^a^	0.002 (−0.022, 0.036)	0.015 (−0.005, 0.039)	0.86	0.04^a^
Sensorimotor cortex	S7–D1 (SMA, BA 6)	−0.009 ± 0.027	0.002	**0.010**	0.32^b^	–	–	–	–	−0.008 ± 0.032	0.016 ± 0.041	**0.011**	0.66^a^
Sensorimotor cortex	S14–D11 (PMC, BA 6)	−0.020 ± 0.025	0.002 ± 0.034	**0.003**	0.77^a^	–	–	–	–	−0.007 ± 0.035	0.019 ± 0.038	**0.007**	0.70^a^
Prefrontal cortex	S3–D2 (PFA, BA 10)	0.021 ± 0.041	−0.001 ± 0.039	**0.029**	0.56^a^	–	–	–	–	–	–	–	–
Prefrontal cortex	S3–D3 (PFA, BA 10)	–	–	–	–	0.003 ± 0.038	0.048 ± 0.057	< **0.001**	0.93^a^	–	–	–	–
Prefrontal cortex	**S8**–**D8 (DLPFC, BA 9/46)**	0.006 (−0.012, 0.022)	0.006 (−0.020, 0.029)	0.61	0.06^b^	0.001 (−0.025, 0.033)	−0.003 (−0.023, 0.046)	0.80	0.06^b^	**−0.022 (−0.073, 0.008)**	**0.025 (0.005, 0.056)**	< **0.001**	**1.06**^a^
Prefrontal cortex	**S9**–**D9 (DLPFC, BA 9/46)**	0.008 ± 0.022	0.004 ± 0.030	0.51	0.17^a^	0.028 (−0.009, 0.027)	0.015 (−0.006, 0.025)	0.63	0.12^b^	**−0.009 (−0.027, 0.009)**	**0.012 (-0.002, 0.043)**	**0.001**	**0.85**^a^

Data are presented as means ± SD or median (Q1, Q3).

Bonferroni corrected α = 0.00143 for 35-channel comparisons per condition. Bold values indicate findings significant after correction.

LFO, lower functional outcome; HFO: higher functional outcome; BA: Brodmann area; DLPFC: dorsolateral prefrontal cortex; HbO_2_, oxygenated hemoglobin; PFA: polar frontal area; PMC: premotor cortex; SMA: supplementary motor area.

– indicates non-significant or not reported for that condition.

a Cohen’s d

b r value. Bold values indicate key findings.

Second, prefrontal activation demonstrated strong task dependence, shifting from compensatory recruitment to apparent functional exhaustion. During normal walking, the lower-function group showed increased polar frontal activation (S3–D2, BA 10; 0.021 ± 0.041 vs. −0.001 ± 0.039 mmol/L·mm, *p* = 0.029, *d* = 0.560), indicating greater reliance on cognitive control even during simple gait. Dual-task walking further amplified these differences, with larger between-group differences across multiple prefrontal channels (S3–D3: *p* < 0.001, *d* = 0.932; S4–D3: *p* = 0.003, *r* = 0.772).

In contrast, balance-challenged walking elicited a reversal of this pattern. The lower-function group demonstrated marked dorsolateral prefrontal cortex (DLPFC) hypoactivation at peak executive demand. Specifically, S8–D8 (BA 9/46) showed −0.022 [−0.073, 0.008] vs. 0.025 [0.005, 0.056] mmol/L·mm (*p* < 0.001, *d* = 1.055), with comparable reductions in S9–D9 (*p* = 0.001, *d* = 0.853). This failure to sustain DLPFC activation under high task demand suggests limited cognitive-motor integration capacity once compensatory resources are exceeded (Tables S35–S37).

### Cortical network reorganization reveals compensatory control strategies

**Figure 2:**
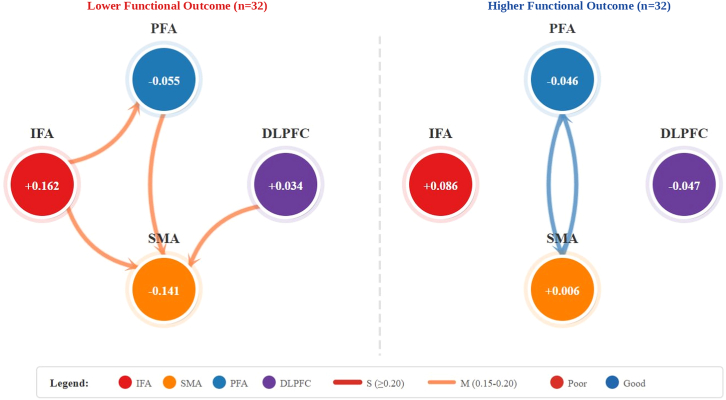
Granger causality networks during balance-challenged walking Left: lower-function group (*n* = 32) shows IFA-dominated control with the supplementary motor area (SMA) as the primary receiver of directed information flow. Right: higher-function group (*n* = 32) shows sparser and more efficient connectivity. Node values indicate net information flow (outflow − inflow). Edge widths are proportional to Granger causality magnitude. Directional effects were tested with F-tests on vector-autoregressive models (optimal lag order selected by AIC, range 2–4) and Bonferroni-corrected within each condition.

Granger causality analysis identified clear differences in cortical network organization between groups across walking tasks (Figures 2; S1–S3). During normal walking, the lower-function group showed increased inferior frontal area (IFA) drive (net flow: +0.213 vs. +0.079) and greater network connectivity (5 vs. 2 connections). During dual-task walking, the SMA became the dominant driver in the lower-function group, with a marked increase in strong connections (7 vs. 1). During balance-challenged walking, SMA shifted from a primary driver to a primary receiver (net flow: +0.100 to −0.141), indicating reduced automatic motor control and greater dependence on top-down prefrontal regulation. In contrast, the higher-function group maintained more stable and efficient networks with fewer but effective connections (Tables S38–S40).

### Muscle coordination deficits extend bilaterally

**Figure 3:**
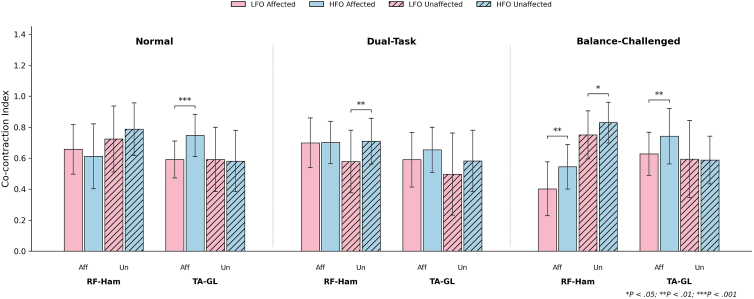
Muscle co-contraction indices across walking conditions Comparison of tibialis anterior-gastrocnemius lateralis (TA-GL) and rectus femoris-hamstring (RF-Ham) antagonist co-contraction between the lower (LFO, *n* = 32; pink) and higher (HFO, *n* = 32; light blue) functional outcome groups. For each muscle pair, affected-side bars (solid fill) and unaffected-side bars (cross-hatched) are displayed adjacent to facilitate bilateral comparison across normal walking, dual-task walking, and balance-challenged walking. Data are presented as mean ± SD. Between-group comparisons used independent-samples t-tests (for normally distributed data) or Mann-Whitney U tests (for non-normally distributed data). The prespecified Bonferroni threshold for the eight co-contraction comparisons within each condition was α = 0.006. Asterisks above bracketed bar pairs denote uncorrected between-group P levels (∗*p* < 0.05, ∗∗*p* < 0.01, and ∗∗∗*p* < 0.001); Bonferroni-adjusted significance calls are reported in Table S52.

The lower-function group exhibited reduced antagonist muscle co-contraction across tasks (Table 4; Figure 3). During normal walking, affected-side tibialis anterior-gastrocnemius lateralis (TA-GL) co-contraction was significantly lower (0.591 ± 0.119 vs. 0.747 ± 0.136, *p* < 0.001, *d* = 1.23). During balance-challenged walking, deficits extended bilaterally, affecting affected-side rectus femoris-hamstrings (RF-Ham; 0.402 ± 0.174 vs. 0.544 ± 0.144, *p* = 0.001, *d* = 0.89), affected-side TA-GL (*p* = 0.006, *d* = 0.71), and unaffected-side RF-Ham (*p* = 0.030, *d* = 0.56). During dual-task walking, unaffected-side RF-Ham co-contraction was also reduced (*p* = 0.004, *d* = 0.74). These results indicate bilateral coordination impairments under increased task demands (Tables S41–S48).

**Table 4:** Muscle co-contraction indices across task conditions

Category	Muscle pair/side	Normal walking, LFO	Normal walking, HFO	Normal walking, *P*	Normal walking, ES	Dual-task walking, LFO	Dual-task walking, HFO	Dual-task walking, *P*	Dual-task walking, ES	Balance-challenged walking, LFO	Balance-challenged walking, HFO	Balance-challenged walking, *P*	Balance-challenged walking, ES
Ankle: TA-GL	**affected side**	**0.591** ± **0.119**	**0.747** ± **0.136**	< **0.001**	**1.23**^a^	0.590 ± 0.176	0.654 ± 0.146	0.120	0.39^a^	0.628 ± 0.139	0.742 ± 0.179	**0.006**	0.71^a^
Ankle: TA-GL	unaffected side	0.592 ± 0.207	0.581 ± 0.198	0.823	0.06^a^	0.497 ± 0.265	0.582 ± 0.198	0.148	0.37^a^	0.594 ± 0.249	0.588 ± 0.154	0.920	0.03^a^
Knee: RF-Ham	**affected side**	0.657 ± 0.161	0.612 ± 0.209	0.340	0.24^a^	0.699 ± 0.161	0.701 ± 0.137	0.967	0.01^a^	**0.402** ± **0.174**	**0.544** ± **0.144**	**0.001**	**0.89**^a^
Knee: RF-Ham	**unaffected side**	0.724 ± 0.213	0.787 ± 0.170	0.199	0.33^a^	**0.578** ± **0.202**	**0.709** ± **0.148**	**0.004**	**0.74**^a^	0.750 ± 0.155	0.830 ± 0.131	0.030	0.56^a^

Data are presented as means ± SD. Co-contraction index range: 0–1 (higher values indicate greater antagonist muscle coordination).

Bonferroni corrected α = 0.006 for 8 comparisons per condition (4 muscle pairs × 2 sides). Bold values indicate findings significant after correction.

LFO, lower functional outcome (KOOS-ADL ≤70); HFO: higher functional outcome (KOOS-ADL ≥80).

ES, effect size; RF-Ham: rectus femoris-hamstring; TA-GL: tibialis anterior-gastrocnemius lateralis.

a Cohen’s d. Bold values indicate significant findings.

### Brain-muscle coupling reveals compensatory strategies

**Figure 4:**
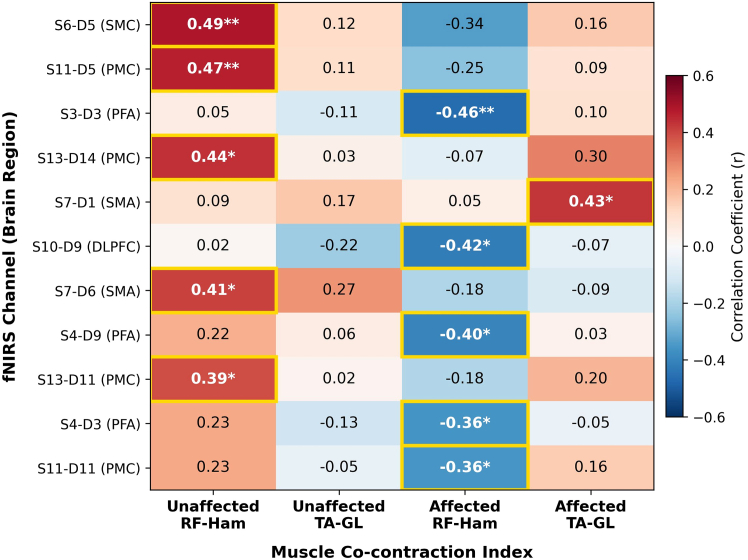
Brain-muscle coupling during balance-challenged walking in the lower functional outcome group Heatmap of correlations between channel-level cortical oxygenation (ΔHbO_2_) and antagonist muscle co-contraction indices in the lower-function group (*n* = 32). Strongest correlations: S6–D5 (primary somatosensory cortex) and S11–D5 (premotor cortex) with unaffected-side RF-Ham (r = 0.49 and r = 0.47); S3–D3 (polar frontal area) with affected-side RF-Ham (r = −0.46). Correlation method (Pearson’s r or Spearman’s ρ) was selected channel-by-channel by the Shapiro-Wilk normality test. Gold borders mark uncorrected *p* < 0.05, ∗*p* < 0.05, and ∗∗*p* < 0.01. None of the correlations survived FDR correction (Benjamini-Hochberg, q < 0.05) across 140 channels × muscle pairs, and the analysis is therefore reported as exploratory.

Correlation analyses identified significant associations between cortical activation and muscle co-contraction in the lower-function group (Table 5). During balance-challenged walking, activation in the sensorimotor cortex channel S6–D5 positively correlated with unaffected-side RF-Ham co-contraction (*r* = 0.49, *p* = 0.005), indicating that higher motor control demands were accompanied by increased muscle co-contraction. Additional significant correlations were observed in premotor cortex (S11–D5: *r* = 0.47, *p* = 0.007) and prefrontal areas (S3–D3: *r* = −0.46, *p* = 0.008) (Figure 4).

**Table 5:** Correlations between cortical activation and muscle co-contraction indices in the lower functional outcome group

Category	Channel	Brain region	Muscle pair	Side	*r*	*p* value
Balance-challenged walking	S6–D5	SMC (BA 1/2/3)	RF-Ham	unaffected	0.487	0.005∗∗
Balance-challenged walking	S11–D5	PMC (BA 6)	RF-Ham	unaffected	0.466	0.007∗∗
Balance-challenged walking	S3–D3	PFA (BA 10)	RF-Ham	affected	−0.460	0.008∗∗
Balance-challenged walking	S13–D14	PMC (BA 6)	RF-Ham	unaffected	0.440	0.012∗
Balance-challenged walking	S7–D1	SMA (BA 6)	TA-GL	affected	0.431	0.014∗
Normal walking	S2–D7	IFA (BA 45)	TA-GL	unaffected	−0.504	0.003∗∗
Normal walking	S6–D5	SMC (BA 1/2/3)	RF-Ham	unaffected	0.473	0.006∗∗
Normal walking	S11–D5	PMC (BA 6)	TA-GL	affected	0.473	0.006∗∗
Dual-task walking	S11–D11	PMC (BA 6)	RF-Ham	affected	0.460	0.008∗∗

Due to the exploratory nature of brain-muscle coupling analysis, correlations are reported at uncorrected *p* < 0.05. None survived FDR correction (q < 0.05) for 140 comparisons per condition. Correlation method (Pearson’s r or Spearman’s ρ) determined by the Shapiro-Wilk normality test.

DLPFC, dorsolateral prefrontal cortex; IFA: inferior frontal area; PFA: polar frontal area; PMC: premotor cortex; SMA: supplementary motor area; SMC: sensorimotor cortex; BA: Brodmann area; RF-Ham: rectus femoris-hamstring; TA-GL: tibialis anterior-gastrocnemius lateralis.

∗*p* < 0.05.

∗∗*p* < 0.01.

∗∗∗*p* < 0.001.

Importantly, these associations predominantly involved the unaffected limb rather than the affected limb, suggesting that patients with lower functional outcomes compensate for impaired coordination on the injured side by increasing prefrontal-driven neuromuscular control of the contralateral limb. This pattern indicates that compensation relies on active cortical regulation rather than purely spinal or reflex-based mechanisms (Table S49).

## Discussion

This study provides cross-sectional evidence consistent with the hypothesis that lower functional recovery after tibial plateau fracture surgery extends beyond peripheral muscle deficits and may involve central neural maladaptation, characterized by cognitive resource depletion and inefficient cortical-muscular coupling.

Knee coronal plane asymmetry differed between groups across all task conditions, with effect sizes of *r* = 0.76 (single-task walking), *d* = 1.66 (dual-task walking), and *d* = 2.54 (balance-challenged walking). The largest effect emerged under balance challenge, demonstrating that task-challenge paradigms effectively reveal motor control impairments that remain undetected during standard gait assessment, consistent with the attentional resource competition hypothesis.^9^ The 65% reduction in ankle power during dual-task walking identifies a specific biomechanical vulnerability with clear relevance for targeted rehabilitation. Together, these findings indicate that routine clinical gait assessments may underestimate persistent deficits and support incorporating balance-challenged walking tasks into postoperative follow-up.

The observed cortical activation patterns provide important mechanistic insight into lower functional recovery. Persistent sensorimotor hypoactivation across all tasks indicates reduced efficiency of primary motor circuits, likely reflecting altered cortical representation and diminished descending motor drive after prolonged immobilization and disrupted sensory input. The shift from prefrontal hyperactivation during simple tasks to bilateral DLPFC deactivation during balance-challenged walking reflects cognitive resource depletion when task demands exceed available capacity. Similar patterns have been reported in older adults^16^ and individuals with Parkinson’s disease,^17^ suggesting that orthopedic trauma may induce widespread central nervous system adaptations beyond the injured limb. Together, findings across biomechanics, cortical network organization, and muscle coordination support a “bone-brain-muscle coordination” framework for explaining heterogeneity in functional recovery. This integrated perspective indicates that rehabilitation after tibial plateau fracture should extend beyond joint-focused strategies to address interacting musculoskeletal, neuromuscular, and central neural deficits. The observed sensorimotor cortex-muscle coupling further demonstrates that peripheral motor execution is actively regulated by higher cortical centers, underscoring the need to integrate neurorehabilitation with conventional orthopedic care.

Persistent pain does not appear to account for the compensatory strategies observed in the lower-function group. Fuzier et al. reported that approximately half of patients experience persistent pain three months after orthopedic surgery.^18^ However, in the present study, KOOS Pain subscale scores did not differ between groups (median: 77.78 [75.00, 80.56] vs. 75.00 [72.22, 80.56], *p* = 0.199; Tables 1 and S50), with both groups reporting relatively low pain at 12 months post-surgery. Similarly, VAS scores at the time of testing were minimal and comparable between groups (0.31 ± 0.46 vs. 0.47 ± 0.61, *p* = 0.357). The neuromuscular deficits we identified, including sensorimotor cortex hypoactivation, reduced antagonist co-contraction, and prefrontal resource depletion, therefore appear to reflect central and peripheral motor control maladaptation rather than ongoing pain. The KOOS Symptoms subscale did differ between groups (67.41 ± 5.42 vs. 78.91 ± 5.83, *p* < 0.001), but this difference was driven by self-reported range-of-motion limitations rather than inflammatory symptoms such as swelling or morning stiffness (Table S51), consistent with the biomechanical deficits identified through objective gait analysis.

Structural factors, particularly gradual articular surface subsidence (sintering), are unlikely to account for the increased coronal plane knee motion observed in the lower-function group. In Schatzker Type II fractures, cancellous bone defects may lead to progressive loss of lateral tibial plateau height during healing, potentially resulting in secondary coronal plane instability. To address this concern, we measured the MTPA on anteroposterior knee radiographs at 12 months post-surgery in all 64 patients. The MTPA did not differ between groups (87.40° ± 2.66 vs. 87.30° ± 2.38, *p* = 0.866, *d* = 0.042), with all values within the normal physiological range (82–93°). Combined with our exclusion criteria requiring postoperative articular step-off ≤2 mm and lateral angulation ≤5°, these findings exclude clinically significant sintering as a confounding factor and support the interpretation that the increased coronal plane ROM asymmetry reflects neuromuscular control deficits rather than bony malalignment.

The positive association between sensorimotor cortex activation and unaffected-side muscle co-contraction (*r* = 0.49) provides direct evidence of motor cortical involvement in compensatory motor strategies. When automatic sensorimotor control is compromised, patients appear to preserve stability through cortically mediated contralateral muscle coordination. This interlimb coordination mechanism^19^ highlights the importance of targeting both affected and unaffected limbs during rehabilitation.

Patients with lower functional outcomes after tibial plateau fracture surgery demonstrate multilevel neuromuscular impairments, including marked knee coronal plane ROM asymmetry (*d* = 2.54 under balance challenge), reduced affected-side ankle power (65% reduction during dual-task walking), diminished antagonist muscle co-contraction (TA-GL: *d* = 1.23; RF-Ham: *d* = 0.89), persistent sensorimotor cortex hypoactivation, and bilateral DLPFC deactivation under high control demands. Task-challenge paradigms, particularly balance-challenged walking, effectively reveal deficits not detected by standard gait assessment. Compensatory strategies involve motor cortex-regulated contralateral muscle coordination (*r* = 0.49). Together, these results identify biomechanical, neuromuscular, and neural biomarkers for early risk stratification and support targeted rehabilitation within a bone-brain-muscle coordination framework.

These findings have direct clinical relevance. For assessment, balance-challenged walking should complement standard gait analysis to expose latent impairments. TA-GL and RF-Ham co-contraction indices may serve as peripheral biomarkers, while DLPFC and sensorimotor cortex activation patterns provide neural indicators for identifying patients at risk of lower functional recovery. For rehabilitation, interventions should address multiple levels, including: (1) knee coronal plane stability training; (2) ankle plantarflexor power enhancement; (3) antagonist coordination training to restore efficient co-contraction; and (4) dual-task training to improve motor automaticity and reduce excessive prefrontal reliance. Neuromodulation targeting the DLPFC or sensorimotor cortex warrants investigation in future trials.

For future studies requiring repetitive assessment during rehabilitation, we recommend balance-challenged walking as the primary task paradigm. In this study, balance-challenged walking demonstrated superior discriminative capacity across multiple domains: knee coronal plane control (*d* = 2.54), bilateral co-contraction deficits, cortical deactivation patterns, and spatiotemporal parameters. While dual-task walking uniquely identified affected-side ankle power deficits (*r* = 0.572), balance-challenged walking captured a broader spectrum of neuromuscular impairments within a single assessment. Additionally, balance-challenged walking is more practical for repeated clinical implementation, as it requires only a tape-marked path and does not depend on cognitive task calibration, which may introduce variability across sessions.

### Limitations of the study

Several limitations should be considered. First, the cross-sectional design limits causal inference regarding the identified neuromuscular deficits. Second, the restriction to Schatzker type II fractures may limit generalizability to other fracture patterns. Third, the synchronized multimodal protocol used here is resource-intensive and not directly suited to routine clinical rehabilitation. Future work will develop simplified assessment protocols based on the most discriminative measures identified here, examine when the bone-brain-muscle pattern of lower functional recovery emerges through longitudinal follow-up, and test whether targeted neuromuscular training can accelerate recovery in higher-risk patients.

## Resource availability

### Lead contact

Further information and requests for resources should be directed to and will be fulfilled by the lead contact, Wei Li (yishengliwei@163.com).

### Materials availability

This study did not generate new unique reagents.

### Data and code availability

• All de-identified individual-participant datasets supporting the findings of this study, including processed three-dimensional kinematic and kinetic gait variables, sEMG co-contraction indices, channel-level fNIRS oxygenated hemoglobin time series, KOOS subscale scores, and medial tibial plateau angle measurements, have been deposited at Zenodo and are publicly available at https://doi.org/10.5281/zenodo.20339370. The dataset DOI is also listed in the Key Resources Table. Raw video recordings, raw motion-capture marker trajectories, and any imaging data that could be linked back to an individual participant are not publicly released because the informed consent (Ethics Approval No. KYLL-366, Institutional Review Board of Binzhou Medical University Hospital) did not authorize their public distribution; access to these restricted-use data is available from the Lead Contact upon reasonable request, subject to approval by the Institutional Review Board and execution of a data use agreement that protects participant privacy.
• All original code generated for this study, comprising the custom MATLAB scripts that implement the vector-autoregressive Granger causality analysis of the fNIRS time series, has been deposited at Zenodo together with the data and is publicly available at https://doi.org/10.5281/zenodo.20339370; the DOI is also listed in the Key Resources Table. All other analyses were performed using commercially available software (Vicon Nexus v2.15, MATLAB R2022b, and SPSS Statistics v27.0) following the procedures described in the STAR Method Details and Quantification and Statistical Analysis sections.
• Any additional information required to reanalyze the data reported in this paper is available from the lead contact upon request.

## Acknowledgments

We thank all patients who participated in this study. We also thank the staff of the gait laboratory at Binzhou Medical University Hospital for their technical assistance. This work was supported by the 10.13039/501100007129Shandong Provincial Natural Science Foundation (grant no. ZR2022MH063). The graphical abstract was created in BioRender. Sanders, A. (2026) https://BioRender.com/im93ijs.

## Author contributions

Conceptualization, L.Y. and W.L.; methodology, Z.S. and C.L.; investigation, B.D., B.H., M.G., and L.Z.; data curation, Z.S. and G.D.; formal analysis, G.D., J.S., and B.W.; writing – original draft, Z.S. and H.D.; writing – review and editing, L.Y., W.L., and P.M.; supervision, L.Y. and W.L.; funding acquisition, W.L. All authors approved the final version of the manuscript.

## Declaration of interests

The authors declare no competing interests.

## Declaration of generative AI and AI-assisted technologies in the writing process

During the preparation of this work, the authors used Claude (Anthropic) to improve the language, readability, and flow of the manuscript. All content was written by the authors; after using this tool, the authors reviewed and edited the result as needed and take full responsibility for the content of the publication.

## STAR★Methods

### Key resources table

**Table:** 

REAGENT or RESOURCE	SOURCE	IDENTIFIER
**Deposited data**		

De-identified processed gait, sEMG co-contraction, fNIRS HbO_2_, KOOS, and MTPA datasets	This paper, deposited at Zenodo	https://doi.org/10.5281/zenodo.20339370
Custom MATLAB scripts for vector-autoregressive Granger causality analysis of fNIRS time series	This paper, deposited at Zenodo	https://doi.org/10.5281/zenodo.20339370

**Software and algorithms**		

Vicon Nexus v2.15	Vicon Motion Systems Ltd.	https://www.vicon.com/software/nexus/
MATLAB R2022b	MathWorks Inc.	RRID: SCR_001622
SPSS Statistics v27.0	IBM Corp.	RRID: SCR_002865
G∗Power v3.1.9.7	Heinrich Heine University Düsseldorf	RRID: SCR_013726

**Other**		

8-camera Vicon Vero 2.2 motion capture system	Vicon Motion Systems Ltd.	Cat# Vero 2.2
AMTI OR6-7-400600 force platforms (×2)	Advanced Mechanical Technology Inc.	Cat# OR6-7-400600
NirSmart II-3000A 35-channel fNIRS system	Danyang Huichuang Medical Equipment Co., Ltd.	Cat# NirSmart II-3000A
Noraxon Ultium 10-channel wireless sEMG	Noraxon USA Inc.	Cat# Ultium EMG
Vicon Lock Lab synchronization unit	Vicon Motion Systems Ltd.	Cat# Lock Lab

### Experimental model and study participant details

This cross-sectional observational study was conducted at the gait laboratory of Binzhou Medical University Hospital. It was approved by the Institutional Review Board of Binzhou Medical University Hospital (Ethics Approval No. KYLL-366) and registered in the Chinese Clinical Trial Registry (ChiCTR2500101020). All procedures conformed to the principles of the Declaration of Helsinki and to the regulatory standards governing human-participant research in China. All participants provided written informed consent. Facial features in Figure 1B have been masked to protect identity; written informed consent for publication of the image was nonetheless obtained from the individual depicted. Investigators blinded to group allocation performed data collection and initial processing to reduce measurement bias.

All participants were of Han Chinese ethnicity and were recruited from the catchment area of Binzhou Medical University Hospital. The proportion of male participants did not differ between the higher- and lower-function groups (62.5% vs. 59.4%, *p* = 0.795; Table 1). Sex was therefore not included as a covariate in the primary between-group analyses; the available sample size did not support formal sex-stratified inferential testing, and exploratory sex-stratified inspection of the primary biomechanical outcomes did not reveal qualitative differences between male and female participants within either group. The reported age range (18–65 years) reflects skeletally mature adults with non-pathological bone quality, consistent with the target indication for the surgical procedure under investigation.

We calculated sample size *a priori* using G∗Power 3.1.9.7 to detect medium-to-large effects (Cohen’s *d* = 0.8) with α = 0.05 and 80% power, resulting in a minimum of 26 participants per group. The final enrollment of 32 participants per group ensured sufficient power for secondary analyses.

Between April 2025 and November 2025, we consecutively enrolled 64 patients evaluated 12 months after unilateral Schatzker Type II tibial plateau fracture surgery. We stratified participants by KOOS-ADL scores into higher-function (≥80, *n* = 32) and lower-function (≤70, *n* = 32) groups.^6^^,^^20^ These cutoffs reflect established reference values, where scores ≥80 indicate near-normal daily function (population mean 86.7) and scores ≤70 represent clinically meaningful impairment exceeding 15 points below healthy norms.^7^ We excluded patients with intermediate scores (71–79) to ensure clear group separation. Additionally, 23 healthy controls matched for age and sex were prospectively recruited during the same period and assessed using identical protocols. Pain intensity at the time of testing was assessed using a VAS, 0–10 to contextualize potential pain-related confounding.

Inclusion criteria were: unilateral Schatzker Type II fracture confirmed by preoperative computed tomography; age 18–65 years; body mass index 18.5–24.9 kg/m^2^; independent walking without assistive devices; and no neurological or musculoskeletal disorders affecting the contralateral limb. Exclusion criteria included multiple fractures or polytrauma; postoperative articular step-off >2 mm or lateral angulation >5°; prior lower-extremity surgery; neurological disease affecting gait; cardiovascular contraindications to testing; or inability to follow task instructions.

### Method details

#### Instrumentation

We synchronized three measurement systems with <10 μs precision using a Vicon Lock Lab synchronization unit (Figure 1A).^21^ We recorded three-dimensional gait kinematics and kinetics using an 8-camera Vicon motion analysis system (200 Hz) with embedded AMTI force platforms (1,000 Hz). Sixteen reflective markers were placed according to the Plug-in-Gait lower-body marker set (Figure 1B).

We measured muscle activation using a wireless 10-channel sEMG system (Noraxon Ultium, 2,000 Hz). Electrodes were placed bilaterally on the rectus femoris, semitendinosus, biceps femoris, tibialis anterior, and lateral gastrocnemius following SENIAM guidelines (Figure 1A).^22^ We normalized EMG signals using maximum voluntary contraction testing.

We assessed cortical hemodynamics using a 35-channel continuous-wave fNIRS system (NirSmart II-3000A, 11 Hz) with dual-wavelength near-infrared light (730 and 850 nm). Fourteen sources and fourteen detectors covered bilateral prefrontal, premotor, primary motor, and primary somatosensory cortices.^9^ A 30-mm source-detector separation yielded an estimated measurement depth of 15–20 mm (Figure 1A).

#### Experimental protocol

Participants performed three walking conditions in a randomized order using a Latin square design: (1) normal walking at a self-selected comfortable speed along an 8-m walkway; (2) dual-task walking combined with concurrent serial-3 subtraction, a validated paradigm to induce cognitive-motor interference^23^; and (3) balance-challenged walking along a 10-cm-wide tape-marked path, which required increased mediolateral stability control.

We implemented a block design optimized for fNIRS acquisition across all conditions, consisting of a 30-s pretask baseline, 15-s walking epochs, and 10-s rest intervals, with 6–8 blocks per condition and 60–120-s rest between conditions. All participants completed at least six valid trials for each walking condition.

#### Data processing

We processed motion data using Vicon Nexus 2.15 with the Plug-in-Gait model. We quantified limb symmetry using the symmetry index^24^:

$$SI=\frac{\left|X_{affected}-X_{unaffected}\right|}{0.5\times \left(X_{affected}+X_{unaffected}\right)}\times 100\%$$

We bandpass filtered EMG signals (50–250 Hz), rectified them, and normalized them to maximum voluntary contraction (MVC). We calculated muscle co-contraction using the CCI^25^:

$$CCI=\frac{2\times \min\left(EMG_{Extensor},EMG_{Flexor}\right)}{EMG_{Extensor}+EMG_{Flexor}}\times 100\%$$

We processed fNIRS data according to established consensus guidelines using the modified Beer–Lambert law.^26^^,^^27^ We evaluated directional cortical connectivity using Granger causality analysis.^28^ We assessed brain-muscle coupling with Spearman correlations between cortical oxygenation and muscle co-contraction indices. We performed Granger causality analysis on regional fNIRS time series using vector autoregressive models, selecting the optimal lag order based on the Akaike Information Criterion (range: 2–4). We tested statistical significance with F-tests (*p* < 0.05, Bonferroni-corrected) and classified connection strength as strong (GC > 0.20), moderate (0.15–0.20), or weak (<0.15).

### Quantification and statistical analysis

Continuous variables were tested for normality using the Shapiro–Wilk test (α = 0.05). Normally distributed variables are reported as mean ± SD and were compared between the lower- and higher-function groups using independent-samples t-tests; non-normally distributed variables are reported as median (Q1, Q3) and were compared using Mann–Whitney U tests. Categorical variables are reported as n (%) and were compared using Pearson χ^2^ or Fisher exact tests as appropriate.

Effect sizes are reported as Cohen’s d for parametric comparisons and as the rank-biserial correlation r for non-parametric comparisons, with the convention that |d| ≥ 0.8 and |r| ≥ 0.5 represent large effects. Family-wise error was controlled by Bonferroni correction within each domain: α = 0.0125 for the four primary biomechanical comparisons in normal and dual-task walking, α = 0.007 for the seven primary biomechanical comparisons in balance-challenged walking, α = 0.006 for the eight muscle co-contraction comparisons per condition, and α = 0.00143 for the 35-channel fNIRS comparisons within each walking condition. Exploratory brain-muscle coupling correlations are reported at uncorrected *p* < 0.05 with a note that none survived FDR correction (Benjamini–Hochberg, q < 0.05) across 140 channel × muscle pairs per condition; correlation method (Pearson r or Spearman ρ) was determined channel-by-channel by the Shapiro–Wilk test on the joint distribution.

Granger causality was estimated on regional fNIRS time series using vector-autoregressive models; the optimal lag order (range 2–4) was selected by the Akaike Information Criterion, and directional effects were tested with F-tests (Bonferroni-corrected α). Connection strength was categorized as strong (GC > 0.20), moderate (0.15 ≤ GC ≤ 0.20), or weak (GC < 0.15).

All analyses were performed in IBM SPSS Statistics v27.0 and MATLAB R2022b. Statistical significance was set at *p* < 0.05 (two-tailed). The number of biological units is *n* = 32 patients per group (64 in total) for all primary and secondary group-level comparisons unless otherwise specified. Per-figure and per-table statistical details, including test names, exact *p* values, available test statistics (t, U, F, r, or ρ), degrees of freedom where applicable, effect sizes with 95% confidence intervals, and the number of biological units underlying each comparison, are provided in Table S52.

#### Additional resources

Chinese Clinical Trial Registry registration: ChiCTR2500101020 (https://www.chictr.org.cn/showproj.html?proj=267647).
