# Supplementary material for: Central and peripheral neuromuscular mechanisms underlying functional recovery heterogeneity in tibial plateau fractures
Source: iScience. 2026 Jun 25;29(7):116490. doi: 10.1016/j.isci.2026.116490 (PMC13319370; doi:10.1016/j.isci.2026.116490)
Supplement: Document S1. Figures S1–S3 and Tables S1–S52 [file mmc1.pdf]

iScience, Volume *29*

Supplemental information

Central and peripheral neuromuscular mechanisms underlying functional recovery heterogeneity in tibial plateau fractures

Zihao Sun, Gangqiang Du, Chao Liu, Hongzhen Du, Jianxin Sun, Baoju Wang, Bixuan Duan, Binbin Huang, Meng Guo, Lina Zhang, Pei Ma, Li Yu, and Wei Li

Supplemental Figures

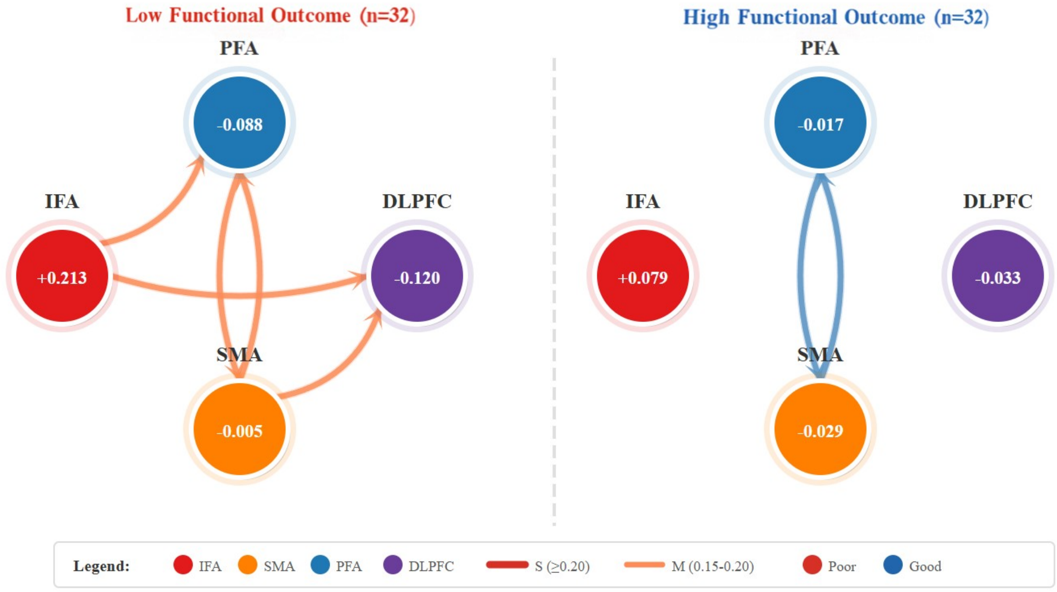

Supplementary Figure S1. Granger causality networks during normal-paced walking comparing the Lower Functional Outcome group (n=32, left) versus the Higher Functional Outcome group (n=32, right). The Lower Functional Outcome group demonstrates enhanced IFA driving (net flow: +0.213 vs. +0.079) and increased network connectivity (5 vs. 2 connections).

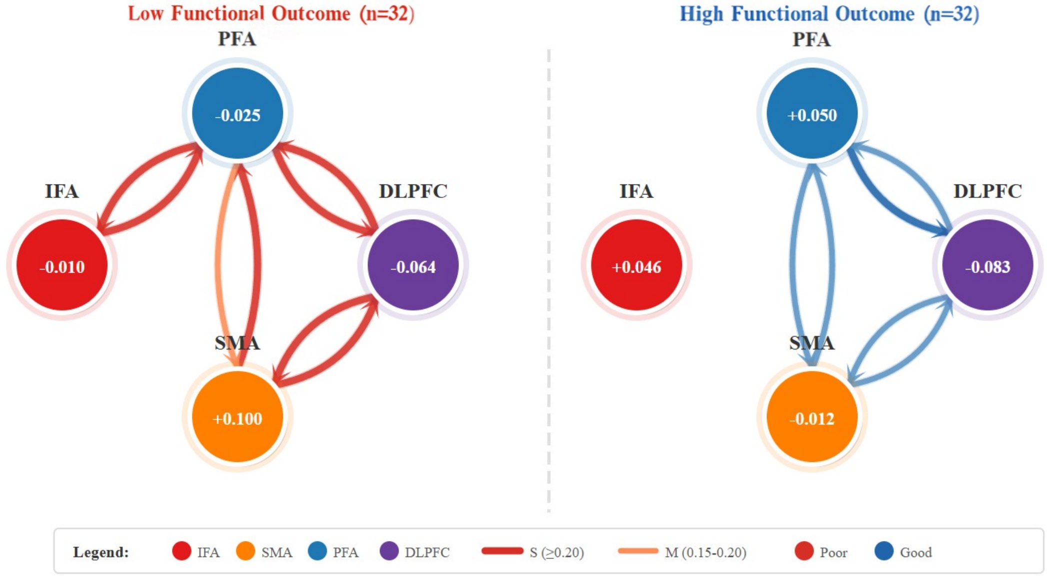

Supplementary Figure S2. Granger causality networks during dual-task walking. The Lower Functional Outcome group demonstrates SMA-dominated control with high network complexity (8 vs. 6 connections, 7 vs. 1 strong connections). Hierarchical ranking: Higher Functional Outcome = SMA (R1) → IFA (R2) → PFA (R3) → DLPFC (R4); Lower Functional Outcome = PFA (R1) → IFA (R2) → SMA (R3) → DLPFC(R4).

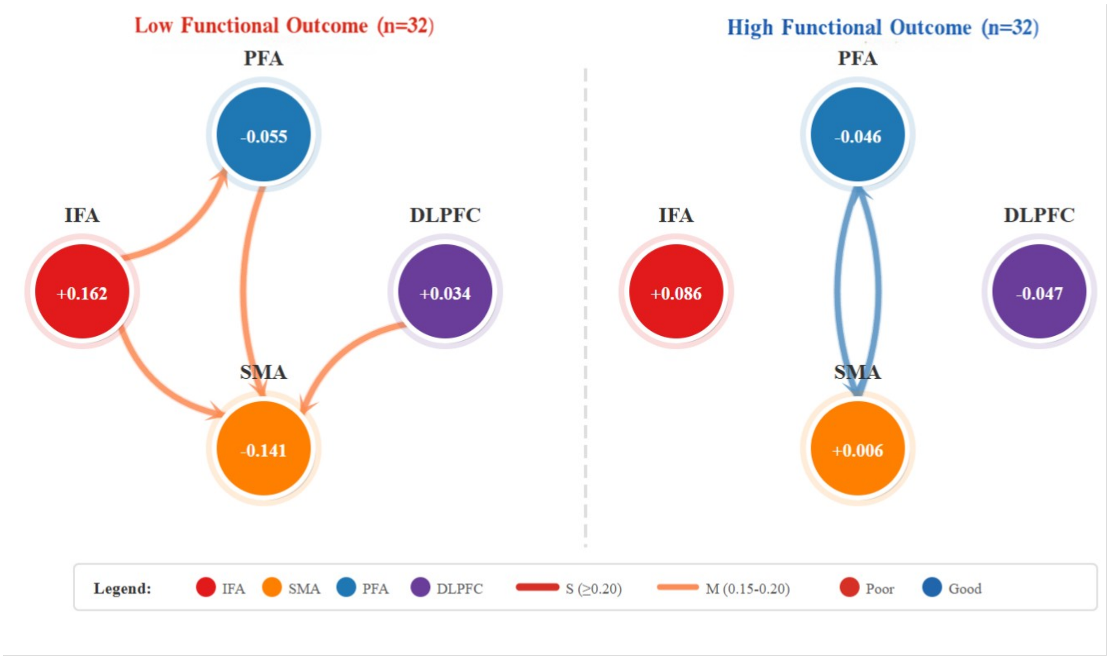

Supplementary Figure S3. Granger causality networks during balance-challenged walking. The Lower Functional Outcome group demonstrates IFA-dominated control with information converging on SMA (primary receiver, -0.141). Between-group difference: 4 vs. 2 connections.

Supplemental Tables

Supplementary Table S1

Comparison of Biomechanical Parameters Among Healthy Controls, Higher Functional Outcome, and Lower Functional Outcome Groups During Normal Walking

Parameter	Groups			Kruskal-Wallis *P*	Post-hoc *P*-Values			Effect Size (Cohen's d)		
	Healthy (n=23)	HFO (n=32)	LFO (n=32)		H vs HFO	H vs LFO	HFO vs LFO	H vs HFO	H vs LFO	HFO vs LFO
Pain Assessment										
VAS Score (0-10)	0.00 ± 0.00	0.31 ± 0.46	0.47 ± 0.61	**0.003****	**0.003****	**0.001****	0.357	0.882	1.002	0.288
Symmetry Indices										
Knee Flexion-Extension ROM SI (%)	5.04 ± 4.14	5.99 ± 3.40	18.67 ± 10.00	**<0.001*****	0.257	**<0.001*****	**<0.001*****	0.255	1.683	1.698
Hip Flexion-Extension ROM SI (%)	9.53 ± 7.24	9.58 ± 7.16	7.56 ± 5.88	0.509	0.871	0.389	0.286	0.007	0.304	0.308
Ankle ROM SI (%)	21.54 ± 14.83	20.43 ± 11.80	24.80 ± 22.87	0.992	0.939	0.966	0.909	0.084	0.164	0.241
Joint Range of Motion - Sagittal Plane										
Knee Flexion-Extension ROM (°) - Left	61.04 ± 6.17	57.91 ± 5.35	52.73 ± 7.88	**<0.001*****	**0.014***	**<0.001*****	0.052	0.549	1.151	0.770
Knee Flexion-Extension ROM (°) - Right	60.46 ± 4.19	58.42 ± 5.02	63.14 ± 5.55	**<0.001*****	**0.017***	**0.032***	**<0.001*****	0.435	0.532	0.891
Hip Flexion-Extension ROM (°) - Left	45.78 ± 3.26	42.65 ± 7.26	43.48 ± 2.91	**0.011***	**0.009****	**0.007****	0.763	0.528	0.752	0.150
Hip Flexion-Extension ROM (°) - Right	45.74 ± 4.90	40.78 ± 7.40	44.98 ± 3.90	**0.001****	**0.002****	0.500	**0.002****	0.765	0.175	0.710
Ankle ROM (°) - Left	26.79 ± 5.40	27.15 ± 5.76	28.56 ± 4.51	0.172	0.952	0.145	0.089	0.064	0.361	0.272
Ankle ROM (°) - Right	29.99 ± 6.06	31.49 ± 6.88	35.27 ± 15.20	0.455	0.479	0.264	0.432	0.228	0.431	0.321
Joint Range of Motion - Coronal Plane										
Knee Adduction-Abduction ROM (°) - Left	33.86 ± 14.68	20.74 ± 11.17	23.93 ± 10.46	**<0.001*****	**<0.001*****	**0.008****	0.073	1.030	0.802	0.295
Knee Adduction-Abduction ROM (°) - Right	24.46 ± 9.10	19.12 ± 8.05	19.39 ± 6.34	**0.035***	**0.014***	**0.045***	0.596	0.629	0.668	0.037
Hip Adduction-Abduction ROM (°) - Left	12.43 ± 2.95	12.61 ± 2.77	11.20 ± 2.47	0.081	0.627	0.229	**0.021***	0.066	0.458	0.539
Hip Adduction-Abduction ROM (°) - Right	10.77 ± 2.48	13.16 ± 3.15	11.11 ± 2.85	**0.003****	**0.003****	0.871	**0.005****	0.827	0.126	0.682

Note:

Data are presented as mean ± SD.

Symmetry Index (SI) = |Left - Right| / ((|Left| + |Right|)/2) × 100%

*P < 0.05; **P < 0.01; ***P < 0.001

Statistical analysis: Kruskal-Wallis test for overall comparison; Mann-Whitney U test for post-hoc pairwise comparisons.

Abbreviations:

H: Healthy controls; HFO: Higher Functional Outcome (KOOS-ADL ≥ 80); LFO: Lower Functional Outcome (KOOS-ADL ≤ 70); ROM: Range of motion; SI: Symmetry index; SD: Standard deviation

Supplementary Table S2

KOOS Activities of Daily Living (ADL) Subscale Comparison Between Higher Functional Outcome and Lower Functional Outcome Groups

KOOS-ADL Item	Higher Functional Outcome (n=32)		Lower Functional Outcome (n=32)		Test Method	P Value	Effect Size
	Mean ± SD	Median (IQR)	Mean ± SD	Median (IQR)			
Individual ADL Items (0-4 scale, lower = better)							
Descending stairs	0.31 ± 0.47	0.00 (0.00, 0.25)	2.72 ± 0.92	3.00 (2.00, 3.00)	Mann-Whitney U	**<0.001*** **	0.86[a]
Ascending stairs	0.55 ± 0.51	1.00 (0.00, 1.00)	1.38 ± 0.83	1.00 (1.00, 2.00)	Mann-Whitney U	**<0.001*** **	0.51[a]
Rising from sitting	0.55 ± 0.51	1.00 (0.00, 1.00)	1.38 ± 0.79	1.00 (1.00, 2.00)	Mann-Whitney U	**<0.001*** **	0.52[a]
Standing	0.62 ± 0.68	1.00 (0.00, 1.00)	1.16 ± 0.72	1.00 (1.00, 2.00)	Mann-Whitney U	**0.004** **	0.36[a]
Bending to floor	0.45 ± 0.51	0.00 (0.00, 1.00)	1.53 ± 0.88	1.50 (1.00, 2.00)	Mann-Whitney U	**<0.001*** **	0.58[a]
Walking on flat surface	0.66 ± 0.55	1.00 (0.00, 1.00)	1.59 ± 0.98	1.00 (1.00, 2.75)	Mann-Whitney U	**<0.001*** **	0.51[a]
Getting in/out of car	0.66 ± 0.48	1.00 (0.00, 1.00)	1.75 ± 0.72	2.00 (1.00, 2.00)	Mann-Whitney U	**<0.001*** **	0.69[a]
Going shopping	0.86 ± 0.58	1.00 (0.00, 1.00)	2.00 ± 0.95	2.00 (1.00, 3.00)	Mann-Whitney U	**<0.001*** **	0.59[a]
Putting on socks/stockings	0.93 ± 0.65	1.00 (0.00, 1.25)	1.31 ± 0.59	1.00 (1.00, 2.00)	Mann-Whitney U	0.149	0.18[a]
Rising from bed	0.66 ± 0.55	1.00 (0.00, 1.00)	1.19 ± 0.78	1.00 (1.00, 2.00)	Mann-Whitney U	**0.007** **	0.34[a]
Taking off socks/stockings	0.83 ± 0.71	1.00 (0.00, 1.00)	1.50 ± 0.72	1.50 (1.00, 2.00)	Mann-Whitney U	**<0.001*** **	0.43[a]
Lying in bed	0.79 ± 0.49	1.00 (0.75, 1.00)	1.59 ± 0.80	1.50 (1.00, 2.00)	Mann-Whitney U	**<0.001*** **	0.54[a]
Getting in/out of bath	0.62 ± 0.49	1.00 (0.00, 1.00)	2.00 ± 0.72	2.00 (2.00, 2.00)	Mann-Whitney U	**<0.001*** **	0.77[a]
Sitting	0.17 ± 0.38	0.00 (0.00, 0.00)	0.28 ± 0.58	0.00 (0.00, 0.00)	Mann-Whitney U	0.453	0.09[a]

KOOS-ADL Item	Higher Functional Outcome (n=32)		Lower Functional Outcome (n=32)		Test Method	P Value	Effect Size
	Mean ± SD	Median (IQR)	Mean ± SD	Median (IQR)			
Getting on/off toilet	0.97 ± 0.57	1.00 (1.00, 1.00)	1.66 ± 0.79	2.00 (1.00, 2.00)	Mann-Whitney U	**<0.001*****	0.49[a]
Heavy domestic duties	0.69 ± 0.54	1.00 (0.00, 1.00)	1.97 ± 0.82	2.00 (1.25, 3.00)	Mann-Whitney U	**<0.001*****	0.70[a]
Light domestic duties	0.59 ± 0.50	1.00 (0.00, 1.00)	0.84 ± 0.57	1.00 (0.00, 1.00)	Mann-Whitney U	0.061	0.23[a]
KOOS-ADL Total Score							
**KOOS-ADL Score (0-100, higher = better)**	**83.32 ± 2.47**	**83.09 (80.88, 85.29)**	**61.31 ± 5.09**	**61.76 (58.82, 64.71)**	**Mann-Whitney U**	**<0.001*****	**0.86[a]**

Note:

Data are presented as mean ± SD and median (Q1, Q3).

Bonferroni corrected α = 0.003 for 18 comparisons.

[a]Effect size reported as r (for Mann-Whitney U test): small ≥0.1, medium ≥0.3, large ≥0.5

*P < 0.05; **P < 0.01; ***P < 0.001

Statistical analysis: All variables showed non-normal distribution (Shapiro-Wilk test, p < 0.05 for at least one group); Mann-Whitney U test was applied for all comparisons.

Higher Functional Outcome: KOOS-ADL total score ≥80 (n=32); Lower Functional Outcome: KOOS total score ≤70 (n=32)

Individual ADL items scored from 0 (no difficulty) to 4 (extreme difficulty).

KOOS-ADL total score transformed to 0-100 scale, where 100 = no difficulty.

Abbreviations:

ADL: Activities of Daily Living; KOOS: Knee injury and Osteoarthritis Outcome Score; SD: Standard deviation; IQR: Interquartile range (Q1, Q3)

SI

Supplementary Table S3

Comparison of Joint Range of Motion Symmetry During Normal Speed Walking Between Lower Functional Outcome and Higher Functional Outcome Groups(percentage)

Variable	Group		*P*-value	EffectSize
	Lower Functional Outcome(n=32)	Higher Functional Outcome(n=32)		
Ankle Joint				
Coronal Plane	24.80 ± 22.87	20.43 ± 11.80	0.340	0.240[a]
Sagittal Plane	34.39 (11.39, 56.76)	17.71 (7.17, 36.01)	0.073	0.224[b]
Transverse Plane	18.39 (8.02, 30.07)	15.46 (7.35, 36.95)	0.799	0.032[b]
Hip Joint				
Coronal Plane	6.62 (2.42, 11.74)	7.80 (3.49, 13.12)	0.283	0.134[b]
Sagittal Plane	13.48 (8.16, 19.10)	9.03 (5.23, 20.71)	0.308	0.128[b]

Transverse Plane	17.88 (7.58, 26.78)	18.55 (4.11, 50.99)	0.629	0.061[b]
Knee Joint				
Coronal Plane	14.12 (11.47, 25.54)	6.16 (2.67, 8.37)	**<0.001*****	0.755[b]
Sagittal Plane	31.74 (21.60, 58.85)	30.72 (17.98, 43.33)	0.428	0.099[b]
Transverse Plane	18.34 (11.28, 35.76)	40.83 (25.08, 56.83)	0.010**	0.322[b]

Note:

Data presented as median (Q1, Q3) unless otherwise noted.

Bonferroni corrected α = 0.006 for 9 comparisons.

***P < 0.001; **P < 0.01; *P < 0.05

[a]Effect size reported as Cohen's d; [b]Effect size reported as r value

Abbreviations:

ROM: Range of Motion

Supplementary Table S4

Comparison of Peak Joint Moment Symmetry During Normal Speed Walking Between Lower Functional Outcome and Higher Functional Outcome Groups(percentage)

Variable	Group		*P*-value	EffectSize
	Lower Functional Outcome(n=32)	Higher Functional Outcome(n=32)		
Ankle Joint				
Coronal Plane	14.06 (5.44, 22.25)	11.24 (4.30, 19.30)	0.428	0.099[b]
Sagittal Plane	38.17 (2.39, 114.88)	12.06 (-75.59, 45.86)	0.040*	0.257[b]
Transverse Plane	17.69 (6.84, 42.26)	17.97 (6.23, 31.92)	0.601	0.066[b]
Hip Joint				
Coronal Plane	-17.24 (-30.24, 1.87)	-13.53 (-30.84, -5.52)	0.883	0.018[b]
Sagittal Plane	9.74 (0.30, 32.94)	37.71 (8.83, 53.98)	0.013*	0.312[b]
Transverse Plane	34.67 (16.08, 82.68)	20.45 (8.96, 46.61)	0.029*	0.273[b]
Knee Joint				
Coronal Plane	15.29 ± 86.82	1.42 ± 33.05	0.401	0.211[a]
Sagittal Plane	32.64 (14.04, 80.20)	21.03 (9.15, 39.61)	0.066	0.230[b]
Transverse Plane	27.89 (7.01, 42.48)	16.21 (5.71, 28.22)	0.188	0.165[b]

Note:

Data presented as median (Q1, Q3) unless otherwise noted.

Bonferroni corrected α = 0.006 for 9 comparisons.

***P < 0.001; **P < 0.01; *P < 0.05

[a]Effect size reported as Cohen's d; [b]Effect size reported as r value

Supplementary Table S5

Comparison of Joint Range of Motion Symmetry During Dual Task Between Lower Functional Outcome and Higher Functional Outcome Groups

Variable	Group		*P*-value	EffectSize
	**Lower Functional Outcome(n=32)**	**Higher Functional Outcome(n=32)**		
Ankle Joint				
Coronal Plane	13.08 ± 9.26	12.24 ± 7.96	0.698	0.097[a]
Sagittal Plane	14.89 (4.73, 38.67)	15.82 (9.11, 31.44)	0.979	0.003[b]
Transverse Plane	19.02 ± 12.58	22.62 ± 13.99	0.283	0.271[a]
Hip Joint				
Coronal Plane	7.86 ± 4.47	8.80 ± 5.54	0.456	0.188[a]
Sagittal Plane	14.59 (6.20, 30.04)	9.11 (4.29, 19.09)	0.076	0.222[b]
Transverse Plane	15.61 (5.32, 27.59)	23.09 (12.15, 40.57)	0.058	0.237[b]
Knee Joint				
Coronal Plane	32.83 ± 20.74	7.77 ± 4.83	**<0.001*** **	1.664[a]
Sagittal Plane	41.98 (24.36, 68.98)	44.43 (33.30, 76.65)	0.081	0.218[b]
Transverse Plane	15.99 (7.77, 32.54)	26.49 (16.98, 43.73)	0.028*	0.275[b]

Note:

Data presented as median (Q1, Q3) unless otherwise noted.

Bonferroni corrected α = 0.006 for 9 comparisons.

***P < 0.001; **P < 0.01; *P < 0.05

[a]Effect size reported as Cohen's d; [b]Effect size reported as r value

Abbreviations:

ROM: Range of Motion

Supplementary Table S6

Comparison of Peak Joint Moment Symmetry During Dual Task Between Lower Functional Outcome and Higher Functional Outcome Groups

Variable	Group		*P*-value	EffectSize
	**Lower Functional Outcome(n=32)**	**Higher Functional Outcome(n=32)**		
Ankle Joint				
Coronal Plane	17.73 (10.55, 33.45)	15.69 (5.17, 25.38)	0.151	0.180[b]
Sagittal Plane	92.97 (29.29, 136.07)	53.95 (-124.08, 95.62)	0.129	0.190[b]
Transverse Plane	20.44 (7.01, 57.03)	25.85 (5.83, 43.40)	0.485	0.087[b]
Hip Joint				
Coronal Plane	-8.30 (-69.09, 45.07)	-15.26 (-45.47, -6.82)	0.347	0.118[b]

Sagittal Plane	39.98 (12.83, 69.75)	33.93 (7.04, 61.00)	0.420	0.101[b]
Transverse Plane	33.64 (2.28, 53.31)	14.25 (-17.64, 34.32)	0.334	0.121[b]
Knee Joint				
Coronal Plane	-42.50 ± 57.92	7.25 ± 48.70	**<0.001*****	0.930[a]
Sagittal Plane	46.94 (23.17, 89.49)	31.56 (12.05, 62.31)	0.136	0.186[b]
Transverse Plane	32.42 (7.90, 60.69)	20.47 (3.48, 40.89)	0.237	0.148[b]

Note:

Data presented as median (Q1, Q3) unless otherwise noted.

Bonferroni corrected α = 0.006 for 9 comparisons.

***P < 0.001; **P < 0.01; *P < 0.05

[a]Effect size reported as Cohen's d; [b]Effect size reported as r value

Supplementary Table S7

Comparison of Joint Range of Motion Symmetry During Balance Task Between Lower Functional Outcome and Higher Functional Outcome Groups

Variable	Group		*P*-value	EffectSize
	Lower Functional Outcome(n=32)	Higher Functional Outcome(n=32)		
Ankle Joint				
Coronal Plane	20.15 (10.80, 32.97)	11.29 (3.94, 17.58)	0.003**	0.373[b]
Sagittal Plane	32.13 ± 22.55	18.84 ± 13.26	0.006**	0.718[a]
Transverse Plane	24.81 ± 16.62	24.78 ± 18.02	0.994	0.002[a]
Hip Joint				
Coronal Plane	7.01 (4.31, 11.07)	7.03 (3.46, 10.89)	0.952	0.008[b]
Sagittal Plane	23.03 (5.97, 37.57)	14.18 (5.64, 23.25)	0.072	0.225[b]
Transverse Plane	26.89 (8.88, 51.54)	26.84 (7.94, 69.62)	0.555	0.074[b]
Knee Joint				
Coronal Plane	32.74 ± 13.09	7.29 ± 5.42	**<0.001*****	2.541[a]
Sagittal Plane	52.68 ± 36.96	51.50 ± 18.08	0.872	0.040[a]
Transverse Plane	21.03 ± 14.65	25.12 ± 18.76	0.335	0.243[a]

Note:

Data presented as median (Q1, Q3) unless otherwise noted.

Bonferroni corrected α = 0.006 for 9 comparisons.

***P < 0.001; **P < 0.01; *P < 0.05

[a]Effect size reported as Cohen's d; [b]Effect size reported as r value

Abbreviations:

ROM: Range of Motion

Supplementary Table S8

Comparison of Peak Joint Moment Symmetry During Balance Task Between Lower Functional Outcome and Higher Functional Outcome Groups

Variable	Group		*P*-value	EffectSize
	Lower Functional Outcome(n=32)	Higher Functional Outcome(n=32)		
Ankle Joint				
Coronal Plane	26.35 (7.45, 33.11)	10.43 (5.88, 16.06)	0.008**	0.329[b]
Sagittal Plane	60.88 (19.39, 85.36)	47.16 (-11.80, 113.49)	0.883	0.019[b]
Transverse Plane	42.46 (9.56, 61.77)	15.26 (7.87, 28.36)	0.005**	0.354[b]
Hip Joint				
Coronal Plane	-19.13 (-65.71, 62.29)	-6.05 (-15.21, 13.04)	0.138	0.186[b]
Sagittal Plane	41.75 ± 62.03	44.28 ± 30.96	0.837	0.052[a]
Transverse Plane	56.61 (16.77, 84.07)	12.52 (5.37, 23.69)	**<0.001*** **	0.529[b]
Knee Joint				
Coronal Plane	13.93 ± 90.65	4.36 ± 43.09	0.592	0.135[a]
Sagittal Plane	44.16 (8.30, 85.69)	30.26 (13.80, 52.50)	0.301	0.129[b]
Transverse Plane	23.49 (10.27, 57.93)	21.07 (9.89, 36.41)	0.354	0.116[b]

Note:

Data presented as median (Q1, Q3) unless otherwise noted.

Bonferroni corrected α = 0.006 for 9 comparisons.

***P < 0.001; **P < 0.01; *P < 0.05

[a]Effect size reported as Cohen's d; [b]Effect size reported as r value

Supplementary Table S9

Between-Group Comparison: Spatiotemporal Parameters During Different Walking Tasks

Variable	Normal Walking				Dual Task				Balance Task			
	**Lower Functional Outcome (n=32)**	**Higher Functional Outcome (n=32)**	**P-value**	**Effect Size**	**Lower Functional Outcome (n=32)**	**Higher Functional Outcome (n=32)**	**P-value**	**Effect Size**	**Lower Functional Outcome (n=32)**	**Higher Functional Outcome (n=32)**	**P-value**	**Effect Size**
Affected Side												
Cadence	98.47 ± 9.10	97.44 ± 12.62	0.71	0.093[a]	99.30 (86.86, 104.50)	100.46 (86.37, 103.78)	0.97	0.005[b]	95.71 ± 10.81	106.77 ± 10.77	**<0.001*** **	1.025[a]
Step Length	0.64 (0.63, 0.65)	0.65 (0.61, 0.66)	0.59	0.067[b]	0.62 (0.60, 0.67)	0.64 (0.62, 0.66)	0.31	0.126[b]	0.62 ± 0.03	0.65 ± 0.05	0.017*	0.615[a]
Step Width	0.14 (0.11, 0.20)	0.19 (0.12, 0.22)	0.14	0.186[b]	0.13 ± 0.05	0.12 ± 0.06	0.35	0.236[a]	0.14 (0.11, 0.21)	0.07 (0.04, 0.11)	**<0.001*** **	0.523[b]
Stride Length	1.25 ±	1.24 ±	0.50	0.17	1.23	1.26	0.10	0.20	1.23 ±	1.30 ±	**<0.001***	1.227[a]

Variable	Normal Walking				Dual Task				Balance Task			
	**Lower Functional Outcome (n=32)**	**Higher Functional Outcome (n=32)**	**P-value**	**Effect Size**	**Lower Functional Outcome (n=32)**	**Higher Functional Outcome (n=32)**	**P-value**	**Effect Size**	**Lower Functional Outcome (n=32)**	**Higher Functional Outcome (n=32)**	**P-value**	**Effect Size**
	0.06	0.07		0[a]	(1.17, 1.31)	(1.23, 1.32)		6[b]	0.06	0.06	**	
Walking Speed	1.03 ± 0.12	1.01 ± 0.16	0.62	0.124[a]	1.01 ± 0.15	1.01 ± 0.13	0.92	0.027[a]	0.92 (0.89, 1.06)	1.20 (1.10, 1.27)	**<0.001***	0.591[b]
Unaffected Side												
Cadence	102.68 (90.50, 106.29)	101.18 (91.05, 107.41)	0.68	0.052[b]	103.43 (96.13, 111.89)	98.68 (91.94, 106.72)	0.08	0.218[b]	89.52 (84.45, 101.82)	109.49 (97.13, 111.45)	**0.003****	0.366[b]
Step Length	0.62 ± 0.04	0.63 ± 0.05	0.82	0.056[a]	0.63 ± 0.04	0.63 ± 0.05	0.53	0.160[a]	0.63 ± 0.04	0.65 ± 0.02	0.006**	0.717[a]
Step Width	0.15 (0.11, 0.20)	0.15 (0.11, 0.22)	0.84	0.025[b]	0.13 ± 0.03	0.13 ± 0.06	0.77	0.074[a]	0.14 (0.10, 0.17)	0.07 (0.02, 0.20)	0.30	0.129[b]
Stride Length	1.25 ± 0.06	1.26 ± 0.07	0.85	0.048[a]	1.26 ± 0.07	1.26 ± 0.07	0.93	0.022[a]	1.26 ± 0.05	1.30 ± 0.05	**0.005****	0.722[a]
Walking Speed	1.05 (0.94, 1.14)	1.08 (0.95, 1.14)	0.71	0.047[b]	1.09 (1.01, 1.18)	1.04 (0.94, 1.13)	0.08	0.220[b]	0.93 (0.87, 1.07)	1.20 (1.07, 1.23)	**0.001****	0.398[b]

Note:

Data are presented as mean ± SD or median (Q1, Q3).

Bonferroni corrected α = 0.005 for 10 comparisons per condition

[a]Effect size reported as Cohen's d; [b]Effect size reported as r value

*P < 0.05; **P < 0.01; ***P < 0.001

Statistical analysis: Independent t-test for normally distributed data; Mann-Whitney U test for non-normally distributed data

Normality was assessed using the Shapiro-Wilk test (P > 0.05 indicates normal distribution)

Supplementary Table S10

Between-Group Comparison: Foot Progression Angles During Different Walking Tasks

**Variable**	**Normal Walking**				**Dual Task**				**Balance Task**			
	**Lower Functional Outcome (n=32)**	**Higher Functional Outcome (n=32)**	**P-value**	**Effect Size**	**Lower Functional Outcome (n=32)**	**Higher Functional Outcome (n=32)**	**P-value**	**Effect Size**	**Lower Functional Outcome (n=32)**	**Higher Functional Outcome (n=32)**	**P-value**	**Effect Size**
Affected Side												
FPA-X Max	-21.16 (-28.46, -15.23)	-27.90 (-31.28, -23.81)	0.009**	0.324[b]	-22.11 (-30.36, -13.50)	-23.84 (-32.17, -17.56)	0.50	0.085[b]	-24.70 (-30.50, -19.51)	-27.46 (-31.63, -22.40)	0.16	0.178[b]
FPA-X Min	-62.42 ± 11.21	-63.36 ± 13.97	0.76	0.076[a]	-60.95 (-72.04, -51.57)	-62.10 (-70.40, -56.84)	0.57	0.072[b]	-66.71 ± 10.89	-63.87 ± 10.36	0.28	0.270[a]
FPA-X ROM	40.16 ± 9.02	35.18 ± 11.53	0.06	0.485[a]	37.89 (34.20, 44.79)	38.55 (34.48, 43.15)	0.85	0.023[b]	41.91 ± 7.59	36.40 ± 8.71	0.006 **	0.684[a]
FPA-Y Max	5.33 ± 5.95	4.39 ± 6.16	0.52	0.155[a]	7.46 (3.08, 13.24)	4.05 (-0.21, 8.75)	0.07	0.226[b]	6.51 ± 6.13	6.09 ± 5.83	0.77	0.072[a]
FPA-Y Min	-4.20 (-8.23, -2.24)	-3.47 (-7.20, -0.87)	0.48	0.087[b]	-4.53 ± 3.80	-4.81 ± 6.85	0.84	0.051[a]	-4.27 (-8.01, -1.66)	-2.33 (-6.55, 1.51)	0.16	0.177[b]
FPA-Y ROM	9.66	7.86 (5.99,	0.15	0.180[b]	12.10 (8.36, 17.11)	9.40 (7.20,	0.07	0.227[b]	10.80	8.42 (6.33,	0.05	0.243[b]

Variable	Normal Walking				Dual Task				Balance Task			
	Lower Functional Outcome (n=32)	Higher Functional Outcome (n=32)	P-value	Effect Size	Lower Functional Outcome (n=32)	Higher Functional Outcome (n=32)	P-value	Effect Size	Lower Functional Outcome (n=32)	Higher Functional Outcome (n=32)	P-value	Effect Size
	(7.54, 13.31)	10.87)				12.20)			(8.11, 14.57)	12.62)		
FPA-Z Max	1.76 (-9.23, 13.31)	0.24 (-8.09, 10.56)	0.77	0.036[b]	3.06 (-7.96, 12.78)	-1.25 (-6.18, 10.46)	0.40	0.105[b]	3.81 (-6.85, 12.93)	2.01 (-5.48, 9.90)	0.67	0.054[b]
FPA-Z Min	-4.71 (-15.30, 7.16)	-3.99 (-12.65, 8.51)	0.84	0.025[b]	-6.37 (-17.88, 7.42)	-8.39 (-16.56, 6.27)	0.57	0.072[b]	-8.37 ± 10.67	-4.47 ± 8.43	0.11	0.407[a]
FPA-Z ROM	8.24 (6.25, 12.95)	5.46 (4.70, 7.33)	0.028*	0.275[b]	9.96 (6.33, 16.31)	8.17 (5.48, 11.16)	0.23	0.151[b]	12.18 (6.70, 18.77)	6.48 (4.24, 9.55)	**0.002 ****	0.390[b]
Unaffected Side												
FPA-X Max	-21.76 (-32.06, -15.89)	-29.68 (-31.80, -24.85)	0.031*	0.270[b]	-24.59 (-33.71, -16.82)	-23.23 (-30.89, -16.28)	0.77	0.037[b]	-25.53 (-32.53, -17.79)	-28.53 (-32.70, -24.36)	0.22	0.154[b]
FPA-X Min	-65.68 ± 14.66	-66.73 ± 11.25	0.75	0.080[a]	-66.04 ± 12.88	-64.00 ± 10.57	0.49	0.175[a]	-68.73 ± 9.22	-66.01 ± 10.72	0.28	0.275[a]
FPA-X ROM	43.91 ± 11.94	36.83 ± 9.76	0.009**	0.660[a]	41.03 (35.45, 48.05)	40.77 (34.60, 46.80)	0.83	0.028[b]	43.21 ± 6.84	37.47 ± 8.26	**0.003 ****	0.776[a]
FPA-Y Max	5.07 ± 7.67	2.67 ± 7.73	0.20	0.313[a]	7.01 ± 7.73	4.90 ± 6.34	0.23	0.299[a]	6.13 ± 6.60	4.28 ± 7.56	0.30	0.264[a]
FPA-Y Min	-2.89 ± 6.03	-4.15 ± 5.39	0.38	0.223[a]	-4.77 ± 5.30	-3.89 ± 6.38	0.55	0.152[a]	-3.33 ± 5.36	-4.35 ± 6.16	0.48	0.178[a]
FPA-Y ROM	7.97 (6.40, 11.65)	7.35 (4.69, 9.84)	0.33	0.123[b]	11.77 (7.83, 16.72)	8.79 (6.03, 11.96)	0.06	0.233[b]	9.46 (6.82, 14.21)	8.63 (6.07, 12.15)	0.43	0.098[b]
FPA-Z Max	2.00 (-6.89, 11.75)	-2.54 (-10.64, 8.95)	0.41	0.103[b]	4.62 (-3.66, 11.15)	-1.16 (-7.92, 9.59)	0.13	0.189[b]	3.63 (-5.93, 13.71)	1.53 (-5.81, 9.88)	0.60	0.065[b]
FPA-Z Min	-9.38 (-19.09, 4.73)	-8.90 (-14.63, 5.31)	0.97	0.005[b]	-12.15 ± 10.06	-10.89 ± 9.03	0.60	0.131[a]	-11.20 ± 10.32	-7.85 ± 8.37	0.15	0.364[a]
FPA-Z ROM	9.79 (5.33, 14.71)	6.56 (4.73, 8.53)	0.06	0.238[b]	15.82 (10.41, 22.28)	10.13 (6.50, 14.72)	**0.039***	0.259[b]	14.83 (8.80, 20.89)	9.38 (6.90, 12.15)	0.007 **	0.338[b]

Note:

Data are presented as mean ± SD or median (Q1, Q3).

Bonferroni corrected α = 0.003 for 18 comparisons per condition

[a]Effect size reported as Cohen's d; [b]Effect size reported as r value

*P < 0.05; **P < 0.01; ***P < 0.001

Statistical analysis: Independent t-test for normally distributed data; Mann-Whitney U test for non-normally distributed data

Normality was assessed using the Shapiro-Wilk test (P > 0.05 indicates normal distribution)

Abbreviations:

FPA: Foot Progression Angle; Max: Maximum; Min: Minimum; ROM: Range of Motion

Supplementary Table S11

Between-Group Comparison: Pelvis Tilt Angles During Different Walking Tasks

Note:

Variable	Normal Walking				Dual Task				Balance Task			
	Lower Functional Outcome (n=32)	Higher Functional Outcome (n=32)	P-value	Effect Size	Lower Functional Outcome (n=32)	Higher Functional Outcome (n=32)	P-value	Effect Size	Lower Functional Outcome (n=32)	Higher Functional Outcome (n=32)	P-value	Effect Size
Affected Side												
PTA-X Max	4.10 (-5.30, 8.74)	0.05 (-9.77, 9.35)	0.27	0.138[b]	1.79 (-7.67, 9.36)	4.60 (-3.42, 12.61)	0.27	0.138[b]	0.92 (-8.45, 8.31)	0.78 (-1.40, 4.27)	0.91	0.014[b]
PTA-X Min	-0.24 (-11.13, 3.71)	-4.44 (-15.41, 5.09)	0.44	0.096[b]	-1.66 (-11.06, 1.23)	-0.17 (-7.50, 3.31)	0.24	0.148[b]	-3.97 (-13.58, 1.33)	-1.73 (-2.85, -0.91)	0.33	0.121[b]
PTA-X ROM	4.81 (3.83, 5.84)	4.35 (3.73, 5.36)	0.44	0.097[b]	3.79 (2.80, 4.28)	7.39 (5.38, 10.79)	<0.001***	0.710[b]	4.87 (3.57, 5.48)	3.39 (2.75, 5.12)	0.025*	0.280[b]
PTA-Y Max	2.30 ± 2.68	2.85 ± 2.59	0.41	0.210[a]	4.65 (2.84, 6.28)	2.56 (0.68, 4.02)	0.019*	0.294[b]	2.12 ± 2.14	4.78 ± 2.34	<0.001***	1.190[a]
PTA-Y Min	-3.80 (-6.07, -1.33)	-4.60 (-5.75, -2.84)	0.88	0.018[b]	-2.19 (-3.41, -1.57)	-4.52 (-8.43, -3.39)	<0.001***	0.457[b]	-3.95 ± 2.33	-1.67 ± 2.30	<0.001***	0.985[a]
PTA-Y ROM	6.10 ± 2.01	6.90 ± 1.34	0.07	0.465[a]	6.87 ± 1.97	7.85 ± 1.93	0.049*	0.502[a]	5.35 (4.26, 7.31)	6.42 (5.56, 7.18)	0.12	0.195[b]
PTA-Z Max	7.20 ± 5.63	5.47 ± 4.08	0.17	0.351[a]	5.53 (2.39, 8.84)	6.27 (5.16, 9.26)	0.23	0.151[b]	10.23 ± 4.43	5.31 ± 3.45	<0.001***	1.237[a]
PTA-Z Min	-5.46 (-8.62, -2.67)	-5.16 (-8.20, -2.80)	0.94	0.010[b]	-6.21 ± 2.96	-12.17 ± 4.35	<0.001***	1.600[a]	-8.87 (-11.40, -4.78)	-6.48 (-8.24, -4.89)	0.16	0.176[b]
PTA-Z ROM	12.85 (9.88, 15.53)	10.02 (8.31, 11.87)	0.06	0.233[b]	12.27 ± 6.20	19.54 ± 7.55	<0.001***	1.052[a]	17.61 ± 3.64	12.20 ± 3.81	<0.001***	1.452[a]
Unaffected Side												
PTA-X Max	3.91 (-9.34, 8.82)	0.00 (-11.33, 8.56)	0.29	0.131[b]	1.58 (-6.74, 4.91)	5.60 (1.29, 10.70)	0.005**	0.351[b]	1.73 (-9.05, 5.53)	1.20 (0.24, 3.41)	0.77	0.037[b]
PTA-X Min	0.58 (-13.75, 3.75)	-3.77 (-16.06, 5.44)	0.55	0.075[b]	-1.99 (-11.77, 1.36)	0.26 (-7.32, 3.54)	0.23	0.151[b]	-5.06 (-13.73, 1.17)	-1.88 (-2.70, -1.20)	0.26	0.141[b]
PTA-X ROM	4.22 (3.25, 5.83)	3.96 (3.31, 4.75)	0.41	0.102[b]	3.93 (3.27, 4.57)	6.41 (5.57, 9.24)	<0.001***	0.737[b]	4.59 (3.63, 6.21)	3.29 (2.77, 4.57)	0.004**	0.357[b]
PTA-Y Max	3.64 (1.39, 6.00)	4.66 (3.13, 5.70)	0.66	0.055[b]	2.22 (1.31, 3.46)	4.10 (2.90, 6.86)	0.001**	0.433[b]	4.19 ± 2.12	1.37 ± 2.55	<0.001***	1.203[a]
PTA-Y Min	-2.19 ± 2.71	-2.45 ± 2.91	0.72	0.091[a]	-3.69 (-5.86, -2.12)	-2.67 (-4.07, 0.38)	0.044*	0.252[b]	-1.92 ± 1.82	-4.92 ± 2.41	<0.001***	1.406[a]
PTA-Y ROM	5.96 ± 1.99	6.50 ± 1.68	0.25	0.293[a]	5.85 (4.81, 7.75)	6.61 (6.25, 7.61)	0.09	0.211[b]	5.53 (4.27, 7.85)	6.27 (5.17, 7.26)	0.22	0.153[b]
PTA-Z Max	6.15 ± 4.58	7.15 ± 3.62	0.34	0.242[a]	7.30 (5.18, 8.94)	12.35 (9.40, 14.67)	<0.001***	0.562[b]	9.35 (4.60, 11.40)	6.79 (5.30, 8.17)	0.12	0.195[b]
PTA-Z Min	-6.16 ± 5.08	-5.13 ± 3.18	0.33	0.243[a]	-4.43 (-11.46, -2.48)	-5.96 (-7.36, -5.14)	0.73	0.044[b]	-9.74 ± 3.15	-5.24 ± 3.49	<0.001***	1.352[a]
PTA-Z ROM	12.43 (10.31, 14.11)	11.67 (9.65, 13.62)	0.41	0.102[b]	12.51 (7.42, 18.74)	19.09 (13.75, 22.15)	0.003**	0.373[b]	17.24 (13.37, 19.82)	12.02 (10.12, 13.49)	<0.001***	0.547[b]

Data are presented as mean ± SD or median (Q1, Q3).

Bonferroni corrected α = 0.003 for 18 comparisons per condition

[a]Effect size reported as Cohen's d; [b]Effect size reported as r value

*P < 0.05; **P < 0.01; ***P < 0.001

Statistical analysis: Independent t-test for normally distributed data; Mann-Whitney U test for non-normally

distributed data

Normality was assessed using the Shapiro-Wilk test (P > 0.05 indicates normal distribution)

Abbreviations:

PTA: Pelvis Tilt Angle; Max: Maximum; Min: Minimum; ROM: Range of Motion

Supplementary Table S12

Comparison of Joint Extension/Abduction/External Rotation Angles During Dual-Task Walking Between Lower Functional Outcome and Higher Functional Outcome Groups

Variable	Group		*P*-value	EffectSize
	**Lower Functional Outcome(n=32)**	**Higher Functional Outcome(n=32)**		
Affected/Non-dominant Side				
APF (°)	-15.35 (-19.35, -11.43)	-14.46 (-19.56, -9.88)	0.601	0.07[b]
AAB (°)	-1.38 (-3.86, 1.77)	-2.56 (-5.13, -0.18)	0.011*	0.32[b]
AER (°)	-30.00 (-40.71, -20.22)	-23.27 (-32.19, -16.56)	0.003**	0.37[b]
HE (°)	-22.81 (-29.08, -17.25)	-24.02 (-27.99, -21.43)	0.259	0.14[b]
HAB (°)	-7.66 (-10.98, -4.47)	-4.78 (-7.84, -2.69)	0.043*	0.25[b]
HER (°)	3.51 (-7.28, 9.51)	-12.23 (-24.13, 0.47)	0.002**	0.39[b]
KE (°)	-4.21 (-6.74, -1.90)	-5.13 (-6.47, -3.77)	0.629	0.06[b]
KAB (°)	-1.95 (-4.24, 1.35)	-4.57 (-9.60, 1.07)	0.677	0.05[b]
KER (°)	-19.71 (-24.52, -15.01)	-10.73 (-15.58, -7.67)	**<0.001*** **	0.52[b]
UnAffected/Dominant Side				
APF (°)	-12.13 (-15.58, -9.78)	-12.48 (-17.77, -9.18)	0.477	0.09[b]
AAB (°)	-3.35 (-7.78, -0.52)	-4.14 (-8.03, -0.63)	0.717	0.05[b]
AER (°)	-24.04 (-27.39, -18.31)	-20.68 (-25.87, 2.75)	0.193	0.16[b]
HE (°)	-17.36 (-30.02, -13.11)	-23.27 (-26.21, -19.87)	0.143	0.18[b]
HAB (°)	-3.93 (-9.82, -0.46)	-5.85 (-9.13, -4.43)	0.136	0.19[b]
HER (°)	-19.51 (-22.38, -12.62)	-4.96 (-37.99, 15.89)	0.006**	0.34[b]
KE (°)	-4.35 (-6.96, -1.65)	-5.26 (-7.14, -1.11)	0.601	0.07[b]
KAB (°)	-3.52 (-4.19, -2.40)	-4.82 (-21.00, -0.18)	0.197	0.16[b]
KER (°)	-9.59 (-18.11, -6.84)	-23.21 (-25.92, -9.40)	0.048*	0.25[b]

Note:

Data presented as median (Q1, Q3).

Bonferroni corrected α = 0.003 for 18 comparisons

***P < 0.001; **P < 0.01; *P < 0.05

°: degrees

Negative values indicate motion opposite to the corresponding positive direction (extension vs. flexion, abduction vs. adduction, external vs. internal rotation)

[a]Effect size reported as Cohen's d; [b]Effect size reported as r value

Abbreviations:

APF: Ankle plantarflexion; AAB: Ankle abduction; AER: Ankle external rotation;

HE: Hip extension; HAB: Hip abduction; HER: Hip external rotation;

KE: Knee extension; KAB: Knee abduction; KER: Knee external rotation

Supplementary Table S13

Comparison of Joint Range of Motion During Dual-Task Walking Between Lower Functional Outcome and Higher Functional Outcome Groups

Variable	Group		*P*-value	EffectSize
	Lower Functional Outcome(n=32)	Higher Functional Outcome(n=32)		
Affected/Non-dominant Side				
A F-E ROM (°)	27.38 (23.31, 31.36)	28.12 (23.12, 33.43)	0.648	0.06[b]
A AD-AB ROM (°)	7.13 (4.48, 9.03)	7.78 (4.51, 9.19)	0.799	0.03[b]
A IR-ER ROM (°)	29.52 (23.46, 35.56)	30.85 (27.58, 34.76)	0.083	0.22[b]
H F-E ROM (°)	43.60 (40.70, 46.61)	43.33 (40.39, 48.33)	0.383	0.11[b]
H AD-AB ROM (°)	12.12 ± 3.09	12.43 ± 3.16	0.736	0.10[a]
H IR-ER ROM (°)	41.12 (28.57, 52.63)	32.61 (27.20, 37.03)	0.037*	0.26[b]
K F-E ROM (°)	42.77 (35.08, 48.90)	57.75 (52.70, 60.99)	**<0.001*** **	0.58[b]
K AD-AB ROM (°)	39.15 (27.71, 46.82)	21.06 (13.58, 26.58)	**<0.001*** **	0.59[b]
K IR-ER ROM (°)	19.19 (13.58, 23.40)	18.30 (13.58, 24.25)	0.914	0.01[b]
UnAffected/Dominant Side				
A F-E ROM (°)	27.52 (22.24, 32.53)	28.70 (22.12, 34.26)	0.232	0.15[b]
A AD-AB ROM (°)	5.77 (4.62, 12.34)	5.77 (5.05, 8.50)	0.727	0.04[b]
A IR-ER ROM (°)	31.18 (23.46, 44.81)	26.50 (23.81, 30.56)	0.068	0.23[b]
H F-E ROM (°)	45.01 (41.66, 48.69)	43.00 (38.93, 45.53)	0.020*	0.29[b]
H AD-AB ROM (°)	11.82 (9.54, 13.81)	12.99 (9.43, 16.34)	0.485	0.09[b]
H IR-ER ROM (°)	38.76 (28.00, 52.97)	29.09 (26.28, 32.14)	0.003**	0.37[b]
K F-E ROM (°)	59.40 (55.50, 64.39)	56.88 (51.12, 58.65)	0.007**	0.34[b]
K AD-AB ROM (°)	25.34 (18.07, 35.08)	30.19 (27.39, 32.24)	0.207	0.16[b]
K IR-ER ROM (°)	19.43 (14.23, 25.90)	16.13 (13.26, 20.82)	0.308	0.13[b]

Note:

Data presented as median (Q1, Q3) unless otherwise noted.

Bonferroni corrected α = 0.003 for 18 comparisons

***P < 0.001; **P < 0.01; *P < 0.05

ROM: Range of Motion; °: degrees

[a]Effect size reported as Cohen's d; [b]Effect size reported as r value

Abbreviations:

A: Ankle; H: Hip; K: Knee; F-E: Flexion-Extension; AD-AB: Adduction-Abduction; IR-ER: Internal-External Rotation; ROM: Range of Motion

Supplementary Table S14

Comparison of Peak Joint Moments During Dual-Task Walking Between Lower Functional Outcome and Higher Functional Outcome Groups

Variable	Group		*P*-value	EffectSize
	**Lower Functional Outcome(n=32)**	**Higher Functional Outcome(n=32)**		
Affected/Non-dominant Side				
PAFM (N·mm/kg)	1476.56 (1112.41, 1853.25)	1339.56 (1214.78, 1519.87)	0.925	0.01[b]
PAAM (N·mm/kg)	165.52 (65.89, 261.99)	103.91 (65.33, 154.76)	0.024*	0.28[b]
PAIRM (N·mm/kg)	211.26 (106.18, 287.28)	224.88 (161.22, 270.86)	0.477	0.09[b]
PHFM (N·mm/kg)	809.96 (529.95, 1211.77)	827.25 (556.26, 1039.20)	0.555	0.07[b]
PHAM (N·mm/kg)	1149.20 (775.53, 1463.82)	959.57 (744.95, 1225.08)	0.066	0.23[b]
PHIRM (N·mm/kg)	187.53 (153.54, 232.41)	195.69 (153.37, 238.74)	0.946	0.01[b]
PKFM (N·mm/kg)	262.51 (0.00, 640.23)	737.91 (229.90, 1161.78)	**<0.001*****	0.54[b]
PKAM (N·mm/kg)	619.99 (353.35, 1004.50)	604.45 (435.08, 776.37)	0.957	0.01[b]
PKIRM (N·mm/kg)	187.42 (134.46, 259.42)	186.11 (155.03, 228.94)	0.819	0.03[b]
UnAffected/Dominant Side				
PAFM (N·mm/kg)	1318.54 (1166.22, 1568.06)	1452.61 (1202.50, 1573.66)	0.707	0.05[b]
PAAM (N·mm/kg)	136.63 (39.96, 197.31)	125.28 (79.50, 214.69)	0.936	0.01[b]
PAIRM (N·mm/kg)	245.22 (199.54, 278.53)	204.87 (144.19, 279.40)	0.155	0.18[b]
PHFM (N·mm/kg)	671.16 (555.65, 786.96)	676.77 (575.00, 769.60)	0.809	0.03[b]
PHAM (N·mm/kg)	932.80 (585.19, 1237.69)	1215.52 (947.23, 1291.77)	0.044*	0.25[b]
PHIRM (N·mm/kg)	179.01 (145.15, 203.60)	180.12 (139.52, 246.38)	0.799	0.03[b]
PKFM (N·mm/kg)	501.98 (409.59, 611.96)	464.43 (268.52, 932.21)	0.778	0.04[b]
PKAM (N·mm/kg)	487.36 (364.88, 717.29)	539.96 (439.51, 706.72)	0.629	0.06[b]
PKIRM (N·mm/kg)	212.70 (182.36, 264.34)	188.75 (153.63, 269.77)	0.179	0.17[b]

Note:

Data presented as median (Q1, Q3).

Bonferroni corrected α = 0.003 for 18 comparisons

***P < 0.001; **P < 0.01; *P < 0.05

[a]Effect size reported as Cohen's d; [b]Effect size reported as r value

Abbreviations:

PAFM: Peak ankle flexion moment; PAAM: Peak ankle adduction moment; PAIRM: Peak ankle internal rotation moment;

PHFM: Peak hip flexion moment; PHAM: Peak hip adduction moment; PHIRM: Peak hip internal rotation moment;

PKFM: Peak knee flexion moment; PKAM: Peak knee adduction moment; PKIRM: Peak knee internal rotation moment

Supplementary Table S15

Comparison of Joint Angles and Range of Motion Between Affected and Unaffected Sides During Dual-Task Walking in Lower Functional Outcome Group

Variable	Side		***P*-value**	**EffectSize**
	**AffectedSide(n=32)**	**UnaffectedSide(n=32)**		
Ankle Joint				
ADF (°)	12.96 (8.66, 17.17)	14.99 (12.14, 18.11)	0.036*	0.26[b]
APF (°)	-12.61 (-19.25, -9.89)	-13.58 (-15.29, -9.76)	0.243	0.15[b]
A F-E ROM (°)	25.54 (23.37, 30.34)	28.27 (21.85, 32.65)	0.601	0.07[b]
AAD (°)	6.46 (4.76, 7.70)	4.44 (3.66, 6.55)	0.058	0.24[b]
AAB (°)	0.28 (-4.11, 1.32)	-3.35 (-7.79, -0.50)	0.001**	0.42[b]
A AD-AB ROM (°)	5.35 (4.47, 9.84)	5.77 (4.62, 12.41)	0.638	0.06[b]
AIR (°)	-7.07 (-12.11, 9.08)	6.05 (0.07, 27.31)	**<0.001*** **	0.46[b]
AER (°)	-35.47 (-41.43, -23.24)	-24.04 (-27.65, -18.26)	**<0.001*** **	0.47[b]
A IR-ER ROM (°)	27.48 (22.00, 34.51)	31.18 (22.51, 46.37)	0.277	0.14[b]
Hip Joint				
HF (°)	21.10 (13.95, 30.94)	26.15 (19.92, 33.11)	0.214	0.16[b]
HE (°)	-19.25 (-31.22, -17.84)	-17.36 (-30.02, -13.04)	0.179	0.17[b]
H F-E ROM (°)	43.55 ± 4.20	45.01 ± 4.31	0.172	0.35[a]
HAD (°)	5.21 (2.45, 9.06)	6.29 (3.35, 12.48)	0.220	0.16[b]
HAB (°)	-6.73 (-9.98, -3.99)	-3.93 (-9.82, -0.46)	0.076	0.22[b]
H AD-AB ROM (°)	11.62 (9.17, 15.00)	11.82 (9.52, 13.83)	0.936	0.01[b]
HIR (°)	49.65 (30.46, 55.69)	26.24 (10.24, 30.33)	**<0.001*** **	0.73[b]
HER (°)	0.29 (-10.31, 16.70)	-19.51 (-22.38, -12.62)	**<0.001*** **	0.59[b]
H IR-ER ROM (°)	34.36 (30.94, 54.52)	38.76 (27.70, 53.13)	0.658	0.06[b]
Knee Joint				
Peak KF (°)	35.36 (28.04, 50.23)	59.50 (49.03, 61.93)	**<0.001*** **	0.75[b]
Peak KE (°)	-4.24 (-7.70, -2.57)	-4.35 (-6.99, -1.62)	0.340	0.12[b]
K F-E ROM (°)	40.16 (32.49, 55.86)	59.40 (55.36, 64.43)	**<0.001*** **	0.66[b]
Peak KAD (°)	31.93 (23.91, 54.47)	22.41 (16.44, 32.12)	**<0.001*** **	0.55[b]

Peak KAB (°)	-3.50 (-5.90, -0.02)	-3.52 (-4.22, -2.36)	0.778	0.04[b]
K AD-AB ROM (°)	36.70 (27.98, 54.54)	25.34 (17.90, 35.24)	**<0.001*** **	0.44[b]
Peak KIR (°)	-1.30 (-3.93, 1.22)	7.11 (3.03, 12.22)	**<0.001*** **	0.53[b]
Peak KER (°)	-22.63 (-25.74, -10.41)	-9.59 (-18.18, -6.75)	0.001**	0.40[b]
K IR-ER ROM (°)	19.76 (11.34, 23.44)	19.43 (13.98, 26.39)	0.648	0.06[b]

Note:

Data presented as median (Q1, Q3) unless otherwise noted.

Bonferroni corrected α = 0.002 for 27 comparisons.

***P < 0.001; **P < 0.01; *P < 0.05

ROM: Range of Motion; °: degrees

[a]Effect size reported as Cohen's d; [b]Effect size reported as r value

Abbreviations:

ADF: Ankle dorsiflexion; APF: Ankle plantarflexion; AAD: Ankle adduction; AAB: Ankle abduction; AIR: Ankle internal rotation; AER: Ankle external rotation;

HF: Hip flexion; HE: Hip extension; HAD: Hip adduction; HAB: Hip abduction; HIR: Hip internal rotation; HER: Hip external rotation;

KF: Knee flexion; KE: Knee extension; KAD: Knee adduction; KAB: Knee abduction; KIR: Knee internal rotation; KER: Knee external rotation;

A: Ankle; H: Hip; K: Knee; F-E: Flexion-Extension; AD-AB: Adduction-Abduction; IR-ER: Internal-External Rotation; ROM: Range of Motion

Supplementary Table S16

Comparison of Joint Moments Between Affected and Unaffected Sides During Dual-Task Walking in Lower Functional Outcome Group

Variable	Side		*P*-value	EffectSize
	AffectedSide(n=32)	UnaffectedSide(n=32)		
Ankle Joint				
PAFM (N·mm/kg)	1462.53 (1105.84, 1636.35)	1318.53 (1157.31, 1617.94)	0.272	0.14[b]
PAAM (N·mm/kg)	143.64 (96.24, 204.09)	136.63 (39.64, 200.98)	0.311	0.13[b]
PAIRM (N·mm/kg)	195.78 (123.75, 271.35)	245.22 (199.15, 284.49)	0.306	0.13[b]
Hip Joint				
PHFM (N·mm/kg)	804.91 (666.03, 1155.72)	671.16 (555.65, 786.96)	0.136	0.19[b]
PHAM (N·mm/kg)	1117.51 (907.09, 1364.87)	932.80 (585.19, 1237.69)	0.158	0.18[b]
PHIRM (N·mm/kg)	194.36 (150.39, 240.95)	179.01 (145.15, 203.60)	0.957	0.01[b]
Knee Joint				
PKFM (N·mm/kg)	199.62 (120.98, 301.33)	501.98 (399.29, 627.29)	0.133	0.19[b]
PKAM (N·mm/kg)	641.86 (351.03, 817.86)	487.36 (363.64, 742.97)	0.266	0.14[b]
PKIRM (N·mm/kg)	179.93 (132.30, 230.18)	212.70 (182.20, 265.50)	0.112	0.20[b]

Note:

Data presented as median (Q1, Q3).

Bonferroni corrected α = 0.006 for 9 comparisons.

**P < 0.01; *P < 0.05

Nmm/kg: Newton-millimeters per kilogram

[a]Effect size reported as Cohen's d; [b]Effect size reported as r value

Abbreviations:

PAFM: Peak ankle flexion moment; PAAM: Peak ankle adduction moment; PAIRM: Peak ankle internal rotation moment;

PHFM: Peak hip flexion moment; PHAM: Peak hip adduction moment; PHIRM: Peak hip internal rotation moment;

PKFM: Peak knee flexion moment; PKAM: Peak knee adduction moment; PKIRM: Peak knee internal rotation moment

Balance Task

Supplementary Table S17

Comparison of Joint Flexion/Adduction/Internal Rotation Angles During Balance Task Walking Between Lower Functional Outcome and Higher Functional Outcome Groups

Variable	Group		*P*-value	EffectSize
	Lower Functional Outcome(n=32)	Higher Functional Outcome(n=32)		
Affected/Non-dominant Side				
ADF (°)	11.49 (5.84, 16.45)	15.23 (11.74, 17.88)	0.018*	0.30[b]
AAD (°)	3.22 (1.73, 6.31)	4.99 (3.61, 5.96)	0.788	0.03[b]
AIR (°)	-1.02 (-4.96, 27.35)	12.07 (2.72, 17.61)	0.015*	0.31[b]
HF (°)	28.66 (21.44, 30.44)	21.04 (5.30, 24.14)	0.021*	0.29[b]
HAD (°)	8.37 (4.79, 12.60)	7.49 (4.96, 12.37)	0.004**	0.36[b]
HIR (°)	26.70 (-4.62, 35.52)	23.84 (6.34, 46.48)	0.025*	0.28[b]
KF (°)	55.49 (53.03, 58.02)	51.17 (49.33, 60.77)	**<0.001*** **	0.63[b]
KAD (°)	24.22 (9.09, 27.96)	21.46 (6.10, 30.67)	**<0.001*** **	0.70[b]
KIR (°)	11.04 (4.36, 23.66)	3.01 (-0.40, 4.94)	**<0.001*** **	0.69[b]
UnAffected/Dominant Side				
ADF (°)	15.86 ± 5.37	17.06 ± 2.84	0.267	0.28[a]
AAD (°)	5.00 (3.40, 6.62)	5.88 (5.15, 6.58)	0.110	0.20[b]
AIR (°)	-0.25 (-4.96, 27.35)	12.07 (2.72, 17.61)	0.347	0.12[b]
HF (°)	28.66 (21.44, 30.44)	21.04 (5.30, 24.14)	**<0.001*** **	0.53[b]
HAD (°)	8.37 (4.79, 12.60)	7.49 (4.96, 12.37)	1.000	0.00[b]
HIR (°)	16.54 ± 22.81	25.55 ± 17.47	0.081	0.44[a]
KF (°)	55.49 (53.03, 58.02)	51.17 (49.33, 60.77)	0.076	0.22[b]
KAD (°)	24.22 (9.09, 27.96)	21.46 (6.10, 30.67)	0.519	0.08[b]

KIR (°)	11.04 (4.36, 23.66)	3.01 (-0.40, 4.94)	**<0.001*** **	0.52[b]

Note:

Data presented as median (Q1, Q3) unless otherwise noted.

Bonferroni corrected α = 0.003 for 18 comparisons

***P < 0.001; **P < 0.01; *P < 0.05

°: degrees

[a]Effect size reported as Cohen's d; [b]Effect size reported as r value

Abbreviations:

ADF: Ankle dorsiflexion; AAD: Ankle adduction; AIR: Ankle internal rotation;

HF: Hip flexion; HAD: Hip adduction; HIR: Hip internal rotation;

KF: Knee flexion; KAD: Knee adduction; KIR: Knee internal rotation

Supplementary Table S18

Comparison of Joint Extension/Abduction/External Rotation Angles During Balance Task Walking Between Lower Functional Outcome and Higher Functional Outcome Groups

Variable	Group		*P*-value	EffectSize
	Lower Functional Outcome(n=32)	Higher Functional Outcome(n=32)		
Affected/Non-dominant Side				
APF (°)	-18.91 (-24.19, -13.32)	-12.18 (-16.81, -7.49)	**<0.001*** **	0.46[b]
AAB (°)	-4.95 (-7.52, 1.54)	-5.02 (-6.11, -4.09)	0.005**	0.35[b]
AER (°)	-17.36 (-20.35, -9.81)	-10.71 (-13.02, -9.15)	0.107	0.20[b]
HE (°)	-14.09 (-25.51, -13.00)	-24.16 (-32.82, -21.66)	**<0.001*** **	0.45[b]
HAB (°)	-2.11 (-5.50, 1.93)	-5.35 (-8.18, -4.25)	0.248	0.14[b]
HER (°)	-17.78 (-31.34, 3.69)	-6.74 (-13.18, 19.15)	**<0.001*** **	0.64[b]
KE (°)	2.55 (-7.92, 6.90)	-2.75 (-5.75, -0.33)	**<0.001*** **	0.53[b]
KAB (°)	-3.36 (-5.40, 3.21)	-4.61 (-6.64, 0.31)	0.052	0.24[b]
KER (°)	-5.06 (-8.13, 10.58)	-21.18 (-22.81, -10.48)	0.017*	0.30[b]
UnAffected/Dominant Side				
APF (°)	-13.08 (-16.68, -10.33)	-13.01 (-17.12, -9.31)	0.757	0.04[b]
AAB (°)	-0.47 (-6.19, 0.35)	-4.65 (-7.70, -1.09)	0.064	0.23[b]
AER (°)	-25.74 (-32.78, -19.83)	-20.49 (-27.15, -18.96)	0.099	0.21[b]
HE (°)	-14.09 (-25.51, -13.00)	-24.16 (-32.82, -21.66)	**<0.001*** **	0.51[b]
HAB (°)	-2.11 (-5.50, 1.93)	-5.35 (-8.18, -4.25)	**<0.001*** **	0.44[b]
HER (°)	-17.78 (-31.34, 3.69)	-6.74 (-13.18, 19.15)	0.004**	0.36[b]
KE (°)	2.55 (-7.92, 6.90)	-2.75 (-5.75, -0.33)	0.485	0.09[b]
KAB (°)	-3.36 (-5.40, 3.21)	-4.61 (-6.64, 0.31)	0.039*	0.26[b]
KER (°)	-5.06 (-8.13, 10.58)	-21.18 (-22.81, -10.48)	**<0.001*** **	0.63[b]

Note:

Data presented as median (Q1, Q3).

Bonferroni corrected α = 0.003 for 18 comparisons

***P < 0.001; **P < 0.01; *P < 0.05

°: degrees

Negative values indicate motion opposite to the corresponding positive direction (extension vs. flexion, abduction vs. adduction, external vs. internal rotation)

[a]Effect size reported as Cohen's d; [b]Effect size reported as r value

Abbreviations:

APF: Ankle plantarflexion; AAB: Ankle abduction; AER: Ankle external rotation;

HE: Hip extension; HAB: Hip abduction; HER: Hip external rotation;

KE: Knee extension; KAB: Knee abduction; KER: Knee external rotation

Supplementary Table S19

Comparison of Joint Range of Motion During Balance Task Walking Between Lower Functional Outcome and Higher Functional Outcome Groups

Variable	Group		*P*-value	EffectSize
	**Lower Functional Outcome(n=32)**	**Higher Functional Outcome(n=32)**		
Affected/Non-dominant Side				
A F-E ROM (°)	29.86 (24.71, 34.24)	26.42 (22.76, 31.23)	0.020*	0.29[b]
A AD-AB ROM (°)	7.30 (5.38, 9.30)	10.08 (7.53, 12.43)	0.001**	0.43[b]
A IR-ER ROM (°)	29.31 ± 6.59	39.37 ± 5.75	**<0.001*** **	1.63[a]
H F-E ROM (°)	44.35 ± 3.78	44.64 ± 4.38	0.774	0.07[a]
H AD-AB ROM (°)	10.05 (8.94, 12.02)	15.61 (11.33, 16.25)	**<0.001*** **	0.48[b]
H IR-ER ROM (°)	27.66 (24.79, 37.20)	28.93 (25.00, 33.90)	0.193	0.16[b]
K F-E ROM (°)	55.43 (50.58, 61.88)	55.92 (53.35, 59.81)	0.007**	0.34[b]
K AD-AB ROM (°)	22.59 (17.41, 25.41)	27.86 (25.71, 30.86)	**<0.001*** **	0.45[b]
K IR-ER ROM (°)	15.85 ± 5.76	22.68 ± 7.19	**<0.001*** **	1.05[a]
UnAffected/Dominant Side				
A F-E ROM (°)	29.32 ± 6.24	30.24 ± 5.89	0.546	0.15[a]
A AD-AB ROM (°)	5.51 (4.53, 8.62)	9.39 (6.26, 12.51)	**<0.001*** **	0.45[b]
A IR-ER ROM (°)	28.21 (23.57, 34.86)	32.51 (26.83, 34.73)	0.140	0.18[b]
H F-E ROM (°)	42.84 (40.33, 47.25)	42.62 (38.85, 44.95)	0.405	0.10[b]
H AD-AB ROM (°)	10.57 ± 2.88	14.18 ± 2.78	**<0.001*** **	1.27[a]
H IR-ER ROM (°)	27.66 (24.79, 37.20)	28.93 (25.00, 33.90)	0.778	0.04[b]
K F-E ROM (°)	55.88 ± 6.28	56.27 ± 4.33	0.772	0.07[a]
K AD-AB ROM (°)	22.59 (17.41, 25.41)	27.86 (25.71, 30.86)	0.009**	0.33[b]
K IR-ER ROM (°)	16.08 (10.56, 19.70)	22.32 (16.61, 24.69)	**<0.001*** **	0.46[b]

Note:

Data presented as median (Q1, Q3) unless otherwise noted.

Bonferroni corrected α = 0.003 for 18 comparisons

***P < 0.001; **P < 0.01; *P < 0.05

ROM: Range of Motion; °: degrees

[a]Effect size reported as Cohen's d; [b]Effect size reported as r value

Abbreviations:

A: Ankle; H: Hip; K: Knee; F-E: Flexion-Extension; AD-AB: Adduction-Abduction; IR-ER: Internal-External Rotation; ROM: Range of Motion

Supplementary Table S20

Comparison of Peak Joint Moments During Balance Task Walking Between Lower Functional Outcome and Higher Functional Outcome Groups

Variable	Group		*P*-value	EffectSize
	Lower Functional Outcome(n=32)	Higher Functional Outcome(n=32)		
Affected/Non-dominant Side				
PAFM (N·mm/kg)	1143.88 (987.31, 1260.53)	1455.71 (1203.37, 1543.49)	0.001**	0.43[b]
PAAM (N·mm/kg)	130.93 (79.08, 470.85)	141.19 (98.41, 220.85)	0.001**	0.43[b]
PAIRM (N·mm/kg)	225.71 (178.95, 498.79)	237.41 (187.08, 267.43)	0.116	0.20[b]
PHFM (N·mm/kg)	486.65 (421.68, 717.54)	858.92 (577.51, 1055.76)	0.893	0.02[b]
PHAM (N·mm/kg)	1130.75 ± 652.55	845.01 ± 267.71	0.027*	0.57[a]
PHIRM (N·mm/kg)	262.08 ± 97.09	184.48 ± 44.32	**<0.001*** **	1.03[a]
PKFM (N·mm/kg)	521.69 (406.47, 1866.85)	463.27 (239.46, 713.63)	0.511	0.08[b]
PKAM (N·mm/kg)	489.40 ± 455.81	553.96 ± 167.74	0.055	0.49[a]
PKIRM (N·mm/kg)	247.22 (176.89, 411.86)	216.32 (191.88, 285.05)	0.289	0.13[b]
UnAffected/Dominant Side				

PAFM(N·mm/kg)	1172.49 ± 414.74	1392.70 ± 186.10	0.009**	0.69[a]
PAAM (N·mm/kg)	130.93 (79.08, 470.85)	141.19 (98.41, 220.85)	0.667	0.05[b]
PAIRM(N·mm/kg)	225.71 (178.95, 498.79)	237.41 (187.08, 267.43)	0.485	0.09[b]
PHFM (N·mm/kg)	486.65 (421.68, 717.54)	858.92 (577.51, 1055.76)	**<0.001*** **	0.45[b]
PHAM (N·mm/kg)	1024.17 (611.73, 1528.10)	1226.67 (1007.60, 1409.56)	0.184	0.17[b]
PHIRM(N·mm/kg)	166.28 (135.92, 218.20)	200.80 (164.01, 229.68)	0.031*	0.27[b]
PKFM (N·mm/kg)	521.69 (406.47, 1866.85)	463.27 (239.46, 713.63)	0.129	0.19[b]
PKAM(N·mm/kg)	489.40 (383.18, 1279.22)	553.96 (468.46, 757.83)	0.452	0.09[b]
PKIRM(N·mm/kg)	247.22 (176.89, 411.86)	216.32 (191.88, 285.05)	0.477	0.09[b]

Note:

Data presented as median (Q1, Q3) unless otherwise noted.

Bonferroni corrected α = 0.003 for 18 comparisons

***P < 0.001; **P < 0.01; *P < 0.05

N·m: Newton-meters

[a]Effect size reported as Cohen's d; [b]Effect size reported as r value

Abbreviations:

PAFM: Peak ankle flexion moment; PAAM: Peak ankle adduction moment; PAIRM: Peak ankle internal rotation moment;

PHFM: Peak hip flexion moment; PHAM: Peak hip adduction moment; PHIRM: Peak hip internal rotation moment;

PKFM: Peak knee flexion moment; PKAM: Peak knee adduction moment; PKIRM: Peak knee internal rotation moment

Supplementary Table S21

Between-Group Comparison: Joint Power During Different Walking Tasks

Variable	Normal Walking				Dual Task				Balance Task			
	Lower Functional Outcome (n=32)	Higher Functional Outcome (n=32)	*p*-Value	ES	Lower Functional Outcome (n=32)	Higher Functional Outcome (n=32)	*p*-Value	ES	Lower Functional Outcome (n=32)	Higher Functional Outcome (n=32)	*p*-Value	ES
Affected Side												
AJP	2.80 (2.26, 3.09)	3.03 (2.65, 3.26)	0.11	0.198[b]	1.06 (0.88, 2.48)	3.07 (2.53, 3.60)	**<0.001*** **	**0.572[b]**	2.81 (2.53, 3.10)	3.22 (2.73, 3.93)	0.048*	0.247[b]
HJP	0.93 (0.77, 1.33)	1.25 (0.86, 6.73)	0.06	0.238[b]	0.79 (0.51, 1.69)	0.98 (0.77, 1.45)	0.28	0.136[b]	1.45 (0.91, 1.88)	1.41 (0.92, 8.77)	0.25	0.144[b]
KJP	0.50 (0.33, 0.69)	0.65 (0.53, 1.31)	0.011*	0.317[b]	0.56 (0.41, 0.66)	0.55 (0.43, 0.89)	0.63	0.060[b]	0.60 (0.44, 0.83)	0.45 (0.31, 1.06)	0.22	0.153[b]
Unaffected Side												
AJP	2.92 (2.64, 3.70)	3.11 (2.54, 3.39)	0.77	0.037[b]	2.46 (2.11, 3.08)	2.93 (2.47, 3.33)	0.013*	0.310[b]	2.83 (2.55, 3.19)	3.16 (2.43, 3.67)	0.13	0.188[b]
HJP	1.03 (0.90, 1.74)	1.34 (0.97, 3.82)	0.21	0.156[b]	1.14 (0.96, 1.30)	1.09 (0.87, 1.41)	0.64	0.059[b]	1.44 (1.25, 2.23)	1.19 (0.94, 6.38)	0.20	0.160[b]
KJP	0.33 (0.24, 0.55)	0.65 (0.48, 1.02)	**0.004** **	0.364[b]	0.39 (0.28, 0.67)	0.47 (0.25, 0.61)	0.87	0.020[b]	0.71 (0.54, 1.07)	0.30 (0.15, 0.83)	**0.006** **	0.346[b]

Note:

Data are presented as mean ± SD or median (Q1, Q3).

Bonferroni corrected α = 0.008 for 6 comparisons per condition

[a]Effect size reported as Cohen's d; [b]Effect size reported as r value

*P < 0.05; **P < 0.01; ***P < 0.001

Statistical analysis: Independent t-test for normally distributed data; Mann-Whitney U test for non-normally distributed data

Normality was assessed using the Shapiro-Wilk test (P > 0.05 indicates normal distribution)

Abbreviations:

AJP: Ankle Joint Power; HJP: Hip Joint Power; KJP: Knee Joint Power

Supplementary Table S22

Within-Group Comparison in Lower Functional Outcome Group: Spatiotemporal Parameters During Different Walking Tasks (Affected Side vs Unaffected Side)

Variable	Normal Walking				Dual Task				Balance Task			
	Affected (n=32)	Unaffected (n=32)	P-value	Effect Size	Affected (n=32)	Unaffected (n=32)	P-value	Effect Size	Affected (n=32)	Unaffected (n=32)	P-value	Effect Size
Cadence	98.47 ± 9.10	98.78 ± 9.29	0.892	-0.03[a]	99.30 (86.82, 105.07)	103.43 (95.46, 112.18)	0.034*	0.27[b]	92.99 (86.30, 105.75)	89.52 (84.36, 101.89)	0.386	0.11[b]
Step Length	0.64	0.63 (0.61,	0.037*	0.26[b]	0.62	0.64 (0.59,	0.383	0.11[b]	0.62 ±	0.63 ± 0.04	0.606	-0.13[a]

Variable	Normal Walking				Dual Task				Balance Task			
	**Affected (n=32)**	**Unaffected (n=32)**	**P-value**	**Effect Size**	**Affected (n=32)**	**Unaffected (n=32)**	**P-value**	**Effect Size**	**Affected (n=32)**	**Unaffected (n=32)**	**P-value**	**Effect Size**
	(0.62, 0.65)	0.64)			(0.60, 0.67)	0.65)			0.03			
Step Width	0.14 (0.11, 0.21)	0.15 (0.11, 0.20)	0.778	0.04[b]	0.13 ± 0.05	0.13 ± 0.03	0.912	0.03[a]	0.14 (0.10, 0.21)	0.14 (0.09, 0.17)	0.271	0.14[b]
Stride Length	1.25 ± 0.06	1.25 ± 0.06	0.993	0.00[a]	1.23 (1.17, 1.32)	1.27 (1.20, 1.31)	0.320	0.12[b]	1.23 ± 0.06	1.26 ± 0.05	0.022*	-0.59[a]
Walking Speed	1.03 ± 0.12	1.03 ± 0.11	0.929	-0.02[a]	1.01 ± 0.15	1.09 ± 0.12	0.030*	-0.55[a]	0.92 (0.88, 1.07)	0.93 (0.87, 1.07)	0.819	0.03[b]

Note:

Data are presented as mean ± SD or median (Q1, Q3).

Bonferroni corrected α = 0.01 for 5 comparisons per condition.

[a]Effect size reported as Cohen's d; [b]Effect size reported as r value

*P < 0.05; **P < 0.01; ***P < 0.001

Statistical analysis: Paired t-test for normally distributed data; Wilcoxon signed-rank test for non-normally distributed data

Comparison is within the same Lower Functional Outcome group, comparing affected side with unaffected side

Supplementary Table S23

Within-Group Comparison in Lower Functional Outcome Group: Foot Progression Angles During Different Walking Tasks (Affected Side vs Unaffected Side)

Variable	Normal Walking				Dual Task				Balance Task			
	Affected (n=32)	Unaffected (n=32)	P-value	Effect Size	Affected (n=32)	Unaffected (n=32)	P-value	Effect Size	Affected (n=32)	Unaffected (n=32)	P-value	Effect Size
FPA-X Max	-21.16 (-29.01, -15.14)	-23.25 (-26.78, -20.63)	0.197	0.16[b]	-24.30 (-30.67, -20.36)	-26.50 (-32.71, -22.15)	0.243	0.15[b]	-23.43 (-28.53, -16.05)	-25.10 (-29.63, -19.65)	0.227	0.15[b]
FPA-X Min	-112.97 ± 6.82	-114.26 ± 4.08	0.365	0.23[a]	-107.59 (-113.85, -106.48)	-115.08 (-117.90, -107.89)	0.007**	0.34[b]	-109.41 (-113.67, -106.09)	-107.82 (-110.68, -104.08)	0.209	0.16[b]
FPA-X ROM	91.43 ± 15.11	90.37 ± 5.11	0.708	0.09[a]	83.14 ± 7.19	85.67 ± 6.12	0.135	-0.38[a]	86.19 (82.48, 89.40)	83.09 (78.80, 88.73)	0.062	0.23[b]
FPA-Y Max	8.34 (3.71, 13.57)	7.43 (5.93, 10.98)	0.546	0.08[b]	5.68 (4.15, 9.37)	10.26 (4.77, 14.23)	0.012*	0.32[b]	11.47 (9.87, 13.91)	10.66 (8.05, 14.00)	0.444	0.10[b]
FPA-Y Min	-2.67 (-5.31, 2.60)	-1.80 (-5.31, 1.22)	0.460	0.09[b]	-4.09 (-5.02, -1.78)	-2.08 (-5.85, -1.08)	0.564	0.07[b]	-3.85 ± 5.02	-4.05 ± 2.76	0.848	0.05[a]
FPA-Y ROM	10.22 (8.16, 13.79)	11.14 (6.45, 12.61)	0.830	0.03[b]	8.94 (7.32, 12.08)	12.76 (11.36, 15.44)	0.007**	0.34[b]	15.63 ± 4.42	15.33 ± 2.36	0.738	0.08[a]
FPA-Z Max	-0.56 (-2.75, 3.34)	-2.26 (-5.38, 1.67)	0.113	0.20[b]	-0.12 ± 3.80	-0.71 ± 5.69	0.624	0.12[a]	3.00 (-0.07, 9.99)	2.98 (0.35, 7.06)	0.830	0.03[b]
FPA-Z Min	-14.26 ± 6.09	-16.06 ± 6.20	0.246	0.29[a]	-11.66 ± 4.56	-12.75 ± 6.03	0.415	0.21[a]	-13.21 ± 4.99	-12.91 ± 5.80	0.822	-0.06[a]
FPA-Z ROM	14.41 (11.19, 17.73)	13.67 (10.72, 17.26)	0.861	0.02[b]	11.01 (9.97, 13.24)	11.29 (7.35, 16.26)	0.861	0.02[b]	17.95 ± 4.48	18.46 ± 4.66	0.657	-0.11[a]

Note:

Data are presented as mean ± SD or median (Q1, Q3).

Bonferroni corrected α = 0.006 for 9 comparisons per condition.

[a]Effect size reported as Cohen's d; [b]Effect size reported as r value

*P < 0.05; **P < 0.01; ***P < 0.001

Statistical analysis: Paired t-test for normally distributed data; Wilcoxon signed-rank test for non-normally distributed data

Comparison is within the same Lower Functional Outcome group, comparing affected side with unaffected side

Abbreviations:

FPA-X Max: Foot Progression Angle X (Max)

FPA-X Min: Foot Progression Angle X (Min)

FPA-X ROM: Foot Progression Angle X (ROM)

FPA-Y Max: Foot Progression Angle Y (Max)

FPA-Y Min: Foot Progression Angle Y (Min)

FPA-Y ROM: Foot Progression Angle Y (ROM)

FPA-Z Max: Foot Progression Angle Z (Max)

FPA-Z Min: Foot Progression Angle Z (Min)

FPA-Z ROM: Foot Progression Angle Z (ROM)

Supplementary Table S24

Within-Group Comparison in Lower Functional Outcome Group: Pelvis Tilt Angles During Different Walking Tasks (Affected Side vs Unaffected Side)

Variable	Normal Walking				Dual Task				Balance Task			
	Affected (n=32)	Unaffected (n=32)	P-value	Effect Size	Affected (n=32)	Unaffected (n=32)	P-value	Effect Size	Affected (n=32)	Unaffected (n=32)	P-value	Effect Size
PTA-X Max	4.10 (-5.51, 8.82)	3.91 (-9.48, 8.94)	0.682	0.05[b]	1.33 (-5.51, 4.23)	1.58 (-7.14, 5.04)	0.973	0.00[b]	1.26 (-7.58, 4.61)	1.73 (-9.09, 5.57)	0.825	0.03[b]
PTA-X Min	-0.24 (-11.29, 4.03)	0.58 (-13.97, 4.05)	0.963	0.01[b]	-1.66 (-12.01, 1.36)	-1.99 (-12.36, 1.54)	0.936	0.01[b]	-3.97 (-13.72, 1.37)	-5.06 (-13.95, 1.18)	0.877	0.02[b]
PTA-X ROM	4.81 (3.70, 5.87)	4.22 (3.20, 5.93)	0.361	0.11[b]	3.79 (2.80, 4.28)	3.93 (3.27, 4.60)	0.424	0.10[b]	4.87 (3.54, 5.53)	4.59 (3.57, 6.23)	0.788	0.03[b]
PTA-Y Max	3.09 (-0.06, 4.12)	3.64 (1.38, 6.04)	0.050	0.25[b]	4.65 (2.81, 6.42)	2.22 (1.31, 3.49)	**0.004****	0.36[b]	2.12 ± 2.14	4.19 ± 2.12	**<0.001*****	-0.97[a]
PTA-Y Min	-3.80 (-6.13, -1.30)	-2.86 (-3.90, 0.14)	0.050	0.25[b]	-2.19 (-3.41, -1.53)	-3.69 (-6.20, -2.10)	0.033*	0.27[b]	-3.95 ± 2.33	-1.92 ± 1.82	**<0.001*****	-0.97[a]
PTA-Y ROM	6.10 ± 2.01	5.96 ± 1.99	0.781	0.07[a]	6.87 ± 1.97	6.44 ± 2.12	0.407	0.21[a]	5.35 (4.23, 7.33)	5.53 (4.25, 7.88)	0.697	0.05[b]
PTA-Z Max	7.20 ± 5.63	6.15 ± 4.58	0.418	0.20[a]	5.53 (2.38, 8.96)	7.30 (5.00, 9.04)	0.171	0.17[b]	9.28 (6.49, 14.35)	9.35 (4.54, 11.50)	0.167	0.17[b]
PTA-Z Min	-5.49 ± 4.35	-6.16 ± 5.08	0.570	0.14[a]	-5.99 (-8.29, -4.49)	-4.43 (-11.80, -2.40)	0.638	0.06[b]	-8.87 (-11.50, -4.77)	-9.54 (-12.20, -7.11)	0.179	0.17[b]
PTA-Z ROM	12.68 ± 4.00	12.31 ± 3.43	0.692	0.10[a]	12.64 (6.64, 16.37)	12.51 (7.18, 18.79)	0.537	0.08[b]	17.61 ± 3.64	17.17 ± 4.37	0.668	0.11[a]

Note:

Data are presented as mean ± SD or median (Q1, Q3).

Bonferroni corrected α = 0.006 for 9 comparisons per condition.

[a]Effect size reported as Cohen's d; [b]Effect size reported as r value

*P < 0.05; **P < 0.01; ***P < 0.001

Statistical analysis: Paired t-test for normally distributed data; Wilcoxon signed-rank test for non-normally distributed data

Comparison is within the same Lower Functional Outcome group, comparing affected side with unaffected side

Abbreviations:

PTA-X Max: Pelvis Tilt Angle X (Max)

PTA-X Min: Pelvis Tilt Angle X (Min)

PTA-X ROM: Pelvis Tilt Angle X (ROM)

PTA-Y Max: Pelvis Tilt Angle Y (Max)

PTA-Y Min: Pelvis Tilt Angle Y (Min)

PTA-Y ROM: Pelvis Tilt Angle Y (ROM)

PTA-Z Max: Pelvis Tilt Angle Z (Max)

PTA-Z Min: Pelvis Tilt Angle Z (Min)

PTA-Z ROM: Pelvis Tilt Angle Z (ROM)

Supplementary Table S25

Within-Group Comparison: Joint Power During Different Walking Tasks (Affected Side vs Unaffected Side)

Variable	Normal Walking				Dual Task				Balance Task			
	**Affected (n=32)**	**Unaffected (n=32)**	**P-value**	**Effect Size**	**Affected (n=32)**	**Unaffected (n=32)**	**P-value**	**Effect Size**	**Affected (n=32)**	**Unaffected (n=32)**	**P-value**	**Effect Size**
AJP	2.80 (2.20, 3.10)	2.92 (2.62, 3.73)	0.057	0.24[b]	3.07 (2.52, 3.65)	2.93 (2.46, 3.34)	0.428	0.10[b]	3.22 (2.68, 3.95)	2.83 (2.54, 3.19)	0.030*	0.27[b]
HJP	1.25 (0.86, 6.78)	1.34 (0.97, 4.08)	0.799	0.03[b]	0.98 (0.76, 1.45)	1.09 (0.87, 1.42)	0.436	0.10[b]	1.41 (0.92, 8.95)	1.19 (0.94, 6.52)	0.334	0.12[b]
KJP	0.65 (0.52, 1.33)	0.65 (0.48, 1.06)	0.493	0.09[b]	0.55 (0.43, 0.89)	0.47 (0.23, 0.61)	0.086	0.22[b]	0.45 (0.29, 1.13)	0.30 (0.15, 0.84)	0.041*	0.26[b]

Note:

Data are presented as mean ± SD or median (Q1, Q3).

[a]Effect size reported as Cohen's d; [b]Effect size reported as r value

*P < 0.05; **P < 0.01; ***P < 0.001

Statistical analysis: Paired t-test for normally distributed data; Wilcoxon signed-rank test for non-normally distributed data

Comparison is within the same Lower Functional Outcome group, comparing affected side with unaffected side

Abbreviations:

AJP: Ankle Joint Power

HJP: Hip Joint Power

KJP: Knee Joint Power

Normal-Speed walking

Supplementary Table S26

Comparison of Joint Flexion/Adduction/Internal Rotation Angles During Normal - Speed Walking Between Lower Functional Outcome and Higher Functional Outcome Groups

Variable	Group		*P*-value	EffectSize
	**Lower Functional Outcome**	**Higher Functional Outcome**		
Affected/Non-dominant Side				
ADF (°)	14.76 (12.01-16.21)	15.89 (13.99-17.74)	0.083	0.22[b]
AAD (°)	4.37 (1.70-5.82)	5.50 (3.88-7.03)	0.015	0.30[b]
AIR (°)	6.21 (-10.63-22.91)	11.93 (3.81-20.24)	0.804	0.03[b]
HF (°)	26.23 (13.67-30.19)	18.11 (8.74-30.98)	0.207	0.16[b]
HAD (°)	4.94 (2.17-10.34)	3.62 (1.96-7.95)	0.640	0.06[b]
HIR (°)	27.19 (11.51-31.90)	10.53 (1.95-20.29)	0.238	0.15[b]
KF (°)	53.24 (48.85-61.10)	55.52 (51.76-59.42)	0.199	0.16[b]
KAD (°)	20.90 (14.23-25.28)	11.94 (6.54-18.18)	0.023	0.28[b]
KIR (°)	-0.76 (-3.59-13.48)	15.13 (5.15-19.95)	**<0.001*** **	0.46[b]

UnAffected/Dominant Side				
ADF (°)†	15.82 ± 6.14	14.87 ± 2.53	0.420	0.20[a]
AAD (°)	2.93 (-1.26-5.84)	5.55 (3.63-6.86)	0.151	0.18[b]
AIR (°)	10.93 (-0.65-26.06)	10.86 (7.04-13.71)	0.892	0.02[b]
HF (°)	23.53 (14.20-30.62)	18.39 (3.70-32.47)	0.039	0.26[b]
HAD (°)	3.06 (1.30-5.58)	10.01 (6.89-11.22)	**<0.001*** **	0.51[b]
HIR (°)	13.47 (-2.72-17.94)	7.42 (3.10-19.81)	0.883	0.02[b]
KF (°)	61.88 (54.46-64.03)	54.81 (51.05-61.20)	0.017	0.30[b]
KAD (°)	18.95 (12.37-22.84)	12.27 (7.10-17.40)	0.252	0.15[b]
KIR (°)	8.73 ± 13.06	5.17 ± 7.12	0.181	0.34[a]

Data presented as median (interquartile range) unless otherwise noted. Effect size reported as r value unless otherwise noted.

***P < 0.001 (significant after Bonferroni correction)

ADF: Ankle dorsiflexion; AAD: Ankle adduction; AIR: Ankle internal rotation;

HF: Hip flexion; HAD: Hip adduction; HIR: Hip internal rotation;

KF: Knee flexion; KAD: Knee adduction; KIR: Knee internal rotation

Supplementary Table S27

Comparison of Joint Extension/Abduction/External Rotation Angles During Normal Walking Between Lower Functional Outcome and Higher Functional Outcome Groups

Variable	Group		*P*-value	EffectSize
	Lower Functional Outcome	Higher Functional Outcome		
Affected/Non-dominant Side				
APF (°)	-14.57 (-17.72--11.62)	-9.35 (-15.79--5.40)	0.047	0.25[b]
AAB (°)	-3.22 (-6.47--0.72)	-2.79 (-5.75--1.96)	0.024	0.28[b]
AER (°)	-18.63 (-26.46--1.54)	-23.52 (-22.90--15.45)	0.502	0.08[b]
HE (°)	-20.89 (-30.59--15.16)	-23.49 (-35.68--8.95)	0.519	0.08[b]
HAB (°)	-6.20 (-10.42--4.88)	-6.82 (-5.35--2.30)	0.452	0.09[b]
HER (°)	-7.54 (-31.06--7.52)	-13.59 (-22.87--9.10)	0.016	0.30[b]
KE (°)	0.62 (-6.56--0.03)	-1.60 (-6.22--0.90)	0.493	0.09[b]
KAB (°)	-3.15 (-5.32--1.80)	-6.80 (-7.30--0.78)	0.001	0.41[b]
KER (°)	-16.19 ± 11.39	-7.12 ± 5.65	**<0.001*** **	1.01[a]
UnAffected/Dominant Side				
APF (°)	-19.45 (-23.54--13.74)	-16.62 (-21.23--12.97)	0.629	0.06[b]
AAB (°)	-4.83 (-6.47--0.72)	-4.11 (-5.75--1.96)	0.017	0.30[b]
AER (°)	-15.80 (-26.46--1.54)	-18.77 (-22.90--15.45)	0.946	0.01[b]
HE (°)	-22.07 (-30.59--15.16)	-24.49 (-35.68--8.95)	0.123	0.19[b]
HAB (°)	-8.16 (-10.42--4.88)	-3.77 (-5.35--2.30)	**<0.001*** **	0.56[b]
HER (°)	-20.29 (-31.06--7.52)	-18.08 (-22.87--9.10)	0.658	0.06[b]
KE (°)	-3.51 (-6.56--0.03)	-2.71 (-6.22--0.90)	0.021	0.29[b]

KAB (°)	-2.86 (-5.32--1.80)	-5.31 (-7.30--0.78)	0.086	0.22[b]
KER (°)	-13.77 ± 14.12	-14.76 ± 9.12	0.739	0.08[a]

Note:

Data presented as median (interquartile range) unless otherwise noted. Effect size reported as r value unless otherwise noted.
***P < 0.001 (significant after Bonferroni correction)
°: degrees
Negative values indicate motion opposite to the corresponding positive direction (extension vs. flexion, abduction vs. adduction, external vs. internal rotation)

Abbreviations:

APF: Ankle plantarflexion; AAB: Ankle abduction; AER: Ankle external rotation;
HE: Hip extension; HAB: Hip abduction; HER: Hip external rotation;
KE: Knee extension; KAB: Knee abduction; KER: Knee external rotation

Supplementary Table S28

Comparison of Joint Range of Motion Between Lower Functional Outcome and Higher Functional Outcome Groups

Variable	Group		*P*-value	EffectSize
	Lower Functional Outcome	Higher Functional Outcome		
Affected/Non-dominant Side				
A F-E ROM (°)	28.56 (25.59-31.46)	27.15 (24.71-29.94)	0.088	0.21[b]
A AD-AB ROM (°)	6.57 (3.58-9.64)	8.78 (5.84-11.99)	0.013	0.31[b]
A IR-ER ROM (°)	25.13 (18.11-32.45)	31.22 (25.35-37.69)	0.001	0.41[b]
H F-E ROM (°)†	43.48 ± 2.91	42.65 (38.37-44.13)	0.757	0.04[b]
H AD-AB ROM (°)	11.20 (8.93-12.76)	12.61 (11.21-14.22)	0.020	0.29[b]
H IR-ER ROM (°)	31.06 (20.34-39.69)	29.07 (19.62-30.53)	0.914	0.01[b]
K F-E ROM (°)	62.29 (60.99-67.38)	59.53 (54.60-60.04)	0.052	0.24[b]
K AD-AB ROM (°)	24.05 (14.53-22.69)	21.87 (13.45-26.12)	0.072	0.22[b]
K IR-ER ROM (°)	19.51 (18.11-25.07)	17.63 (15.34-21.19)	0.468	0.09[b]
UnAffected/Dominant Side				

A F-E ROM (°)	32.06 (29.27-34.85)	31.50 (28.91-34.11)	0.428	0.10[b]
A AD-AB ROM (°)	7.84 (5.85-9.83)	9.96 (7.70-12.22)	0.159	0.18[b]
A IR-ER ROM (°)	26.74 (18.11-35.37)	29.43 (23.67-35.19)	0.163	0.17[b]
H F-E ROM (°)	44.64 (42.68-47.42)	41.26 (38.37-44.13)	0.002	0.39[b]
H AD-AB ROM (°)	10.92 (8.93-12.76)	13.02 (11.21-14.22)	0.005	0.35[b]
H IR-ER ROM (°)	26.31 (20.34-39.69)	25.87 (19.62-30.53)	0.601	0.07[b]
K F-E ROM (°)	63.78 (60.99-67.38)	57.93 (54.60-60.04)	**<0.001*** **	0.46[b]
K AD-AB ROM (°)	19.74 (14.53-22.69)	14.94 (13.45-26.12)	0.591	0.07[b]
K IR-ER ROM (°)	21.45 (18.11-25.07)	18.65 (15.34-21.19)	0.068	0.23[b]

Note:

Data presented as median (interquartile range) unless otherwise noted. Effect size reported as r value unless otherwise noted.

***P < 0.001 (significant after Bonferroni correction)

ROM: Range of Motion; °: degrees

Abbreviations:

A: Ankle; H: Hip; K: Knee; F-E: Flexion-Extension; AD-AB: Adduction-Abduction; IR-ER: Internal-External Rotation; ROM: Range of Motion

Supplementary Table S29

Comparison of Peak Joint Moments During Normal-Speed Walking Between Lower Functional Outcome and Higher Functional Outcome Groups

Variable	Group		*P*-value	EffectSize
	Lower Functional Outcome(n=32)	Higher Functional Outcome(n=32)		
Affected/Non-dominant Side				
PAFM (N·mm/kg)	1199.99 (1031.84-1492.57)	1372.28 (1317.10-1506.46)	0.489	0.09[b]
PAAM (N·mm/kg)	127.55 (68.03-211.49)	109.89 (70.33-140.07)	0.675	0.05[b]
PAIRM(N·mm/kg)	198.57 (154.59-259.17)	196.08 (164.35-245.22)	0.883	0.02[b]
PHFM(N·mm/kg)	789.41 (614.86-955.60)	662.99 (432.40-975.90)	0.138	0.19[b]
PHAM (N·mm/kg)	953.23 (800.30-1224.19)	816.68 (655.33-1028.40)	0.240	0.15[b]
PHIRM (N·mm/kg)	163.99 (116.30-241.36)	168.10 (145.78-197.73)	0.597	0.07[b]
PKFM (N·mm/kg)	494.58 (268.42-1885.15)	513.85 (346.52-676.38)	0.368	0.11[b]
PKAM (N·mm/kg)	544.06 (477.20-725.89)	513.60 (434.74-639.87)	0.177	0.17[b]
PKIRM(N·mm/kg)	181.24 (149.92-233.73)	175.56 (158.34-210.47)	0.354	0.12[b]
UnAffected/Dominant Side				

PAFM (N·mm/kg)	1297.45 (870.79-1426.62)	1376.88 (1236.24-1439.99)	0.870	0.02[b]
PAAM (N·mm/kg)	127.81 (72.58-182.78)	130.30 (77.06-181.66)	0.574	0.07[b]
PAIRM (N·mm/kg)	223.32 (187.61-256.56)	225.21 (185.01-269.57)	0.706	0.05[b]
PHFM (N·mm/kg)	696.21 (589.54-854.58)	679.16 (467.53-826.37)	0.332	0.12b
PHAM (N·mm/kg)	956.44 (750.50-1071.28)	1172.38 (958.36-1269.90)	0.030*	0.27[b]
PHIRM(N·mm/kg)	214.69 (176.23-260.40)	184.88 (123.73-225.44)	0.771	0.04[b]
PKFM (N·mm/kg)	584.25 (325.98-2371.64)	430.06 (239.88-605.30)	0.161	0.18[b]
PKAM (N·mm/kg)	583.69 (426.45-727.33)	574.96 (444.06-664.29)	0.460	0.09[b]
PKIRM (N·mm/kg)	212.75 (178.81-248.55)	201.48 (173.18-229.23)	0.875	0.02[b]

Note:

Data presented as median (interquartile range) unless otherwise noted. Effect size reported as r value unless otherwise noted.

***P < 0.001 (significant after Bonferroni correction); *P < 0.05

N·mm/kg: Newton-millimeters per kilogram

Abbreviations:

PAFM: Peak ankle flexion moment; PAAM: Peak ankle adduction moment; PAIRM: Peak ankle internal rotation moment;

PHFM: Peak hip flexion moment; PHAM: Peak hip adduction moment; PHIRM: Peak hip internal rotation moment;

PKFM: Peak knee flexion moment; PKAM: Peak knee adduction moment; PKIRM: Peak knee internal rotation moment

Supplementary Table S30

Comparison of Joint Angles and Range of Motion Between Affected and Unaffected Sides During Normal Speed Walking in Lower Functional Outcome Group

Variable	Side		*P*-value	EffectSize
	AffectedSide(n=32)	UnaffectedSide(n=32)		
Ankle Joint				
ADF (°)	14.76 (11.81, 16.22)	15.09 (10.70, 20.86)	0.383	0.109[b]
APF (°)	-14.01 (-17.95, -12.66)	-15.67 (-23.62, -13.69)	0.193	0.163[b]
AAD (°)	4.37 (1.50, 5.94)	2.93 (-1.32, 5.87)	0.327	0.123[b]
AAB (°)	-3.85 (-5.59, 0.82)	-3.28 (-6.47, -0.72)	0.222	0.153[b]
AIR (°)	6.50 ± 17.41	12.05 ± 16.09	0.191	0.331[a]
AER (°)	-21.32 (-31.05, -12.06)	-21.77 (-26.46, 1.54)	0.376	0.111[b]
A F-E ROM (°)	28.31 (25.91, 31.45)	31.61 (27.39, 40.82)	0.025*	0.280[b]
A AD-AB ROM (°)	5.11 (3.90, 7.39)	5.89 (3.86, 11.54)	0.493	0.086[b]
A IR-ER ROM (°)	22.28 (18.61, 27.36)	25.09 (23.45, 33.55)	0.030*	0.272[b]
Hip Joint				
HF (°)	26.23 (13.28, 30.40)	23.53 (14.17, 30.75)	0.851	0.024[b]
HE (°)	-16.58 (-29.81, -13.30)	-21.34 (-30.59, -15.16)	0.227	0.151[b]
HAD (°)	4.94 (2.17, 10.34)	3.06 (1.30, 5.58)	0.083	0.217[b]
HAB (°)	-6.66 (-8.08, -1.97)	-7.35 (-10.42, -4.88)	0.292	0.132[b]
Peak HIR (°)	27.19 (11.51, 31.90)	13.47 (-2.72, 17.94)	0.004**	0.363[b]
HER (°)	-7.13 (-15.84, 2.13)	-15.19 (-31.06, -7.52)	0.035*	0.263[b]
H F-E ROM (°)	43.34 (41.85, 44.83)	44.64 (42.68, 47.42)	0.091	0.212[b]
H AD-AB ROM (°)	11.08 (9.76, 12.85)	10.92 (8.93, 12.76)	0.687	0.050[b]
H IR-ER ROM (°)	29.28 (22.40, 33.37)	26.31 (20.34, 39.69)	0.485	0.087[b]
Knee Joint				
KF (°)	53.24 (48.47, 61.31)	61.88 (53.79, 64.09)	0.024*	0.282[b]
KE (°)	2.10 (-4.86, 4.96)	-2.24 (-6.56, 0.03)	0.013*	0.310[b]
KAD (°)	20.90 (13.73, 25.28)	18.95 (11.95, 23.05)	0.086	0.215[b]
KAB (°)	-3.65 (-6.84, 3.22)	-3.16 (-5.32, 1.80)	0.716	0.045[b]
KIR (°)	-0.76 (-3.82, 13.93)	5.05 (-1.51, 18.75)	0.056	0.239[b]
KER (°)	-18.73 (-21.80, -9.09)	-14.18 (-22.34, -6.41)	0.460	0.092[b]
K F-E ROM (°)	54.82 (46.34, 59.67)	63.78 (60.99, 67.38)	**<0.001*** **	0.646[b]
K AD-AB ROM (°)	20.83 (17.65, 31.62)	19.74 (14.53, 22.69)	0.096	0.208[b]
K IR-ER ROM (°)	20.03 (15.68, 23.38)	21.45 (18.11, 25.07)	0.212	0.156[b]

Note:

Data presented as median (Q1, Q3) unless otherwise noted.

Bonferroni corrected α = 0.002 for 27 comparisons.

***P < 0.001; **P < 0.01; *P < 0.05

ROM: Range of Motion; °: degrees

[a]Effect size reported as Cohen's d; [b]Effect size reported as r value

Abbreviations:

ADF: Ankle dorsiflexion; APF: Ankle plantarflexion; AAD: Ankle adduction; AAB: Ankle abduction; AIR: Ankle internal rotation; AER: Ankle external rotation;

HF: Hip flexion; HE: Hip extension; HAD: Hip adduction; HAB: Hip abduction; HIR: Hip internal rotation; HER: Hip external rotation;

KF: Knee flexion; KE: Knee extension; KAD: Knee adduction; KAB: Knee abduction; KIR: Knee internal rotation; KER: Knee external rotation;

A: Ankle; H: Hip; K: Knee; F-E: Flexion-Extension; AD-AB: Adduction-Abduction; IR-ER: Internal-External Rotation; ROM: Range of Motion

Supplementary Table S31

Comparison of Joint Moments Between Affected and Unaffected Sides During Normal Speed Walking in Lower Functional Outcome Group

Variable	Side		*P*-value	EffectSize
	AffectedSide(n=32)	UnaffectedSide(n=32)		
Ankle Joint				
PAFM (N·mm/kg)	1200.00 (1023.72, 1510.02)	1297.45 (870.62, 1427.76)	0.914	0.013[b]
PAAM (N·mm/kg)	127.55 (64.61, 212.90)	127.81 (71.02, 184.56)	0.851	0.024[b]
PAIRM (N·mm/kg)	198.57 (151.95, 259.41)	223.32 (181.99, 259.30)	0.227	0.151[b]
Hip Joint				
PHFM (N·mm/kg)	789.41 (613.74, 962.88)	696.21 (582.65, 855.20)	0.301	0.129[b]
PHAM (N·mm/kg)	953.23 (773.55, 1232.67)	956.44 (716.65, 1074.34)	0.582	0.069[b]
PHIRM (N·mm/kg)	163.99 (114.21, 249.38)	214.69 (172.82, 263.07)	0.025*	0.280[b]
Knee Joint				
PKFM (N·mm/kg)	494.58 (255.71, 1914.56)	584.25 (307.20, 2373.25)	0.301	0.129[b]
PKAM (N·mm/kg)	544.06 (469.58, 741.71)	583.69 (416.60, 728.83)	0.861	0.022[b]
PKIRM (N·mm/kg)	181.24 (147.78, 234.48)	212.75 (178.27, 251.91)	0.155	0.178[b]

Note:

Data presented as median (Q1, Q3).

Bonferroni corrected α = 0.006 for 9 comparisons.

**P < 0.01; *P < 0.05

Nmm/kg: Newton-millimeters per kilogram

[a]Effect size reported as Cohen's d; [b]Effect size reported as r value

Abbreviations:

PAFM: Peak ankle flexion moment; PAAM: Peak ankle adduction moment; PAIRM: Peak ankle internal rotation moment;

PHFM: Peak hip flexion moment; PHAM: Peak hip adduction moment; PHIRM: Peak hip internal rotation moment;

PKFM: Peak knee flexion moment; PKAM: Peak knee adduction moment; PKIRM: Peak knee internal rotation moment

Dual Task

Supplementary Table S32

Comparison of Joint Flexion/Adduction/Internal Rotation Angles During Dual-Task Walking Between Lower Functional Outcome and Higher Functional Outcome Groups

Variable	Group		*P*-value	EffectSize
	**Lower Functional Outcome(n=32)**	**Higher Functional Outcome(n=32)**		
Affected/Non-dominant Side				
ADF (°)	12.98 (9.20, 16.15)	13.80 (11.38, 17.42)	0.222	0.15[b]
AAD (°)	6.52 (3.52, 9.00)	4.14 (1.79, 6.66)	0.006**	0.35[b]
AIR (°)	2.85 (-5.91, 9.40)	8.58 (-1.31, 20.46)	0.008**	0.33[b]
HF (°)	21.15 (15.14, 25.36)	19.81 (15.72, 24.05)	0.591	0.07[b]
HAD (°)	6.48 (0.41, 9.80)	7.20 (4.72, 9.92)	0.066	0.23[b]
HIR (°)	44.50 (31.67, 52.54)	22.34 (9.91, 35.21)	**<0.001*** **	0.52[b]
KF (°)	38.52 (30.34, 47.00)	52.61 (47.63, 57.23)	**<0.001*** **	0.51[b]
KAD (°)	35.83 (26.52, 45.47)	16.00 (7.90, 21.66)	**<0.001*** **	0.72[b]
KIR (°)	-1.14 (-4.04, 1.36)	7.25 (2.97, 13.58)	**<0.001*** **	0.63[b]
UnAffected/Dominant Side				
ADF (°)	15.70 (12.35, 18.62)	17.09 (14.71, 20.48)	0.093	0.21[b]
AAD (°)	4.44 (3.68, 6.46)	4.50 (-1.23, 6.68)	0.546	0.08[b]
AIR (°)	6.05 (0.22, 27.07)	9.48 (-1.34, 32.10)	0.601	0.07[b]
HF (°)	26.15 (20.39, 32.91)	20.29 (17.03, 24.79)	0.007**	0.34[b]
HAD (°)	6.29 (3.47, 12.44)	5.55 (2.82, 9.53)	0.361	0.11[b]
HIR (°)	26.24 (10.60, 30.33)	28.84 (-8.36, 44.06)	0.452	0.09[b]
KF (°)	59.50 (49.12, 61.70)	50.87 (47.31, 55.81)	0.003**	0.38[b]
KAD (°)	22.41 (17.40, 32.11)	23.08 (4.21, 31.65)	0.405	0.10[b]
KIR (°)	7.11 (3.17, 12.05)	2.20 (-5.20, 3.99)	**<0.001*** **	0.45[b]

Note:

Data presented as median (Q1, Q3) unless otherwise noted.

Bonferroni corrected α = 0.006 for 9 comparisons.

***P < 0.001; **P < 0.01; *P < 0.05

°: degrees

[a]Effect size reported as Cohen's d; [b]Effect size reported as r value

Abbreviations:

ADF: Ankle dorsiflexion; AAD: Ankle adduction; AIR: Ankle internal rotation;

HF: Hip flexion; HAD: Hip adduction; HIR: Hip internal rotation;

KF: Knee flexion; KAD: Knee adduction; KIR: Knee internal rotation

Supplementary Table S33

Comparison of Joint Angles and Range of Motion Between Affected and Unaffected Sides During Balance Task in Lower Functional Outcome Group

**Variable**	**Side**		***P*-value**	**EffectSize**
	**AffectedSide(n=32)**	**UnaffectedSide(n=32)**		
Ankle Joint				
ADF (°)	7.65 (5.74, 17.18)	16.14 (11.55, 19.25)	0.002**	0.39[b]
AAD (°)	5.91 (1.23, 7.18)	5.00 (3.39, 6.64)	0.667	0.05[b]
AIR (°)	-3.16 (-11.51, 24.02)	-0.25 (-5.23, 27.38)	0.277	0.14[b]
APF (°)	-19.39 ± 6.57	-13.08 ± 4.27	**<0.001*** **	1.10[a]
AAB (°)	0.00 (-4.75, 1.52)	-0.47 (-6.48, 0.37)	0.006**	0.35[b]
AER (°)	-34.43 (-37.25, -9.34)	-25.74 (-32.90, -19.38)	0.001**	0.41[b]
A F-E ROM (°)	29.99 (27.39, 32.36)	27.74 (25.13, 31.94)	0.227	0.15[b]
Hip Joint				
HF (°)	24.41 ± 7.49	28.66 ± 9.07	0.052	0.50[a]
HAD (°)	4.58 (2.54, 7.76)	8.37 (4.73, 12.91)	0.011*	0.32[b]
HIR (°)	32.53 (18.66, 49.93)	26.70 (-4.67, 36.13)	0.012*	0.32[b]
HE (°)	-19.01 (-28.35, -15.21)	-14.09 (-25.74, -13.00)	0.001**	0.41[b]
HAB (°)	-6.40 (-7.31, -3.73)	-2.11 (-5.72, 1.95)	**<0.001*** **	0.45[b]
HER (°)	-4.59 (-10.86, 1.44)	-17.78 (-31.65, 3.86)	0.006**	0.35[b]
H F-E ROM (°)	44.11 (42.04, 45.75)	42.84 (40.15, 47.45)	0.383	0.11[b]
Knee Joint				
KF (°)	40.25 (32.99, 53.75)	55.49 ± 4.54	**<0.001*** **	0.48[b]
KAD (°)	27.12 (23.91, 41.21)	24.22 (8.71, 28.38)	0.001**	0.41[b]
KIR (°)	-1.02 (-2.85, 5.08)	11.04 (4.27, 23.70)	**<0.001*** **	0.56[b]
KE (°)	-7.26 (-9.77, -2.26)	2.55 (-7.94, 6.92)	**<0.001*** **	0.56[b]
KAB (°)	-2.19 (-6.33, 2.70)	-3.36 (-5.61, 3.26)	0.023*	0.29[b]
KER (°)	-17.36 (-20.39, -9.76)	-5.06 (-8.23, 10.68)	**<0.001*** **	0.45[b]
K F-E ROM (°)	47.96 (37.90, 65.97)	55.43 (50.53, 62.08)	0.057	0.24[b]

Note:

Data presented as median (Q1, Q3) unless otherwise noted.

Bonferroni corrected α = 0.002 for 21 comparisons.

***P < 0.001; **P < 0.01; *P < 0.05

ROM: Range of Motion; °: degrees

[a]Effect size reported as Cohen's d; [b]Effect size reported as r value

Abbreviations:

ADF: Ankle dorsiflexion; APF: Ankle plantarflexion; AAD: Ankle adduction; AAB: Ankle abduction; AIR: Ankle internal rotation; AER: Ankle external rotation;

HF: Hip flexion; HE: Hip extension; HAD: Hip adduction; HAB: Hip abduction; HIR: Hip internal rotation; HER: Hip external rotation;

KF: Knee flexion; KE: Knee extension; KAD: Knee adduction; KAB: Knee abduction; KIR: Knee internal rotation; KER: Knee external rotation;

A: Ankle; H: Hip; K: Knee; F-E: Flexion-Extension; ROM: Range of Motion

Supplementary Table S34

Comparison of Joint Moments Between Affected and Unaffected Sides During Balance Task in Lower Functional Outcome Group

Variable	Side		*P*-value	EffectSize
	AffectedSide(n=32)	UnaffectedSide(n=32)		
Ankle Joint				
PAFM (N·mm/kg)	1035.97 (744.71, 1339.77)	1143.88 (975.95, 1263.52)	0.283	0.13[b]
PAAM (N·mm/kg)	160.01 (84.16, 268.96)	130.93 (78.45, 473.02)	0.745	0.04[b]
PAIRM (N·mm/kg)	284.12 (179.92, 383.20)	225.71 (178.93, 506.72)	0.908	0.02[b]
Hip Joint				
PHFM (N·mm/kg)	788.55 (484.79, 1051.22)	486.65 (420.79, 737.71)	0.001**	0.42[b]
PHAM (N·mm/kg)	1112.56 ± 652.55	1024.17 ± 596.26	0.604	0.13[a]
PHIRM (N·mm/kg)	244.63 ± 97.09	166.28 ± 63.41	**<0.001*** **	1.04[a]
Knee Joint				
PKFM (N·mm/kg)	460.97 (149.65, 2890.21)	521.69 (405.90, 1882.28)	0.908	0.02[b]
PKAM (N·mm/kg)	489.21 (384.05, 882.59)	489.40 (380.85, 1308.58)	0.537	0.08[b]
PKIRM (N·mm/kg)	243.50 ± 96.13	247.22 (176.34, 416.25)	0.066	0.23[b]

Note:

Data presented as median (Q1, Q3) unless otherwise noted.

Bonferroni corrected α = 0.006 for 9 comparisons.

***P < 0.001; **P < 0.01; *P < 0.05

Nmm/kg: Newton-millimeters per kilogram

[a]Effect size reported as Cohen's d; [b]Effect size reported as r value

Abbreviations:

PAFM: Peak ankle flexion moment; PAAM: Peak ankle adduction moment; PAIRM: Peak ankle internal rotation moment;

PHFM: Peak hip flexion moment; PHAM: Peak hip adduction moment; PHIRM: Peak hip internal rotation moment;

PKFM: Peak knee flexion moment; PKAM: Peak knee adduction moment; PKIRM: Peak knee internal rotation moment

fNIRS

Normal-Speed Task

Supplementary Table S35

Comparison of Oxygenated Hemoglobin Concentrations During Normal-Paced Walking Between Lower Functional Outcome and Higher Functional Outcome

Channel	Groups: Lower Functional Outcome (n=32)	Groups: Higher Functional Outcome (n=32)	*P*-value	Effect Size
PFA (Polar Frontal Area)				
S3-D2	0.021 ± 0.041	-0.001 ± 0.039	**0.029***	0.560[a]
S3-D3	0.013 ± 0.030	0.011 (-0.018, 0.030)	0.421	0.101[b]
S3-D8	0.008 ± 0.023	0.000 ± 0.037	0.314	0.254[a]
S4-D3	0.014 (-0.001, 0.027)	0.012 (-0.016, 0.036)	0.582	0.069[b]
S4-D4	0.006 ± 0.027	0.014 ± 0.050	0.449	0.191[a]
S4-D9	0.000 (-0.017, 0.003)	0.005 ± 0.036	0.485	0.087[b]
S9-D3	0.015 ± 0.035	-0.005 ± 0.043	**0.048***	0.504[a]
DLPFC (Dorsolateral Prefrontal Cortex)				
S5-D4	0.008 ± 0.021	0.009 (-0.024, 0.027)	0.962	0.006[b]
S5-D10	0.008 ± 0.022	-0.001 (-0.014, 0.011)	0.434	0.098[b]
S8-D2	-0.001 ± 0.038	0.007 ± 0.036	0.380	0.221[a]
S8-D7	-0.003 (-0.021, 0.009)	0.006 (-0.018, 0.028)	0.129	0.190[b]
S8-D8	0.006 (-0.012, 0.022)	0.006 (-0.020, 0.029)	0.610	0.064[b]
S9-D8	0.016 (-0.007, 0.027)	-0.002 (-0.021, 0.022)	0.066	0.230[b]
S9-D9	0.008 ± 0.022	0.004 ± 0.030	0.511	0.165[a]
S10-D4	0.005 (-0.003, 0.024)	0.011 ± 0.044	0.936	0.010[b]
S10-D9	0.002 (-0.017, 0.006)	-0.002 (-0.009, 0.024)	0.757	0.039[b]
S10-D10	0.002 (-0.011, 0.008)	0.007 (-0.007, 0.016)	0.058	0.237[b]
IFA (Inferior Frontal Area)				
S2-D2	0.004 ± 0.022	0.007 ± 0.033	0.651	0.114[a]
S2-D7	-0.004 ± 0.016	0.001 ± 0.025	0.352	0.234[a]
SMA (Sensorimotor Area)				
S1-D1	-0.004 ± 0.030	0.008 ± 0.019	0.060	0.479[a]
S1-D6	-0.018 ± 0.029	-0.009 (-0.020, 0.005)	0.159	0.176[b]
S6-D5	-0.014 ± 0.020	0.005 (-0.014, 0.024)	**0.002****	0.387[b]
S6-D11	-0.022 ± 0.028	0.001 (-0.009, 0.017)	**0.001****	0.408[b]
S7-D1	-0.009 ± 0.027	0.002 (-0.009, 0.018)	**0.010***	0.321[b]
S7-D6	-0.014 ± 0.025	-0.001 ± 0.029	0.059	0.480[a]
S7-D12	-0.013 ± 0.033	0.002 (-0.008, 0.026)	**0.016***	0.300[b]
S7-D13	-0.013 ± 0.035	0.003 (-0.012, 0.021)	**0.027***	0.277[b]

Channel	Groups		P-value	Effect Size
	Lower Functional Outcome (n=32)	Higher Functional Outcome (n=32)		
S11-D5	-0.003 ± 0.018	0.001 (-0.009, 0.016)	0.340	0.119[b]
S11-D11	-0.009 ± 0.022	0.005 ± 0.033	**0.042***	0.519[a]
S12-D12	-0.003 (-0.029, 0.019)	0.002 (-0.009, 0.019)	0.468	0.091[b]
S12-D13	-0.010 (-0.022, 0.023)	0.005 (-0.012, 0.017)	0.248	0.144[b]
S13-D11	-0.018 ± 0.032	-0.002 ± 0.033	0.054	0.491[a]
S13-D14	-0.017 (-0.039, 0.001)	-0.001 ± 0.030	**0.022***	0.285[b]
S14-D11	-0.020 ± 0.025	0.002 ± 0.034	**0.003****	0.766[a]
S14-D14	-0.007 (-0.041, 0.004)	0.003 ± 0.026	**0.047***	0.248[b]

Note:

Data are presented as mean ± SD or median (Q1, Q3).

[a]Effect size reported as Cohen's d; [b]Effect size reported as r value

*P < 0.05; **P < 0.01; ***P < 0.001

Statistical analysis: Independent t-test for normally distributed data; Mann-Whitney U test for non-normally distributed data

All HbO_2 values are expressed in mmol/L·mm

Channel assignments based on Brodmann area (BA) coverage determined by Talairach daemon atlas

Bonferroni corrected α = 0.00143 for 35-channel comparisons. Bold values indicate findings significant after correction.

Abbreviations:

HbO_2: Oxygenated hemoglobin; S: Source optode; D: Detector optode; Q1: First quartile; Q3: Third quartile; SD: Standard deviation; BA: Brodmann area

Dual Task

Supplementary Table S36

Comparison of Oxygenated Hemoglobin Concentrations During Dual-Task Performance Between Lower Functional Outcome and Higher Functional Outcome

Channel	Groups		P-value	Effect Size
	Lower Functional Outcome (n=32)	Higher Functional Outcome (n=32)		
PFA (Polar Frontal Area)				
S3-D2	0.023 ± 0.053	0.040 ± 0.055	0.217	0.312[a]
S3-D3	0.003 ± 0.038	0.048 ± 0.057	**0.000*****	0.932[a]

Channel	Groups		P-value	Effect Size
	Lower Functional Outcome (n=32)	Higher Functional Outcome (n=32)		
S3-D8	0.010 ± 0.030	0.022 ± 0.036	0.158	0.358[a]
S4-D3	0.000 (-0.026, 0.041)	0.048 ± 0.068	**0.003****	0.772[b]
S4-D4	0.012 ± 0.051	0.057 ± 0.070	**0.005****	0.731[a]
S4-D9	0.002 ± 0.047	0.031 ± 0.035	**0.006****	0.716[a]
S9-D3	0.010 ± 0.052	0.026 ± 0.065	0.280	0.272[a]
DLPFC (Dorsolateral Prefrontal Cortex)				
S5-D4	-0.006 ± 0.043	0.007 ± 0.045	0.219	0.311[a]
S5-D10	-0.010 (-0.010, 0.016)	0.009 (-0.002, 0.022)	0.191	0.331[b]
S8-D2	0.007 ± 0.071	0.021 ± 0.064	0.401	0.211[a]
S8-D7	0.002 (-0.014, 0.019)	0.021 (-0.029, 0.058)	0.277	0.274[b]
S8-D8	0.001 (-0.025, 0.033)	-0.003 (-0.023, 0.046)	0.802	0.063[b]
S9-D8	0.001 (-0.021, 0.021)	0.007 (-0.017, 0.038)	0.636	0.119[b]
S9-D9	0.028 (-0.009, 0.027)	0.015 (-0.006, 0.025)	0.625	0.123[b]
S10-D4	0.009 ± 0.050	0.043 ± 0.066	**0.022***	0.585[a]
S10-D9	0.012 ± 0.044	0.028 ± 0.032	0.105	0.411[a]
S10-D10	-0.002 (-0.021, 0.019)	0.012 (-0.011, 0.030)	0.084	0.440[b]
IFA (Inferior Frontal Area)				
S2-D2	0.010 ± 0.032	0.011 ± 0.057	0.928	0.023[a]
S2-D7	0.006 ± 0.035	0.017 ± 0.046	0.289	0.267[a]
SMA (Sensorimotor Area)				
S1-D1	0.002 ± 0.026	0.009 ± 0.029	0.356	0.232[a]
S1-D6	-0.013 (-0.014, 0.024)	0.019 ± 0.043	**0.037***	0.534[b]
S6-D5	-0.008 ± 0.031	0.029 ± 0.046	**0.000*****	0.942[a]
S6-D11	0.007 ± 0.051	0.022 ± 0.042	0.201	0.323[a]
S7-D1	0.011 (-0.018, 0.027)	0.014 (-0.010, 0.037)	0.803	0.063[b]
S7-D6	-0.001 ± 0.036	0.012 ± 0.044	0.222	0.308[a]
S7-D12	0.000 (-0.026, 0.050)	0.030 ± 0.031	**0.008****	0.689[b]
S7-D13	0.001 ± 0.046	0.013 ± 0.037	0.242	0.296[a]
S11-D5	0.002 (-0.026, 0.014)	0.007 (-0.002, 0.022)	0.600	0.132[b]
S11-D11	0.004 (-0.018, 0.023)	0.013 (-0.011, 0.033)	0.474	0.180[b]
S12-D12	0.014 (-0.036, 0.059)	0.020 ± 0.042	0.684	0.102[b]

Channel	Groups		P-value	Effect Size
	Lower Functional Outcome (n=32)	Higher Functional Outcome (n=32)		
S12-D13	0.012 (-0.016, 0.039)	0.017 ± 0.027	0.749	0.080[b]
S13-D11	-0.004 ± 0.036	0.030 ± 0.046	**0.001*** **	0.833[a]
S13-D14	0.000 (-0.025, 0.038)	0.017 ± 0.034	0.118	0.396[b]
S14-D11	-0.002 ± 0.036	0.013 ± 0.031	0.100	0.417[a]
S14-D14	-0.019 (-0.035, 0.006)	0.015 ± 0.034	**0.013***	0.636[b]

Note:

Data are presented as mean ± SD or median (Q1, Q3).

[a]Effect size reported as Cohen's d; [b]Effect size reported as r value

*P < 0.05; **P < 0.01; ***P < 0.001

Statistical analysis: Independent t-test for normally distributed data; Mann-Whitney U test for non-normally distributed data

All HbO_2 values are expressed in mmol/L·mm

Channel assignments based on detailed anatomical localization and functional specialization

Bonferroni corrected α = 0.00143 for 35-channel comparisons. Bold values indicate findings significant after correction.

Abbreviations:

HbO_2: Oxygenated hemoglobin; PFC: Prefrontal cortex; S: Source optode; D: Detector optode; Q1: First quartile; Q3: Third quartile; SD: Standard deviation; BA: Brodmann area

Balance Task

Supplementary Table S37

Comparison of Oxygenated Hemoglobin Concentrations During Balance Task Between Lower Functional Outcome and Higher Functional Outcome

Channel	Groups		P-value	Effect Size
	Lower Functional Outcome (n=32)	Higher Functional Outcome (n=32)		
PFA (Polar Frontal Area)				
S3-D2	-0.000 (-0.016, 0.036)	0.041 (-0.005, 0.083)	**0.038***	0.529[a]
S3-D3	0.014 (-0.030, 0.056)	0.032 (0.001, 0.056)	0.163	0.353[a]
S3-D8	-0.004 (-0.050, 0.026)	0.024 (0.003, 0.046)	0.168	0.349[a]
S4-D3	0.002 (-0.025, 0.058)	0.013 (-0.007, 0.050)	0.367	0.227[a]
S4-D4	0.030 (-0.039, 0.063)	0.027 (-0.004, 0.054)	0.358	0.232[a]

Channel	Groups		*P*-value	Effect Size
	Lower Functional Outcome (n=32)	Higher Functional Outcome (n=32)		
S4-D9	0.013 ± 0.038	0.023 ± 0.034	0.265	0.281[a]
S9-D3	-0.016 (-0.041, 0.014)	0.017 (-0.018, 0.045)	0.267	0.280[a]
DLPFC (Dorsolateral Prefrontal Cortex)				
S5-D4	-0.011 (-0.035, 0.019)	0.012 (-0.001, 0.034)	0.533	0.157[a]
S5-D10	0.003 (-0.018, 0.025)	0.007 (-0.007, 0.016)	0.739	0.084[a]
S8-D2	0.001 (-0.033, 0.025)	0.025 (0.002, 0.046)	0.067	0.467[a]
S8-D7	0.006 (-0.032, 0.062)	0.011 (-0.003, 0.034)	0.792	0.066[a]
S8-D8	-0.022 (-0.073, 0.008)	0.025 (0.005, 0.056)	**0.000*** **	1.055[a]
S9-D8	-0.008 (-0.030, 0.025)	0.016 (-0.001, 0.043)	0.055	0.489[a]
S9-D9	-0.009 (-0.027, 0.009)	0.012 (-0.002, 0.043)	**0.001** **	0.853[a]
S10-D4	-0.010 (-0.044, 0.039)	0.021 (0.003, 0.058)	0.105	0.412[a]
S10-D9	0.002 (-0.008, 0.022)	0.007 (-0.001, 0.039)	0.080	0.444[a]
S10-D10	-0.006 (-0.022, 0.016)	0.011 (-0.006, 0.026)	0.148	0.366[a]
IFA (Inferior Frontal Area)				
S2-D2	-0.000 (-0.025, 0.019)	0.007 (-0.008, 0.030)	0.195	0.328[a]
S2-D7	0.003 (-0.019, 0.026)	0.007 (-0.003, 0.024)	0.408	0.208[a]
SMA (Sensorimotor Area)				
S1-D1	-0.004 (-0.023, 0.011)	0.008 (-0.005, 0.026)	0.057	0.486[a]
S1-D6	-0.000 ± 0.040	0.019 ± 0.033	**0.037***	0.534[a]
S6-D5	0.004 (-0.040, 0.026)	0.013 (-0.014, 0.043)	0.198	0.326[a]
S6-D11	0.002 (-0.022, 0.036)	0.015 (-0.005, 0.039)	0.862	0.044[a]
S7-D1	-0.008 ± 0.032	0.016 ± 0.041	**0.011***	0.658[a]
S7-D6	-0.009 (-0.031, 0.017)	0.014 (-0.010, 0.051)	**0.006** **	0.713[a]
S7-D12	0.007 (-0.011, 0.033)	0.028 (-0.004, 0.062)	0.145	0.369[a]
S7-D13	0.005 (-0.020, 0.020)	0.016 (-0.009, 0.045)	0.071	0.459[a]
S11-D5	-0.001 (-0.029, 0.015)	0.003 (-0.011, 0.015)	0.249	0.291[a]
S11-D11	-0.012 (-0.035, 0.028)	0.017 (-0.008, 0.045)	0.103	0.414[a]
S12-D12	0.010 (-0.011, 0.034)	0.024 (-0.000, 0.050)	0.145	0.369[a]
S12-D13	0.005 (-0.024, 0.018)	0.030 (-0.002, 0.052)	**0.022***	0.588[a]
S13-D11	0.004 ± 0.051	0.022 ± 0.057	0.165	0.351[a]
S13-D14	0.008 (-0.011, 0.022)	0.018 (-0.009, 0.045)	0.714	0.092[a]

Channel	Groups		*P*-value	Effect Size
	**Lower Functional Outcome (n=32)**	**Higher Functional Outcome (n=32)**		
S14-D11	-0.007 ± 0.035	0.019 ± 0.038	**0.007****	0.698[a]
S14-D14	-0.006 ± 0.041	0.011 ± 0.042	0.121	0.393[a]

Note:

Data are presented as mean ± SD or median (Q1, Q3) based on Shapiro-Wilk normality test results.

[a]Effect size reported as Cohen's d

*P < 0.05; **P < 0.01; ***P < 0.001

Statistical analysis: Independent t-test for normally distributed data; Mann-Whitney U test for non-normally distributed data

All HbO_2 values are expressed in mmol/L·mm

Regional classification based on precise Brodmann area mapping and functional specialization

Bonferroni corrected α = 0.00143 for 35-channel comparisons. Bold values indicate findings significant after correction.

Abbreviations:

HbO_2: Oxygenated hemoglobin; S: Source optode; D: Detector optode; Q1: First quartile; Q3: Third quartile; SD: Standard deviation; BA: Brodmann area; PFC: Prefrontal cortex

Supplementary Table S38: Network Topology Characteristics Across Walking Conditions

Walking Condition	Group	Total Connections	Connection Strength			Network Density	Mean GC ± SD	Primary Driver (Net Flow)
			**Strong**	**Medium**	**Weak**			
**Normal-Paced Walking**	Lower Functional Outcome (n=32)	5	0	5	0	0.313	0.172 ± 0.016	**IFA (0.213)**
	Higher Functional Outcome (n=32)	2	0	2	0	0.125	0.152 ± 0.003	IFA (0.079)
**Dual-Task Walking**	Lower Functional Outcome (n=32)	**8**	**7**	1	0	0.500	**0.212 ± 0.024**	**SMA (+0.100)**
	Higher Functional Outcome	6	1	5	0	0.375	0.176 ± 0.027	**PFA (+0.050)**

Walking Condition	Group	Total Connections	Connection Strength			Network Density	Mean GC ± SD	Primary Driver (Net Flow)
			Strong	Medium	Weak			
	(n=32)							
**Balance-Task Walking**	Lower Functional Outcome (n=32)	4	0	4	0	0.250	0.173 ± 0.014	**IFA (+0.162)**
	Higher Functional Outcome (n=32)	2	0	2	0	0.125	0.164 ± 0.010	IFA (+0.086)

Note:

Network density = total connections / maximum possible connections (16). Connection strength classification: Strong (GC > 0.20), Medium (GC 0.15-0.20), Weak (GC < 0.15). All GC values are dimensionless. Bold values indicate most prominent features.

Supplementary Table S39: Significant Granger Causality Connections During Different Walking Conditions

Connection	Normal-Paced Walking		Dual-Task Walking		Balance-Task Walking	
	GC Value	Strength	GC Value	Strength	GC Value	Strength
**Lower Functional Outcome (n=32)**						
IFA → PFA	0.155	M	**0.203**	**S**	0.187	M
IFA → SMA	--	--	--	--	0.174	M
IFA → DLPFC	0.186	M	--	--	--	--
SMA → PFA	0.192	M	**0.233**	**S**	--	--
SMA → DLPFC	0.158	M	**0.252**	**S**	--	--
PFA → IFA	--	--	**0.219**	**S**	--	--
PFA → SMA	0.167	M	0.173	M	0.176	M
PFA → DLPFC	--	--	**0.224**	**S**	--	--
DLPFC → SMA	--	--	**0.215**	**S**	0.153	M
DLPFC → PFA	--	--	**0.205**	**S**	--	--
**Higher Functional Outcome (n=32)**						
SMA → PFA	0.150	M	0.153	M	0.172	M
SMA → DLPFC	--	--	0.182	M	--	--
PFA → SMA	0.155	M	0.156	M	0.157	M
PFA → DLPFC	--	--	**0.226**	**S**	--	--
DLPFC → SMA	--	--	0.161	M	--	--
DLPFC → PFA	--	--	0.178	M	--	--

Note:

GC values are dimensionless; Freq = frequency of occurrence across subjects (%); Bold and highlighted values indicate strong connections (GC > 0.20); (--) indicates non-significant connection below threshold criteria. Strong connections are highlighted in yellow.

Supplementary Table S40: Regional Net Flow Values and Hierarchical Rankings

Brain Region	Normal-Paced Walking		Dual-Task Walking		Balance-Task Walking	
	Lower Functional Outcome	Higher Functional Outcome	Lower Functional Outcome	Higher Functional Outcome	Lower Functional Outcome	Higher Functional Outcome
IFA	+0.213 (R1)	+0.079 (R1)	-0.010 (R2)	+0.046 (R2)	+0.162 (R1)	+0.086 (R1)
SMA	-0.005 (R2)	-0.029 (R3)	+0.100 (R1)	-0.012 (R3)	-0.141 (R4)	+0.006 (R2)
PFA	-0.088 (R3)	-0.017 (R2)	-0.025 (R3)	+0.050 (R1)	-0.055 (R3)	-0.046 (R3)
DLPFC	-0.120 (R4)	-0.033 (R4)	-0.064 (R4)	-0.083 (R4)	+0.034 (R2)	-0.047 (R4)

Note:

Values represent net flow (sum of outgoing minus incoming causal influences). R1-R4 indicate hierarchical ranking within each condition and group (R1 = primary driver, R4 = primary receiver). Bold values indicate strongest drivers/receivers within each condition. Highlighted values indicate most prominent changes from normal walking.

Abbreviations:

IFA: inferior frontal area; SMA: sensorimotor area; PFA: polar frontal area; DLPFC: dorsolateral prefrontal cortex; R: rank; GC: Granger causality

EMG

Supplementary Table S41

Comparison of EMG Activity During Normal Speed Walking Between Lower Functional Outcome and Higher Functional Outcome Groups(percentage)

Muscle	Group		P-value	EffectSize
	Lower Functional Outcome(n=32)	Higher Functional Outcome(n=32)		
Affected Side				
Quadriceps	10.91 (9.15, 13.43)	5.22 (4.16, 9.69)	**<0.001*****	0.53[b]
Semitendinosus	25.65 ± 11.50	12.56 ± 5.54	**<0.001*****	1.45[a]
Biceps Femoris	21.04 (18.02, 26.07)	19.69 (7.40, 29.65)	0.340	0.12[b]
Tibialis Anterior	13.17 (10.64, 60.59)	21.66 (13.57, 47.00)	0.175	0.17[b]
Lateral Gastrocnemius	29.54 (23.22, 36.07)	18.10 (13.57, 31.72)	**0.001****	0.40[b]
Unaffected Side				
Quadriceps	20.92 (10.11, 34.96)	11.26 (7.21, 16.38)	**0.004****	0.36[b]
Semitendinosus	24.90 (20.03, 32.38)	12.62 (3.25, 16.11)	**<0.001*****	0.59[b]
Biceps Femoris	15.91 (11.98, 19.27)	9.57 (5.13, 22.13)	0.034*	0.27[b]
Tibialis Anterior	15.97 (7.84, 34.86)	22.25 (13.87, 44.37)	0.022*	0.29[b]
Lateral Gastrocnemius	24.97 (21.50, 28.62)	19.41 (8.80, 33.88)	0.254	0.14[b]

Note:

Data presented as median (Q1, Q3) unless otherwise noted.

***P < 0.001 (significant after Bonferroni correction); **P < 0.01; *P < 0.05

[a]Effect size reported as Cohen's d; [b]Effect size reported as r value

Bonferroni corrected α = 0.005 for between-group comparisons

Abbreviations:

EMG: Electromyography

Supplementary Table S42

Comparison of EMG Activity Between Affected and Unaffected Sides During Normal Speed Walking in Lower Functional Outcome Group

Muscle	Side		P-value	EffectSize
	AffectedSide(n=32)	UnaffectedSide(n=32)		
Quadriceps	10.91 (9.15, 13.43)	20.92 (10.11, 34.96)	**<0.001*****	0.61[b]
Semitendinosus	25.65 ± 11.50	26.09 ± 12.25	0.882	0.04[a]
Biceps Femoris	21.04 (18.02, 26.07)	15.91 (11.98, 19.27)	**0.001****	0.42[b]
Tibialis Anterior	13.17 (10.64, 60.59)	15.97 (7.84, 34.86)	0.028*	0.32[b]
Lateral Gastrocnemius	30.29 ± 8.71	25.51 ± 4.95	**0.009****	0.68[a]

Note:

Data presented as median (Q1, Q3) unless otherwise noted.

***P < 0.001; **P < 0.01 (significant after Bonferroni correction); *P < 0.05

[a]Effect size reported as Cohen's d; [b]Effect size reported as r value

Bonferroni corrected α = 0.01 for within-group comparisons

Supplementary Table S43

Comparison of EMG Activity During Dual Task Between Lower Functional Outcome and Higher Functional Outcome Groups

Muscle	Group		*P*-value	EffectSize
	**Lower Functional Outcome(n=32)**	**Higher Functional Outcome(n=32)**		
Affected Side				
Quadriceps	10.08 (8.41, 16.73)	5.29 (4.47, 12.30)	**0.001****	0.42[b]
Semitendinosus	19.68 (10.04, 25.41)	10.45 (7.80, 14.53)	**<0.001*****	0.46[b]
Biceps Femoris	23.44 (16.02, 26.79)	13.50 (9.08, 20.92)	**<0.001*****	0.46[b]
Tibialis Anterior	11.94 (8.89, 37.17)	21.92 (16.13, 31.21)	0.019*	0.29[b]
Lateral Gastrocnemius	27.87 (21.34, 33.54)	13.91 (8.50, 22.95)	**<0.001*****	0.55[b]
Unaffected Side				
Quadriceps	11.92 (5.62, 28.00)	11.48 (7.81, 16.22)	0.707	0.05[b]
Semitendinosus	31.14 (26.06, 41.67)	11.44 (2.31, 18.39)	**<0.001*****	0.65[b]
Biceps Femoris	15.78 (11.84, 22.25)	6.07 (4.12, 18.08)	**0.001****	0.41[b]
Tibialis Anterior	10.54 (2.70, 38.30)	24.83 (14.84, 27.07)	0.016*	0.30[b]
Lateral Gastrocnemius	26.76 (24.18, 30.77)	11.18 (6.29, 22.22)	**<0.001*****	0.56[b]

Note:

Data presented as median (Q1, Q3) unless otherwise noted.

***P < 0.001 (significant after Bonferroni correction); **P < 0.01; *P < 0.05

[a]Effect size reported as Cohen's d; [b]Effect size reported as r value

Bonferroni corrected α = 0.005 for between-group comparisons

Abbreviations:

EMG: Electromyography

Supplementary Table S44

Comparison of EMG Activity Between Affected and Unaffected Sides During Dual Task in Lower Functional Outcome Group

Muscle	Side		*P*-value	EffectSize
	**AffectedSide(n=32)**	**UnaffectedSide(n=32)**		
Quadriceps	10.08 (8.41, 16.73)	11.92 (5.62, 28.00)	0.727	0.04[b]
Semitendinosus	19.68 (10.04, 25.41)	31.14 (26.06, 41.67)	**0.004****	0.36[b]
Biceps Femoris	22.19 ± 6.95	16.03 ± 7.02	**0.001****	0.88[a]
Tibialis Anterior	11.94 (8.89, 37.17)	10.54 (2.70, 38.30)	0.151	0.18[b]
Lateral Gastrocnemius	27.87 (21.34, 33.54)	26.76 (24.18, 30.77)	0.511	0.08[b]

Note:

Data presented as median (Q1, Q3) unless otherwise noted.

***P < 0.001; **P < 0.01 (significant after Bonferroni correction); *P < 0.05

[a]Effect size reported as Cohen's d; [b]Effect size reported as r value

Bonferroni corrected α = 0.01 for within-group comparisons

Supplementary Table S45

Comparison of EMG Activity During Balance Task Between Lower Functional Outcome and Higher Functional Outcome Groups

Muscle	Group		*P*-value	EffectSize
	Lower Functional Outcome(n=32)	Higher Functional Outcome(n=32)		
Affected Side				
Quadriceps	7.93 (6.09, 11.67)	10.78 (4.02, 16.05)	0.619	0.06[b]
Semitendinosus	29.51 (19.11, 41.18)	17.05 (9.59, 56.62)	0.060	0.24[b]
Biceps Femoris	41.51 (35.62, 48.04)	30.33 (12.70, 38.50)	**<0.001*** **	0.47[b]
Tibialis Anterior	19.49 (15.03, 53.14)	26.89 (16.45, 42.02)	0.591	0.07[b]
Lateral Gastrocnemius	35.70 (26.01, 44.08)	23.65 (14.03, 41.46)	0.032*	0.27[b]
Unaffected Side				
Quadriceps	17.47 (12.55, 21.19)	17.82 (5.54, 28.87)	0.528	0.08[b]
Semitendinosus	28.33 (21.50, 33.02)	13.60 (2.54, 25.33)	**<0.001*** **	0.44[b]
Biceps Femoris	19.00 (14.83, 27.53)	19.23 (5.01, 52.45)	0.747	0.04[b]
Tibialis Anterior	18.70 (9.57, 40.97)	21.57 (16.35, 30.07)	0.184	0.17[b]
Lateral Gastrocnemius	33.10 (29.83, 50.82)	11.71 (7.13, 33.07)	**<0.001*** **	0.54[b]

Note:

Data presented as median (Q1, Q3) unless otherwise noted.

***P < 0.001 (significant after Bonferroni correction); **P < 0.01; *P < 0.05

[a]Effect size reported as Cohen's d; [b]Effect size reported as r value

Bonferroni corrected α = 0.005 for between-group comparisons

Abbreviations:

EMG: Electromyography

Supplementary Table S46

Comparison of EMG Activity Between Affected and Unaffected Sides During Balance Task in Lower Functional Outcome Group

Muscle	Side		*P*-value	EffectSize
	AffectedSide(n=32)	UnaffectedSide(n=32)		
Quadriceps	7.93 (6.09, 11.67)	17.47 (12.55, 21.19)	**<0.001*** **	0.66[b]
Semitendinosus	30.84 ± 13.64	26.63 ± 8.03	0.138	0.38[a]
Biceps Femoris	41.51 (35.62, 48.04)	19.00 (14.83, 27.53)	**<0.001*** **	0.62[b]
Tibialis Anterior	19.49 (15.03, 53.14)	18.70 (9.57, 40.97)	0.212	0.16[b]
Lateral Gastrocnemius	35.70 (26.01, 44.08)	33.10 (29.83, 50.82)	0.151	0.18[b]

Note:

Data presented as median (Q1, Q3) unless otherwise noted.

***P < 0.001 (significant after Bonferroni correction); **P < 0.01; *P < 0.05

[a]Effect size reported as Cohen's d; [b]Effect size reported as r value

Bonferroni corrected α = 0.01 for within-group comparisons

Co-contraction

Supplementary Table S47 Affected Side

Task/Muscle Co-contraction	Group		*P*-value	EffectSize
	Lower Functional Outcome(n=32)	Higher Functional Outcome(n=32)		
Normal Walking Task				
RF-Ham Co-contraction	0.657 ± 0.161	0.612 ± 0.209	0.340	0.24[a]
TA-GL Co-contraction	0.591 ± 0.119	0.747 ± 0.136	**<0.001*** **	1.23[a]
Dual Task				
RF-Ham Co-contraction	0.699 ± 0.161	0.701 ± 0.137	0.967	0.01[a]
TA-GL Co-contraction	0.590 ± 0.176	0.654 ± 0.146	0.120	0.39[a]
Balance Task				
RF-Ham Co-contraction	0.402 ± 0.174	0.544 ± 0.144	**0.001** **	0.89[a]
TA-GL Co-contraction	0.628 ± 0.139	0.742 ± 0.179	**0.006** **	0.71[a]

Note:

Data presented as mean ± standard deviation.

Effect size: ^a^ Cohen's d.

***P < 0.001; **P < 0.01; *P < 0.05

Co-contraction values are normalized ratios.

Bonferroni corrected α = 0.0125 for four co-contraction comparisons per task condition.

Supplementary Table S48 Unaffected Side

Task/MuscleCo-contraction	Group		*P*-value	EffectSize
	Lower Functional Outcome(n=32)	Higher Functional Outcome(n=32)		
Normal Walking Task				
RF-Ham Co-contraction	0.724 ± 0.213	0.787 ± 0.170	0.199	0.33[a]
TA-GL Co-contraction	0.592 ± 0.207	0.581 ± 0.198	0.823	0.06[a]
Dual Task				
RF-Ham Co-contraction	0.578 ± 0.202	0.709 ± 0.148	**0.004****	0.74[a]
TA-GL Co-contraction	0.497 ± 0.265	0.582 ± 0.198	0.148	0.37[a]
Balance Task				
RF-Ham Co-contraction	0.750 ± 0.155	0.830 ± 0.131	**0.030***	0.56[a]
TA-GL Co-contraction	0.594 ± 0.249	0.588 ± 0.154	0.920	0.03[a]

Note:

Data presented as mean ± standard deviation.

Effect size: ^a^ Cohen's d.

***P < 0.001; **P < 0.01; *P < 0.05

Co-contraction values are normalized ratios.

Bonferroni corrected α = 0.0125 for four co-contraction comparisons per task condition.

Abbreviations:

RF = Rectus Femoris; Ham = Hamstring muscles;

TA = Tibialis Anterior; GL = Gastrocnemius Lateralis

RF-Ham = Rectus Femoris and Hamstring Co-contraction

TA-GL = Tibialis Anterior and Gastrocnemius Lateralis Co-contraction

Supplementary Table S49

Correlation Analysis Results Between Brain Oxygenation Changes and Muscle Co-contraction by Task

Channel	Brain Region	Muscle	Side	r / ρ	*P*-Value	Method
Balance-Challenged Walking — Lower Functional Outcome (11 correlations)						
S6-D5	SMC (BA 1/2/3)	RF-Ham	Unaffected	0.487	**0.0047****	Spearman
S11-D5	PMC (BA 6)	RF-Ham	Unaffected	0.466	**0.0071****	Spearman
S3-D3	PFA (BA 10)	RF-Ham	Affected	-0.460	**0.0080****	Spearman
S13-D14	PMC (BA 6)	RF-Ham	Unaffected	0.440	**0.0118***	Spearman
S7-D1	SMA (BA 6)	TA-GL	Affected	0.431	**0.0138***	Spearman
S10-D9	DLPFC (BA 46)	RF-Ham	Affected	-0.420	**0.0167***	Spearman
S7-D6	SMA (BA 6)	RF-Ham	Unaffected	0.412	**0.0192***	Spearman
S4-D9	PFA (BA 10)	RF-Ham	Affected	-0.400	**0.0232***	Spearman
S13-D11	PMC (BA 6)	RF-Ham	Unaffected	0.391	**0.0269***	Spearman
S4-D3	PFA (BA 10)	RF-Ham	Affected	-0.357	**0.0451***	Spearman
S11-D11	PMC (BA 6)	RF-Ham	Affected	-0.356	**0.0458***	Spearman
Balance-Challenged Walking — Higher Functional Outcome (3 correlations)						
S9-D8	DLPFC (BA 46)	TA-GL	Affected	0.442	**0.0112***	Spearman
S8-D2	DLPFC (BA 9)	TA-GL	Affected	0.393	**0.0259***	Spearman
S1-D1	PFA (BA 10)	RF-Ham	Affected	-0.353	**0.0475***	Pearson
Normal Walking — Lower Functional Outcome (12 correlations)						
S2-D7	IFA (BA 45)	TA-GL	Unaffected	-0.504	**0.0033****	Spearman
S6-D5	SMC (BA 1/2/3)	RF-Ham	Unaffected	0.473	**0.0063****	Spearman
S11-D5	PMC (BA 6)	TA-GL	Affected	0.473	**0.0063****	Pearson
S9-D9	DLPFC (BA 9/46)	RF-Ham	Affected	0.446	**0.0105***	Pearson
S6-D5	SMC (BA 1/2/3)	TA-GL	Affected	0.432	**0.0135***	Pearson
S7-D1	SMA (BA 6)	RF-Ham	Unaffected	0.410	**0.0197***	Spearman
S3-D3	PFA (BA 10)	TA-GL	Affected	0.395	**0.0253***	Pearson
S7-D12	SMA (BA 6/4)	RF-Ham	Unaffected	0.387	**0.0286***	Spearman
S1-D6	SMC (BA 6)	RF-Ham	Unaffected	0.384	**0.0302***	Spearman
S12-D13	SMA (BA 6)	RF-Ham	Unaffected	0.362	**0.0418***	Spearman
S14-D11	PMC (BA 6)	RF-Ham	Unaffected	0.358	**0.0445***	Spearman
S10-D10	DLPFC (BA 9/46)	TA-GL	Affected	0.356	**0.0455***	Spearman
Normal Walking — Higher Functional Outcome (9 correlations)						
S14-D11	PMC (BA 6)	RF-Ham	Unaffected	0.504	**0.0032****	Spearman
S7-D12	SMA (BA 6/4)	TA-GL	Unaffected	-0.463	**0.0076****	Pearson
S5-D10	PFA (BA 10)	TA-GL	Affected	-0.457	**0.0085****	Spearman
S9-D9	DLPFC (BA 9/46)	TA-GL	Affected	-0.438	**0.0122***	Pearson
S2-D7	IFA (BA 45)	RF-Ham	Unaffected	0.434	**0.0131***	Spearman
S7-D13	SMA (BA 6)	RF-Ham	Unaffected	0.400	**0.0235***	Spearman
S4-D3	PFA (BA 10)	TA-GL	Affected	-0.379	**0.0322***	Spearman
S12-D12	SMA (BA 6)	TA-GL	Unaffected	-0.364	**0.0408***	Spearman
S4-D4	PFA (BA 10)	TA-GL	Affected	-0.363	**0.0413***	Pearson
Dual-Task Walking — Lower Functional Outcome (3 correlations)						
S11-D11	PMC (BA 6)	RF-Ham	Affected	0.460	**0.0081****	Spearman

Channel	Brain Region	Muscle	Side	r / ρ	*P*-Value	Method
S10-D10	DLPFC (BA 9/46)	TA-GL	Unaffected	0.382	**0.0308***	Spearman
S11-D5	PMC (BA 6)	RF-Ham	Affected	0.363	**0.0412***	Spearman
Dual-Task Walking — Higher Functional Outcome (12 correlations)						
S3-D3	PFA (BA 10)	TA-GL	Affected	-0.494	**0.0041****	Pearson
S3-D3	PFA (BA 10)	RF-Ham	Unaffected	-0.475	**0.0061****	Pearson
S4-D3	PFA (BA 10)	TA-GL	Affected	-0.439	**0.0120***	Spearman
S13-D14	PMC (BA 6)	RF-Ham	Unaffected	-0.424	**0.0157***	Pearson
S9-D3	DLPFC (BA 46)	TA-GL	Unaffected	0.405	**0.0215***	Spearman
S11-D11	PMC (BA 6)	RF-Ham	Unaffected	-0.397	**0.0245***	Pearson
S4-D3	PFA (BA 10)	RF-Ham	Unaffected	-0.390	**0.0274***	Spearman
S14-D11	PMC (BA 6)	RF-Ham	Unaffected	-0.386	**0.0292***	Pearson
S8-D8	DLPFC (BA 9/46)	TA-GL	Unaffected	0.385	**0.0294***	Spearman
S6-D11	SMC (BA 3/1/6)	RF-Ham	Unaffected	-0.380	**0.0320***	Spearman
S9-D9	DLPFC (BA 9/46)	TA-GL	Affected	0.367	**0.0390***	Spearman
S10-D9	DLPFC (BA 46)	TA-GL	Affected	0.367	**0.0390***	Pearson

Note: All correlations significant at P < 0.05. Method determined by Shapiro-Wilk normality test.

Abbreviations: DLPFC: dorsolateral prefrontal cortex; IFA: inferior frontal area; PFA: polar frontal area; PMC: premotor cortex; SMA: supplementary motor area; SMC: sensorimotor cortex; RF-Ham: rectus femoris-hamstring; TA-GL: tibialis anterior-gastrocnemius lateralis

Abbreviations:

BA: Brodmann area; DLPFC: dorsolateral prefrontal cortex; fNIRS: functional near-infrared spectroscopy; HbO_2: oxygenated hemoglobin; IFA: inferior frontal area; PFA: polar frontal area; SMA: sensorimotor area

Affected: surgically treated/ipsilateral limb; Unaffected: non-affected/contralateral limb

RF-Ham: rectus femoris-hamstring co-contraction index; TA-GL: tibialis anterior-gastrocnemius lateralis indexco-contraction

Supplementary Table S50

Complete KOOS Subscale Comparison Between Higher and Lower Functional Outcome Groups

KOOS Subscale (0–100)	LFO (n = 32)	HFO (n = 32)	P-value	Effect Size	Test Method
Pain	75.00 (72.22, 80.56)	77.78 (75.00, 80.56)	.199	r = 0.16	Mann–Whitney U
**Symptoms**	**67.41 ± 5.42**	**78.91 ± 5.83**	**<.001*****	**d = 2.04**	**Independent t-test**
**ADL**	**61.31 ± 5.09**	**83.32 ± 2.47**	**<.001*****	**r = 0.86**	**Mann–Whitney U**

**Sport/Rec**	**35.00 (30.00, 40.00)**	**65.00 (60.00, 75.00)**	**<.001*** **	**r = 0.86**	**Mann–Whitney U**
**QOL**	**37.50 (31.25, 43.75)**	**68.75 (62.50, 68.75)**	**<.001*** **	**r = 0.85**	**Mann–Whitney U**

Notes: Data are presented as mean ± SD for normally distributed data or median (Q1, Q3) for non-normally distributed data. Normality assessed using Shapiro–Wilk test. Higher scores indicate better outcomes (100 = no symptoms/full function). Bold values indicate findings significant at P < .05. ***P < .001.

The complete KOOS questionnaire (42 items across five subscales) was administered at enrollment. The ADL subscale was selected as the primary stratification variable for this study given its direct relevance to daily functional capacity. Pain scores did not differ between groups, indicating that persistent pain is unlikely to be the primary driver of functional outcome heterogeneity. The Symptoms difference was primarily driven by self-reported range-of-motion limitations (items S4 and S5, P < .001 each) rather than inflammatory symptoms (Supplementary Table S51).

Abbreviations: KOOS, Knee Injury and Osteoarthritis Outcome Score; LFO, Lower Functional Outcome (KOOS-ADL ≤ 70); HFO, Higher Functional Outcome (KOOS-ADL ≥ 80); ADL, Activities of Daily Living; QOL, Quality of Life.

Supplementary Table S51

KOOS Symptoms Subscale Item-Level Comparison Between Groups

Symptoms Item (0–4 scale)	LFO (n = 32)	HFO (n = 32)	P-value
Inflammatory Symptoms			
S1 – Swelling	0.97 ± 0.78	0.75 ± 0.72	.261
S2 – Grinding/clicking	0.91 ± 0.73	0.75 ± 0.72	.391
S3 – Catching/locking	1.22 ± 0.75	1.00 ± 0.76	.291
S6 – Morning stiffness	1.09 ± 0.73	0.81 ± 0.74	.132
Range-of-Motion Limitations			
**S4 – Difficulty straightening knee fully**	**1.59 ± 0.80**	**0.53 ± 0.57**	**<.001*** **
**S5 – Difficulty bending knee fully**	**1.72 ± 0.63**	**0.91 ± 0.73**	**<.001*** **
Stiffness After Rest			
**S7 – Stiffness after sitting/lying/resting**	**1.62 ± 0.55**	**1.16 ± 0.77**	**.016***

Notes: Data are presented as mean ± SD (0–4 scale; 0 = no problem, 4 = extreme problem). Because item-level responses are ordinal, between-group comparisons used the Mann–Whitney U test. Bold values indicate P < .05. *P < .05; ***P < .001.

The between-group difference in the KOOS Symptoms subscale was primarily driven by range-of-motion limitations (items S4 and S5), not by inflammatory symptoms (S1–S3, S6), supporting the interpretation that functional deficits are related to neuromuscular control rather than ongoing inflammatory processes. Stiffness after prolonged rest (S7) was categorized separately from inflammatory symptoms because it primarily reflects functional range-of-motion limitations rather than ongoing tissue inflammation, consistent with the absence of differences in the inflammatory items (S1, S2, S3, S6).

Abbreviations: LFO, Lower Functional Outcome (KOOS-ADL ≤ 70); HFO, Higher Functional Outcome (KOOS-

ADL ≥ 80); KOOS, Knee Injury and Osteoarthritis Outcome Score.

Supplementary Table S52

Per-figure and per-table statistical details underlying the main-text tables and figures: test name, exact P value, available test statistic (t, U, |Z|, χ², r, or ρ), degrees of freedom where applicable, effect size with 95% confidence interval, and number of biological units per comparison (Manuscript ISCIENCE-D-26-03085).

Sources and notes

- Summary values are aligned to the revised manuscript tables and figure legends.
- Where exact test-statistic fields were available from the working analysis records they are reported directly; where they were not retained in the deposited files, the worksheet reports the exact P value, effect size, confidence interval (if available), and sample size.
- Group 1 = Lower Functional Outcome (LFO, n = 32); Group 2 = Higher Functional Outcome (HFO, n = 32). Means and SDs are formatted as mean ± SD.
- Figure 3 and Figure 4 legend statistics are provided in dedicated sections below for editorial cross-checking.
- Multiple-comparison (Bonferroni) thresholds: biomechanics α = .0125 (normal & dual-task) / .007 (balance-challenged); sEMG co-contraction α = .006; 35-channel fNIRS α = .00143.
- Brain–muscle coupling (Table 5 / Figure 4) is exploratory, reported at uncorrected P < .05; no comparison survived FDR (Benjamini–Hochberg, q < .05) across 140 channel × muscle pairs per condition.
- Number of biological units per group: n = 32; total N = 64.
- All values shown are direct SPSS output (precision = 3 decimals or as available).

Statistical details for manuscript Table 1 — Baseline demographic and clinical characteristics

Variable	LFO (n=32)	HFO (n=32)	Test	Test statistic	df	Exact P	Effect size (95% CI)	Note
**Demographics — values transcribed from manuscript Table 1**								
Age (years)	49.2 ± 9.4	48.3 ± 8.7	Independent t-test	t = 0.398	62	0.686	Cohen's d = 0.099 (−0.391, 0.589)	From manuscript
Sex (male), n (%)	19 (59.4%)	20 (62.5%)	Pearson χ²	χ² = 0.067	1	0.795	φ = 0.032	From manuscript
Height (m)	1.67 ± 0.09	1.68 ± 0.08	Independent t-test	t = -0.470	62	0.621	Cohen's d = -0.117 (-0.608, 0.373)	Computed from group means ± SDs (manuscript)
Weight (kg)	64.8 ± 7.9	65.4 ± 8.2	Independent t-test	t = -0.298	62	0.763	Cohen's d = -0.075 (-0.565, 0.416)	Computed from group means ± SDs (manuscript)
BMI (kg/m²)	23.4 ± 1.6	23.2 ± 1.8	Independent t-test	t = 0.470	62	0.642	Cohen's d = 0.117 (-0.373, 0.608)	Computed from group means ± SDs (manuscript)
**Clinical Characteristics**								
Affected side (left), n (%)	16 (50.0%)	15 (46.9%)	Pearson χ²	χ² = 0.063	1	0.823	φ = 0.031	From manuscript
Time since surgery (months)	12.0 ± 0.5	12.1 ± 0.4	Independent t-test	t = -0.883	62	0.371	Cohen's d = -0.221 (-0.712, 0.271)	Computed from group means ± SDs (manuscript)
MTPA (°)	87.40 ± 2.66	87.30 ± 2.38	Independent t-test	t = 0.158	62	0.866	Cohen's d = 0.042	Computed from MTPA_radiographic_angle.xlsx
**Functional Outcome (stratification variable)**								
KOOS Pain score (0–100)	75.00 (72.22, 80.56)	77.78 (75.00, 80.56)	Mann–Whitney U	U = 412	—	0.199	r = 0.163	From manuscript
KOOS-ADL score (0–100)	61.31 ± 5.09	83.32 ± 2.47	Independent t-test (Welch)	t = 22.03	44.7	< .001	Cohen's d = 5.51 (4.50, 6.50)	Stratification variable: groups were defined by KOOS-ADL (>=80 vs <=70); the between-group difference is by design and the effect size is not independently interpretable.

Statistical details for manuscript Table 2 — Key biomechanical parameters across task conditions

NOTE: Test statistics are now populated. t values were recalculated from the manuscript Table 2 summary statistics (n=32 per group); Mann–Whitney entries report U where recoverable from the uploaded raw gait data and/or standardized |Z| from the reported P value/effect size. P values, effect sizes, correction thresholds, and sample sizes remain aligned to manuscript Table 2.

Variable	Condition	LFO (n=32)	HFO (n=32)	Test	Test statistic (t, U or |Z|)	df	Exact P	Effect size (95% CI)	Bonferroni α	Significant after correction	Source
**Primary outcome — Knee Coronal Plane ROM Asymmetry (%)**											
Knee coronal plane	Normal walking	14.12 (11.47, 25.54)	6.16 (2.67, 8.37)	Mann–Whitney U	U = 62.0; |Z| = 6.04	—	< .001	r = 0.76 (large)	0.0125	Yes	Manuscript Table 2 + uploaded raw gait dataset
Knee coronal plane	Dual-task walking	32.83 ± 20.74	7.77 ± 4.83	Independent t-test	t = 6.66	62	< .001	Cohen's d = 1.66 (large)	0.0125	Yes	Recalculated from Manuscript Table 2 summaries
Knee coronal plane	Balance-challenged walking	32.74 ± 13.09	7.29 ± 5.42	Independent t-test	t = 10.16	62	< .001	Cohen's d = 2.54 (large)	0.007	Yes	Recalculated from Manuscript Table 2 summaries
Knee transverse plane	Normal walking	18.34 (11.28, 35.76)	40.83 (25.08, 56.83)	Mann–Whitney U	U = 320.0; |Z| = 2.57	—	0.011	r = 0.32	0.0125	Yes	Manuscript Table 2 + uploaded raw gait dataset
Knee transverse plane	Dual-task walking	15.99 (7.77, 32.54)	26.49 (16.98, 43.73)	Mann–Whitney U	|Z| = 2.170	—	0.030	r = 0.28	0.0125	No	Manuscript Table 2 + uploaded raw gait dataset/P value
Knee transverse plane	Balance-challenged walking	21.03 ± 14.65	25.12 ± 18.76	Independent t-test	t = -0.97	62	0.337	Cohen's d = 0.24	0.007	No	Recalculated from Manuscript Table 2 summaries
**Secondary outcome — Affected-Side Joint Power (W/kg)**											
Ankle joint power	Normal walking	2.80 (2.26, 3.09)	3.03 (2.65, 3.26)	Mann–Whitney U	|Z| = 1.598	—	0.110	r = 0.20	0.0125	No	Manuscript Table 2 P value/effect size
Ankle joint power	Dual-task walking	1.06 (0.88, 2.48)	3.07 (2.53, 3.60)	Mann–Whitney U	|Z| = 4.56	—	< .001	r = 0.57 (large)	0.0125	Yes	Manuscript Table 2 P value/effect size
Ankle joint power	Balance-challenged walking	2.81 (2.53, 3.10)	3.22 (2.73, 3.93)	Mann–Whitney U	|Z| = 1.977	—	0.048	r = 0.25	0.007	No	Manuscript Table 2 P value/effect size
Knee joint power	Normal walking	0.50 (0.33, 0.69)	0.65 (0.53, 1.31)	Mann–Whitney U	|Z| = 2.543	—	0.011	r = 0.32	0.0125	Yes	Manuscript Table 2 P value/effect size
Knee joint power	Dual-task walking	0.56 (0.41, 0.66)	0.55 (0.43, 0.89)	Mann–Whitney U	|Z| = 0.482	—	0.630	r = 0.06	0.0125	No	Manuscript Table 2 P value/effect size
Knee joint power	Balance-challenged walking	0.60 (0.44, 0.83)	0.45 (0.31, 1.06)	Mann–Whitney U	|Z| = 1.227	—	0.220	r = 0.15	0.007	No	Manuscript Table 2 P value/effect size
**Spatiotemporal Parameters (Balance-challenged walking only)**											
Cadence (steps/min)	Balance-challenged	95.71 ± 10.81	106.77 ± 10.77	Independent t-test	t = -4.10	62	< .001	Cohen's d = 1.03 (large)	0.007	Yes	Recalculated from Manuscript Table 2 summaries
Stride length (m)	Balance-challenged	1.23 ± 0.06	1.30 ± 0.06	Independent t-test	t = -4.67	62	< .001	Cohen's d = 1.23 (large)	0.007	Yes	Recalculated from Manuscript Table 2 summaries
Walking speed (m/s)	Balance-challenged	0.92 (0.89, 1.06)	1.20 (1.10, 1.27)	Mann–Whitney U	|Z| = 4.72	—	< .001	r = 0.59 (large)	0.007	Yes	Manuscript Table 2 P value/effect size

Statistical details for manuscript Table 3 — Cortical activation (fNIRS, ΔHbO_2)

Channel	Brain region	Condition	LFO (n=32)	HFO (n=32)	Test	Test statistic	df	Exact P	Effect size (95% CI)	Bonferroni α (.05/35)	Significant after correction
**Sensorimotor cortex (BA 1/2/3, 4, 6) — motor planning and execution**											
S6D5	Primary somatosensory (BA 1/2/3)	Normal walking	-0.0136 ± 0.0196	0.0069 ± 0.0292	Independent t-test	t = -3.288	62	0.0020	Cohen's d = -0.822 (-1.330, -0.308)	0.00143	No
S6D5	Primary somatosensory (BA 1/2/3)	Dual-task walking	-0.0080 ± 0.0312	0.0293 ± 0.0464	Independent t-test	t = -3.767	62	< .001	Cohen's d = -0.942 (-1.455, -0.421)	0.00143	Yes
S6D5	Primary somatosensory (BA 1/2/3)	Balance-challenged walking	-0.0046 ± 0.0898	0.0193 ± 0.0522	Independent t-test	t = -1.302	62	0.1980	Cohen's d = -0.326 (-0.818, 0.169)	0.00143	No
S6D11	BA 3/1/6	Normal walking	-0.0221 ± 0.0277	0.0058 ± 0.0277	Independent t-test	t = -4.021	62	< .001	Cohen's d = -1.005 (-1.522, -0.481)	0.00143	Yes
S6D11	BA 3/1/6	Dual-task walking	0.0072 ± 0.0510	0.0223 ± 0.0419	Independent t-test	t = -1.293	62	0.2010	Cohen's d = -0.323 (-0.815, 0.171)	0.00143	No
S6D11	BA 3/1/6	Balance-challenged walking	0.0169 ± 0.0692	0.0194 ± 0.0443	Independent t-test	t = -0.175	62	0.8620	Cohen's d = -0.044 (-0.534, 0.446)	0.00143	No
S7D1	Supplementary motor area (BA 6)	Normal walking	-0.0093 ± 0.0272	0.0072 ± 0.0214	Independent t-test	t = -2.703	62	0.0090	Cohen's d = -0.676 (-1.177, -0.169)	0.00143	No
S7D1	Supplementary motor area (BA 6)	Dual-task walking	0.0114 ± 0.0541	0.0143 ± 0.0355	Independent t-test	t = -0.250	62	0.8030	Cohen's d = -0.063 (-0.552, 0.428)	0.00143	No
S7D1	Supplementary motor area (BA 6)	Balance-challenged walking	-0.0077 ± 0.0321	0.0164 ± 0.0407	Independent t-test	t = -2.631	62	0.0110	Cohen's d = -0.658 (-1.159, -0.152)	0.00143	No
S14D11	Premotor cortex (BA 6)	Normal walking	-0.0203 ± 0.0247	0.0023 ± 0.0338	Independent t-test	t = -3.063	62	0.0030	Cohen's d = -0.766 (-1.271, -0.255)	0.00143	No
S14D11	Premotor cortex (BA 6)	Dual-task walking	-0.0017 ± 0.0364	0.0125 ± 0.0314	Independent t-test	t = -1.669	62	0.1000	Cohen's d = -0.417 (-0.911, 0.080)	0.00143	No
S14D11	Premotor cortex (BA 6)	Balance-challenged walking	-0.0068 ± 0.0355	0.0187 ± 0.0376	Independent t-test	t = -2.790	62	0.0070	Cohen's d = -0.698 (-1.200, -0.190)	0.00143	No
S11D5	Premotor cortex (BA 6)	Normal walking	-0.0030 ± 0.0178	0.0011 ± 0.0301	Independent t-test	t = -0.658	62	0.5130	Cohen's d = -0.165 (-0.655, 0.327)	0.00143	No
S11D5	Premotor cortex (BA 6)	Dual-task walking	0.0023 ± 0.0494	0.0075 ± 0.0236	Independent t-test	t = -0.527	62	0.6000	Cohen's d = -0.132 (-0.622, 0.359)	0.00143	No
S11D5	Premotor cortex (BA 6)	Balance-challenged walking	-0.0081 ± 0.0362	0.0018 ± 0.0320	Independent t-test	t = -1.163	62	0.2490	Cohen's d = -0.291 (-0.782, 0.203)	0.00143	No
**Prefrontal cortex (BA 9/10/46) — executive function and cognitive control**											
S3D2	Polar frontal area (BA 10)	Normal walking	0.0214 ± 0.0410	-0.0012 ± 0.0395	Independent t-test	t = 2.242	62	0.0290	Cohen's d = 0.560 (0.059, 1.058)	0.00143	No
S3D3	Polar frontal area (BA 10)	Dual-task walking	0.0034 ± 0.0383	0.0485 ± 0.0567	Independent t-test	t = -3.730	62	< .001	Cohen's d = -0.932 (-1.446, -0.412)	0.00143	Yes
S3D3	Polar frontal area (BA 10)	Balance-challenged walking	0.0019 ± 0.1161	0.0344 ± 0.0584	Independent t-test	t = -1.412	62	0.1630	Cohen's d = -0.353 (-0.846, 0.142)	0.00143	No
S8D8	DLPFC (BA 9/46)	Normal walking	0.0023 ± 0.0324	0.0054 ± 0.0435	Independent t-test	t = -0.319	62	0.7510	Cohen's d = -0.080 (-0.570, 0.411)	0.00143	No
S8D8	DLPFC (BA 9/46)	Dual-task walking	0.0014 ± 0.0647	-0.0028 ± 0.0691	Independent t-test	t = 0.252	62	0.8020	Cohen's d = 0.063 (-0.427, 0.553)	0.00143	No
S8D8	DLPFC (BA 9/46)	Balance-challenged walking	-0.0252 ± 0.0646	0.0320 ± 0.0413	Independent t-test	t = -4.221	62	< .001	Cohen's d = -1.055 (-1.575, -0.528)	0.00143	Yes

Channel	Brain region	Condition	LFO (n=32)	HFO (n=32)	Test	Test statistic	df	Exact P	Effect size (95% CI)	Bonferroni α (.05/35)	Significant after correction
S9D9	DLPFC (BA 9/46)	Normal walking	0.0082 ± 0.0217	0.0039 ± 0.0297	Independent t-test	t = 0.662	62	0.5110	Cohen's d = 0.165 (-0.326, 0.656)	0.00143	No
S9D9	DLPFC (BA 9/46)	Dual-task walking	0.0276 ± 0.1383	0.0152 ± 0.0358	Independent t-test	t = 0.491	62	0.6250	Cohen's d = 0.123 (-0.368, 0.613)	0.00143	No
S9D9	DLPFC (BA 9/46)	Balance-challenged walking	-0.0206 ± 0.0509	0.0186 ± 0.0404	Independent t-test	t = -3.410	62	0.0010	Cohen's d = -0.853 (-1.362, -0.337)	0.00143	Yes

Statistical details for manuscript Table 4 — Muscle co-contraction indices

Muscle pair	Side	Condition	LFO (n=32)	HFO (n=32)	Test	Test statistic	df	Exact P	Effect size (95% CI)	Bonferroni α (.05/8)	Significant after correction
**Ankle joint — TA–GL antagonist co-contraction**											
TA–GL	Affected	Normal walking	0.591 ± 0.119	0.747 ± 0.136	Independent t-test	t = -4.906	62	< .001	Cohen's d = 1.23	0.006	Yes
TA–GL	Affected	Dual-task walking	0.590 ± 0.176	0.654 ± 0.146	Independent t-test	t = -1.577	62	0.120	Cohen's d = 0.39	0.006	No
TA–GL	Affected	Balance-challenged walking	0.628 ± 0.139	0.742 ± 0.179	Independent t-test	t = -2.831	62	0.006	Cohen's d = 0.71	0.006	Yes (at boundary)
TA–GL	Unaffected	Normal walking	0.592 ± 0.207	0.581 ± 0.198	Independent t-test	t = 0.224	62	0.823	Cohen's d = 0.06	0.006	No
TA–GL	Unaffected	Dual-task walking	0.497 ± 0.265	0.582 ± 0.198	Independent t-test	t = -1.465	62	0.148	Cohen's d = 0.37	0.006	No
TA–GL	Unaffected	Balance-challenged walking	0.594 ± 0.249	0.588 ± 0.154	Independent t-test	t = 0.101	62	0.920	Cohen's d = 0.03	0.006	No
**Knee joint — RF–Ham antagonist co-contraction**											
RF–Ham	Affected	Normal walking	0.657 ± 0.161	0.612 ± 0.209	Independent t-test	t = 0.961	62	0.340	Cohen's d = 0.24	0.006	No
RF–Ham	Affected	Dual-task walking	0.699 ± 0.161	0.701 ± 0.137	Independent t-test	t = -0.041	62	0.967	Cohen's d = 0.01	0.006	No
RF–Ham	Affected	Balance-challenged walking	0.402 ± 0.174	0.544 ± 0.144	Independent t-test	t = -3.542	62	0.001	Cohen's d = 0.89	0.006	Yes
RF–Ham	Unaffected	Normal walking	0.724 ± 0.213	0.787 ± 0.170	Independent t-test	t = -1.298	62	0.199	Cohen's d = 0.33	0.006	No
RF–Ham	Unaffected	Dual-task walking	0.578 ± 0.202	0.709 ± 0.148	Independent t-test	t = -2.959	62	0.004	Cohen's d = 0.74	0.006	Yes
RF–Ham	Unaffected	Balance-challenged walking	0.750 ± 0.155	0.830 ± 0.131	Independent t-test	t = -2.228	62	0.030	Cohen's d = 0.56	0.006	No

Statistical details for manuscript Table 5 — Brain–muscle coupling correlations

Channel	Brain region	Muscle pair	Side	Condition	Test (correlation)	Coefficient	n	Uncorrected P	Survives FDR (q < .05 across 140 pairs)?
**Balance-challenged walking — Lower Functional Outcome group**									
S6–D5	SMC (BA 1/2/3)	RF–Ham	Unaffected	Balance-challenged	Pearson r	0.487	32	0.005	No

Channel	Brain region	Muscle pair	Side	Condition	Test (correlation)	Coefficient	n	Uncorrected P	Survives FDR (q < .05 across 140 pairs)?
S11–D5	PMC (BA 6)	RF–Ham	Unaffected	Balance-challenged	Pearson r	0.466	32	0.007	No
S3–D3	PFA (BA 10)	RF–Ham	Affected	Balance-challenged	Pearson r	−0.460	32	0.008	No
S13–D14	PMC (BA 6)	RF–Ham	Unaffected	Balance-challenged	Pearson r	0.440	32	0.012	No
S7–D1	SMA (BA 6)	TA–GL	Affected	Balance-challenged	Pearson r	0.431	32	0.014	No
**Normal walking — Lower Functional Outcome group**									
S2–D7	IFA (BA 45)	TA–GL	Unaffected	Normal walking	Pearson r	−0.504	32	0.003	No
S6–D5	SMC (BA 1/2/3)	RF–Ham	Unaffected	Normal walking	Pearson r	0.473	32	0.006	No
S11–D5	PMC (BA 6)	TA–GL	Affected	Normal walking	Pearson r	0.473	32	0.006	No
**Dual-task walking — Lower Functional Outcome group**									
S11–D11	PMC (BA 6)	RF–Ham	Affected	Dual-task walking	Pearson r	0.460	32	0.008	No

Figure 3 — statistical underlay

Figure 3 visualizes the data analyzed in Table 4 (muscle co-contraction indices). For per-bar test details, see the 'Table4_CCI' worksheet. Asterisks shown above bracketed bar pairs in Figure 3 correspond to: *P < .05; **P < .01; ***P < .001 (all uncorrected); bracketed pairs that remain significant after Bonferroni correction within condition (α = .006) are flagged in the 'Significant after correction' column of the Table4_CCI sheet.

Figure 4 — statistical underlay

Figure 4 visualizes the brain–muscle coupling correlations in the lower-function group during balance-challenged walking, listed in detail in the 'Table5_BrainMuscle' worksheet (top section). Correlation method was determined channel-by-channel by the Shapiro–Wilk test on the joint distribution (Pearson r vs. Spearman ρ). Gold-bordered cells in Figure 4 mark uncorrected P < .05; *P < .05, **P < .01 as labelled within the heatmap. None of the correlations survived FDR (Benjamini–Hochberg, q < .05) across 140 channel × muscle pairs per condition; the analysis is reported as exploratory.
